# Agrarian and $\ell ^2$-Betti numbers of locally indicable groups, with a twist

**DOI:** 10.1007/s00208-024-02835-7

**Published:** 2024-03-26

**Authors:** Dawid Kielak, Bin Sun

**Affiliations:** 1 Mathematical Institute, University of Oxford, Andrew Wiles Building, Radcliffe Observatory Quarter, Woodstock Road, Oxford, OX2 6GG UK; 2 Present Address: 619 Red Cedar Road C212, Wells Hall, East Lansing, MI 48824 USA

## Abstract

We prove that twisted $\ell ^2$-Betti numbers of locally indicable groups are equal to the usual $\ell ^2$-Betti numbers rescaled by the dimension of the twisting representation; this answers a question of Lück for this class of groups. It also leads to two formulae: given a fibration *E* with base space *B* having locally indicable fundamental group, and with a simply-connected fiber *F*, the first formula bounds $\ell ^2$-Betti numbers $b_i^{(2)}(E)$ of *E* in terms of $\ell ^2$-Betti numbers of *B* and usual Betti numbers of *F*; the second formula computes $b_i^{(2)}(E)$ exactly in terms of the same data, provided that *F* is a high-dimensional sphere. We also present an inequality between twisted Alexander and Thurston norms for free-by-cyclic groups and 3-manifolds. The technical tools we use come from the theory of generalised agrarian invariants, whose study we initiate in this paper.

## Introduction

Motivated by the Gauss–Bonnet theorem, and seeking *“Zusammenhänge und Bindungen [...] zwischen den topologischen Eigenschaften einerseits und den differentialgeometrischen Eigenschaften andererseits”*, Heinz Hopf formulated in 1932 [20, Page 224] a somewhat vague question about the relationship between curvature of even-dimensional Riemannian manifolds and their Euler characteristic. The question was then given an explicit form, now known as the Hopf Conjecture. The version of the conjecture for negatively curved manifolds can be found in the work of Yau [50, Problem 10]), and states: every closed Riemannian 2*n*-manifold *M* of negative sectional curvature satisfies $(-1)^n\chi (M)>0$, where $\chi (M)$ denotes the Euler characteristic of *M*.

Since Riemannian manifolds of negative sectional curvature are aspherical, one can replace the geometric assumption by a topological one. This was done by Thurston (see [29, Problem 4.10]), who formulated the following conjecture: every closed aspherical 2*n*-dimensional manifold *M* satisfies $(-1)^n\chi (M)\geqslant 0$.

An attack strategy for resolving Hopf Conjecture was proposed by Singer, as reported by Dodziuk [10, Conjecture 2] and Yau [50]. What is now known as the Singer Conjecture states that the $\ell ^2$-Betti numbers $b_i^{(2)}(M)$ of a closed aspherical *n*-manifold *M* should vanish in all dimensions, except perhaps the middle dimension $\frac{n}{2}$, if it exists; moreover, if the manifold is negatively curved, then the middle $\ell ^2$-Betti number should be strictly positive. Knowing the $\ell ^2$-Betti numbers allows one to compute the $\ell ^2$-Euler characteristic, which for manifolds is equal to the usual Euler characteristic by the $L^2$ Index Theorem of Atiyah [3]. Therefore, the Singer Conjecture implies Hopf and Thurston Conjectures. Furthermore, Singer Conjecture was established for locally symmetric spaces by Borel [5] (see also [42]), a large class of manifolds of classical interest.

Singer Conjecture is a statement about aspherical manifolds. But such manifolds can be used as building blocks in constructions of more general spaces. One example of this is a fibration over an aspherical manifold. If one could connect the $\ell ^2$-Betti numbers of such fibrations with those of the aspherical base spaces, then, on the one hand, the Singer conjecture would yield a statement about $\ell ^2$-Betti numbers of some not necessarily aspherical manifolds, and on the other hand, the conjecture could potentially be disproved by finding a suitable not necessarily aspherical example.

In this article we give a method of computing $\ell ^2$-Betti numbers for fibrations $F\rightarrow E \rightarrow B$ when *F* is sufficiently similar to a high-dimensional sphere, and when $\pi _1(B)$ is virtually locally indicable. Recall that a group *G* is *locally indicable* if every non-trivial finitely generated subgroup admits an epimorphism onto $\mathbb {Z}$. A group *G* is *virtually locally indicable* if *G* has a finite-index locally indicable subgroup.

### Theorem 1.1

Let $F\rightarrow E \rightarrow B$ be a fibration of connected finite CW-complexes, with $\pi _1(B)$ being virtually locally indicable. If *F* is simply connected, or more generally, if the map $\pi _1(E)\rightarrow \pi _1(B)$ induced by the fibration is an isomorphism, then

$$\begin{aligned} b^{(2)}_i(E)\leqslant \sum ^i_{j=0}b_j(F)\cdot b^{(2)}_{i-j}(B) \end{aligned}$$

for every $$i \in {\mathbb {N}}$$.

If moreover the homology of *F* with $\mathbb {C}$-coefficients is non-zero in at most two degrees, 0 and *n* with $n\geqslant \max \{2, \dim B \}$ (e.g., *F* is a sphere of dimension at least 2), then for every $i \in {\mathbb {N}}$ we have

$$\begin{aligned} b^{(2)}_i(E)=b^{(2)}_i(B)+b_n(F)\cdot b^{(2)}_{i-n}(B).\end{aligned}$$

In fact, we prove a more general result, Theorem 4.1, whose statement is perhaps too involved for this introduction; Theorem 1.1 summarises items (i) and (ii) of Theorem 4.1. Also, Theorem 4.1 makes the connection between Theorem 1.1 and Singer Conjecture explicit.

Using techniques similar to those underpinning Theorem 1.1, in Sect. 4 we will prove the following results.

### Corollary 1.2

Let $F\rightarrow E \rightarrow B$ be a fiber bundle of compact connected manifolds such that *F* is simply connected and *B* is a surface (possibly with boundary) with $|\pi _1(B)|=\infty$ (which holds except when *B* is either $S^2$, the 2-disk, or the projective plane $P^2$). Then for every $i \in {\mathbb {N}}$ we have

$$\begin{aligned} b^{(2)}_i(E)=-\chi (B)b_{i-1}(F). \end{aligned}$$

### Corollary 1.3

Let $F\rightarrow E \rightarrow B$ be a fiber bundle of compact connected manifolds such that *F* is simply connected and *B* is an orientable prime 3-manifold with empty or toroidal boundary and infinite fundamental group. Then $b^{(2)}_{*}(E)=0$.

In the setting of all of the above results, the $\ell ^2$-homology of *E* is related via the Leray–Serre spectral sequence to the homology of *B* with $\ell ^2$-coefficients twisted by the action of $\pi _1(B)$ on the usual $\mathbb {C}$-homology of *F*, as noticed by Lück [39] (see Sect. 4). Our proof of Theorem 4.1 thus follows from relating the *twisted* $\ell ^2$*-Betti number* of *B* to $b^{(2)}_{*}(B)$.

In fact, the question of the nature of twisted $\ell ^2$-Betti numbers can be asked more generally, in the realm of group theory; in particular, for a group *G* and an *n*-dimensional complex representation $\sigma$, Lück asked whether the $\ell ^2$-Betti numbers of *G* twisted by $\sigma$ are equal to the corresponding usual $\ell ^2$-Betti numbers multiplied by *n* (see Question 4.2). In Theorem 4.4 we prove that this is indeed the case when *G* is locally indicable.

After the first version of this article appeared online, Boschheidgen–Jaikin-Zapirain in [4] related the twisted and untwisted $\ell ^2$-Betti numbers for sofic groups. We remark that it is unclear whether the main result of [4] generalises our Theorem 4.4 as it is currently unknown if locally indicable groups are sofic.

Another setting in which one wishes to twist homology with finite-dimensional representations is that of knots and 3-manifolds. Here one studies for example the twisted Alexander polynomial, which was introduced as a refined version of the classical Alexander polynomial, by Lin [34] for knot groups and Wada [49] for general finitely presented groups. (Note that although Lin’s paper was published later, it was finished in 1990, and thus the introduction of the twisted Alexander polynomial is attributed to both Lin and Wada.) It is used to distinguish certain knots from their inversions [31]. Twisted Alexander polynomials can also detect fiberedness of characters, as proven by Friedl–Vidussi [16]. This fact is crucially used in two important recent results of Jaikin-Zapirain [24] and Liu [36]. We refer the reader to [17] for a survey on this topic.

Every twisted Alexander polynomial leads to a *twisted Alexander norm*, a function $H^1(G,\mathbb {R}) \rightarrow [0,\infty )$. The $\ell ^2$-analogue of this for locally indicable groups is the Thurston norm $\Vert \cdot \Vert _T:H^1(G,\mathbb {R}) \rightarrow [0,\infty )$. (This terminology is justified, since this latter norm coincides with Thurston’s famous norm [48] in the case of 3-manifolds, as proven by Friedl–Lück [15] – see Example 3.17.)

The classical Thurston norm on a 3-manifold *M* is closely related to fibrations of *M* over the circle $S^1$: the unit ball *B* of $\Vert \cdot \Vert _T$ is a polytope and there exists a collection of top dimensional faces of *B* such that a character $\phi \in H^1(M,\mathbb {Z})$ induces such a fibration if and only if $\phi$ belongs to the interior of the positive cone of one of those top dimensional faces [48, Theorem 3]. The same statement also holds for free-by-cyclic groups [27, Theorem 5.29].

Despite of its significance, a disadvantage of the Thurston norm is that it is hard to compute in general. In the seminal paper [41], McMullen showed that for 3-manifolds, the Alexander norm, which is computationally much easier, provides a lower bound to the Thurston norm. McMullen’s result was extended in [13] to the case of free-by-cyclic groups. We extend these results further to cover twisted Alexander norms:

### Theorem 1.4

(Theorems 7.1 and 7.2) Let *G* be either

- a (finitely generated free)-by-(infinite cyclic) group, or
- the fundamental group of a closed connected orientable 3-manifold that fibers over $S^1$.

Then for every finite-dimensional representation $\sigma :G\rightarrow {{\,\textrm{GL}\,}}_n(\mathbb {C})$ and every character $\phi \in H^1(G,\mathbb {Z})$ we have

$$\begin{aligned}\Vert \phi \Vert _{\sigma }\leqslant n\cdot \Vert \phi \Vert _T,\end{aligned}$$

where $\Vert \phi \Vert _{\sigma }$ (resp. $\Vert \phi \Vert _T$) denotes the twisted Alexander norm of $\phi$ with respect to $\sigma$ (resp. the Thurston norm of $\phi$). Moreover, equality holds when $\phi$ is a *fibered character*, i.e., when $\ker \phi$ is finitely generated.

The 3-manifold case of the above theorem was first proved by Friedl–Kim [12] by a different method.

Both the Alexander polynomial and $\ell ^2$-homology form part of the unified theory of agrarian invariants; the same holds true for their twisted analogues – both twisted $\ell ^2$-homology and twisted Alexander polynomials are manifestations of *agrarian invariants* of a generalized kind, whose study we initiate here.

**Outline of the paper.** We recall the necessary definitions and results in Sect. 2. We then introduce agrarian invariants (of a generalized kind) in Sect. 3. We study twisted $\ell ^2$-Betti numbers in Sect. 4, where Theorem 1.1 and Corollaries 1.2, 1.3 are proved. The rest of the paper, i.e., Sects. 5 through 7, are devoted to the study of the Thurston and twisted Alexander norms.

## Preliminaries

### Twisted group rings

We now recall the construction of twisted group rings, which will be used in dealing with twisted $\ell ^2$-Betti numbers.

#### Definition 2.1

Let *R* be an associative ring with unity. Denote the group of units of *R* by $R^{\times }$. Let *G* be a group and let

$$\begin{aligned} c&:G\rightarrow \textrm{Aut}(R), g\mapsto c_g,\\ \tau&:G\times G\rightarrow R^{\times }, (g,g')\mapsto \tau (g,g') \end{aligned}$$

be functions such that

$$\begin{aligned} c_g(c_{g'}(r))=\tau (g,g')\cdot c_{gg'}(r)\cdot \tau (g,g')^{-1},\\ \tau (g,g')\tau (gg',g'')=c_g(\tau (g',g''))\cdot \tau (g,g'g''), \end{aligned}$$

where $g,g',g''\in G$ and $r\in R$. The pair $(c,\tau )$ is the pair of *structure functions*. We denote by *RG* the free *R*-module with basis *G* and write elements of *RG* as finite *R*-linear combinations $\sum _{g\in G}r_g*g$ of elements of *g*. When convenient, we shorten $1*g$ to *g*. The structure functions endow *RG* with the structure of an (associative) *twisted group ring* by declaring

$$\begin{aligned} (r*g)\cdot (r'*g')=(r\cdot c_g(r')\cdot \tau (g,g'))*(gg') \end{aligned}$$

and extending linearly. For details see [43, Section 1.2].

If $c_g=\textrm{id}_R$ and $\tau (g,g')=1$ for all $g,g'\in G$, in which case we say the structure functions $(c,\tau )$ are *trivial*, then *RG* is called the *untwisted group ring*, in which case we will write elements of *RG* as $\sum _{g\in G} rg$ instead of $\sum _{g\in G}r*g$, and we will also shorten $r*1$ to *r*.

Note that $\tau (1,1)^{-1}*1$ is the multiplicative identity of the twisted group ring.

#### Notation 2.2

We will mostly use the notation *RG* for a twisted group ring. However, in Sect. 3.2 we will talk about two group ring structures on *RG*, one twisted and the other one untwisted. There, we will denote the twisted group ring by $R*G$ and the untwisted one by *RG*.

In the sequel, we reserve the name group rings for untwisted group rings.

Since all our rings are unital, we require ring homomorphisms to respect units.

#### Example 2.3

Let $\phi :G\rightarrow H$ be a surjective group homomorphism with kernel *K*. We choose a set-theoretic section $s:H\rightarrow G$, i.e., a map between the underlying sets such that $\phi \circ s=\textrm{id}_H$. Let $(\mathbb {Z}K) H$ be the twisted group ring with structure functions $c_h(r)=s(h)rs(h)^{-1}$ and $\tau (h,h')=s(h)s(h')s(hh')^{-1}$. The untwisted group ring $\mathbb {Z}G$ is then isomorphic to the twisted group ring $(\mathbb {Z}K) H$ via the map

$$\begin{aligned}g\mapsto (g(s\circ \phi )(g)^{-1})*\phi (g).\end{aligned}$$

### Ore localization

The notion of the Ore localization is a generalization of the classical notion of the field of fractions of an integral domain. We will use this notion to rationalize a given agrarian map in Sect. 3.2.

Let *R* be a ring and let $T\subset R$ be a subset of *R* that does not include any zero-divisors. We say *T* satisfies the *left Ore condition* if for all $r\in R$ and $t\in T$, there exist $r_1\in R,t_1\in T$ such that $r_1t=t_1r$. The *(left) Ore localization of* *R* *with respect to* *T* is

$$\begin{aligned}{{\,\textrm{Ore}\,}}(R,T)=\{t^{-1}r\mid t\in T, r\in R\}.\end{aligned}$$

It is a well defined ring - for details, see [43, Theorem 4.1] and its proof.

If *R* has no non-trivial zero divisor, *T* is the set of non-zero elements of *R*, and *T* satisfies the left Ore condition, then we briefly say that *R* *satisfies the Ore condition* and call the Ore localization of *R* with respect to *T* the *Ore localization* of *R*; we denote it by ${{\,\textrm{Ore}\,}}(R)$, and note that it is a skew field (division ring).

#### Example 2.4

Let *D* be a skew field and let *H* be a torsion-free amenable group. Every twisted group ring *DH* that is a domain satisfies the Ore condition – this follows from [46], and in this form is stated for example in [27, Theorem 2.14].

### Laurent power series and orders

Let *R* be a ring, let $\alpha$ be an automorphism of *R*, and let $t\not \in R$ be a symbol. The ring of *twisted Laurent power series* in *t* with coefficients in *R* is the set

$$\begin{aligned} R(\!(t)\!)=\left\{ \sum ^{\infty }_{i=k}r_it^i\mid k\in \mathbb {Z}, r_i\in R\right\} . \end{aligned}$$

Our convention is $r_i=0$ for $i<k$.

The multiplication on $R(\!(t)\!)$ is given by the convention

$$\begin{aligned} t\cdot r=\alpha (r)\cdot t. \end{aligned}$$

With this multiplication and the obvious summation $R(\!(t)\!)$ is a ring. The function $\alpha$ is called the *twisting structure* of $R(\!(t)\!)$. If $\alpha =\textrm{id}_R$ then $R(\!(t)\!)$ will be called the *ring of (untwisted) Laurent power series* and *t* will be called a *central variable*.

#### Remark 2.5

Let $x=\sum ^{\infty }_{i=k}r_it^i\in R(\!(t)\!)$. If $r_k$ is invertible in *R*, then *x* is invertible in $R(\!(t)\!)$. Indeed, the inverse of *x* can be found by solving linear equations.

For the rest of this subsection we restrict ourselves to the case where $R=D$ is a skew field and $\alpha =\textrm{id}_D$, in which case $D(\!(t)\!)$ is a skew field.

For each $x= \sum ^{\infty }_{i=k}d_i t^i \in D(\!(t)\!)$, if $d_k \ne 0$, then we define the *order* of *t* in *x* as

$$\begin{aligned}{{\,\textrm{ord}\,}}_t x = k;\end{aligned}$$

and if $x=0$, then ${{\,\textrm{ord}\,}}_t x = \infty$ by definition. It is easy to check that if $x,y\in D(\!(t)\!)\smallsetminus \{0\}$, then

$$\begin{aligned}{{\,\textrm{ord}\,}}_t (xy)={{\,\textrm{ord}\,}}_t x + {{\,\textrm{ord}\,}}_t y.\end{aligned}$$

So ${{\,\textrm{ord}\,}}_t$ restricts to a homomorphism ${{\,\textrm{ord}\,}}_t :D(\!(t)\!)_{\textrm{ab}}^{\times }\rightarrow \mathbb {Z}$, where $D(\!(t)\!)_{\textrm{ab}}^{\times }$ denotes the abelianization of the group of units $D(\!(t)\!)^\times$ of $D(\!(t)\!)$. Taking 0 into account, we can also view ${{\,\textrm{ord}\,}}_t$ as a semi-group homomorphism

$$\begin{aligned} {{\,\textrm{ord}\,}}_t:D(\!(t)\!)_{\textrm{ab}}^{\times } \sqcup \{0\}\rightarrow \mathbb {Z}\sqcup \{\infty \}. \end{aligned}$$

Here our convention is $\infty +n=\infty$ for all $n\in \mathbb {Z}\sqcup \{\infty \}$.

### Degree of rational functions

The notion of the degree serves as a convenient way to compute the Thurston norm and, more generally, the agrarian norm. Let *D* be a skew field, let *H* be a finite-rank free abelian group with basis *X*, and let *DH* be the untwisted group ring. Note that *DH* is a domain and satisfies the Ore condition by Example 2.4.

Let $t\in X$. We would like to define the *t*-degree function in this subsection, which depends on the choice of *X*. We thus use the following convention to emphasize the role played by *X*.

#### Notation 2.6

We will denote *DH* by $D[X^{\pm }]$ and the Ore localization ${{\,\textrm{Ore}\,}}(DH)$ by *D*(*X*).

For each $p\in D[X^{\pm }]\smallsetminus \{0\}$, we defined the *degree* of *t* in *p*, denoted $\deg _t p$, as the maximal power of *t* in *p* minus the minimal power of *t* in *p*. Note that $\deg _t$ is not an extension of the usual notion of the degree of a polynomial. For $p=0$, we define $\deg _t p=-\infty$. The degree function can be extended to *D*(*X*): for every element $f\in D(X)$, the *degree* of *t* in *f*, denoted $\deg _t f$, is

$$\begin{aligned}\deg _t f = \deg _t p - \deg _t q,\end{aligned}$$

where $p,q\in DH,q\ne 0$ and $q^{-1}p=f$. Here, our convention is $-\infty +n=-\infty$ for all $n\in \mathbb {Z}$. The well-definedness of $\deg _t f$ is an easy exercise using the Ore condition.

We define an *order function* ${{\,\textrm{ord}\,}}_t$ *with respect to the basis* *X* by embedding *D*(*X*) into a Laurent series ring and using Sect. 2.4. First, let *K* be the subgroup of *H* generated by $X\smallsetminus \{t\}$ and let $E={{\,\textrm{Ore}\,}}(DK)$. Then we have a natural map $\alpha :D(X)\hookrightarrow E(\!(t)\!)$ by expanding every rational function into a Laurent power series: Let $f\in E[t]\smallsetminus \{0\}\subset D(X)$. Factorize *f* as $f=d t^k\cdot (1+\sum ^{\ell }_{i=1}d_i t^i)$, where $d,d_i\in E,d\ne 0$. Define

$$\begin{aligned} \alpha (f^{-1})=\left( 1+\sum ^{\infty }_{j=1}\left( -\sum ^{\ell }_{i=1}d_i t^i\right) ^{j}\right) \cdot d^{-1}t^{-k}. \end{aligned}$$

Every element of *D*(*X*) can be written as a fraction $q^{-1}p$ with $p,q\in E[t],q\ne 0$. Define

$$\begin{aligned}\alpha (q^{-1}p)=\alpha (q^{-1})\cdot p.\end{aligned}$$

That $\alpha$ is well defined follows from the universal property of the Ore localization.

The function $\alpha$ embeds *D*(*X*) into $E(\!(t)\!)$ as a subfield, and so we can view each element $f\in D(X)$ as an element of $E(\!(t)\!)$ and compute ${{\,\textrm{ord}\,}}_t f$. Once again, this *t*-order depends on the basis *X*.

There is a ring homomorphism $\beta :E[t^{\pm }]\rightarrow E[t^{\pm }]$ such that $\beta (e t^k)=e t^{-k}$ for all $e\in E,k\in \mathbb {Z}$. For $f\in D(X)$, let $p,q\in D[X]$ such that $f=q^{-1}p$ and define

$$\begin{aligned}\beta (f)=\beta (q)^{-1}\beta (p).\end{aligned}$$

That $\beta$ is well defined follows from the universal property of the Ore localization.

Thus, $\beta$ extends to a map (still denoted by) $\beta :E(t)\rightarrow E(t)$, which can be easily seen to be a ring homomorphism. Also, $\beta$ is the inverse of itself, so it is in fact a ring automorphism. We have

1 $$\begin{aligned} \deg _t f=-{{\,\textrm{ord}\,}}_t f-{{\,\textrm{ord}\,}}_t \beta (f). \end{aligned}$$

Just like ${{\,\textrm{ord}\,}}_t$, the function $\deg _t$ descends to a semi-group homomorphism

$$\begin{aligned} \deg _t:D(X)^{\times }_{\textrm{ab}}\sqcup \{0\}\rightarrow \mathbb {Z}\sqcup \{-\infty \}. \end{aligned}$$

### Dieudonné determinant

The Dieudonné determinant is a generalization to skew fields of the classical notion of determinant over a commutative field. It is indispensable in our definition of agrarian torsion and thus we recall its definition here. Let *D* be a skew field and let $A=(A_{ij})$ be an $n\times n$-matrix over *D*. The *canonical representative of the Dieudonné determinant* $\det ^c_D A\in D$ is defined inductively as follows:

1. If $$n=1$$, then $$\det ^c_D A=a_{11}$$.
2. If the last row of *A* consists of zeros only, then $\det ^c_D A=0$.
3. If $a_{nn}\ne 0$, then we form the $(n-1)\times (n-1)$-matrix $A'=(a'_{ij})$ by setting $a'_{ij}=a_{ij}-a_{in}a^{-1}_{nn}a_{nj}$ and declare $\det ^c_D A=\det ^c_D A'\cdot a_{nn}$.
4. Otherwise, let $j<n$ be maximal such that $a_{nj}\ne 0$. Let $A'$ be obtained from *A* by interchanging columns *j* and *n*. Then set $\det ^c_D A=-\det ^c_D A'$.

The *Dieudonné determinant* $\det _D A$ of *A* is defined to be the image of $\det ^c_D A$ in $D^{\times }_{\textrm{ab}}\sqcup \{0\}$, where $D^{\times }_{\textrm{ab}}$ is the abelianization of the multiplicative group of *D*.

#### Remark 2.7

The Dieudonné determinant satisfies the following [9]:

1. ${{\,\mathrm{\det }\,}}_D (AB)={{\,\mathrm{\det }\,}}_D A\cdot {{\,\mathrm{\det }\,}}_D B$ for all square matrices *A*, *B* of the same dimension.
2. If $A'$ is obtained from *A* by adding a multiple of a row to another row, then $\det _D A'=\det _D A$.
3. If *A* is upper-triangular, then $\det _D A$ is the image of $\prod ^n_{i=1} a_{ii}$ in $D^{\times }_{\textrm{ab}}\sqcup \{0\}$.
4. *A* is invertible over *D* if and only if ${{\,\mathrm{\det }\,}}_D(A) \ne 0$.

### Polytope group and polytope homomorphism

In this subsection, we recall the notion of the polytope homomorphism, which is used later to construct the agrarian polytope. Let *V* be an $\mathbb {R}$-vector space.

#### Definition 2.8

A *polytope* in *V* is the convex hull of finitely many points of *V*. Given two polytopes *P*, *Q*, the *Minkowski sum* of *P*, *Q*, denoted $P+Q$, is the polytope

$$\begin{aligned} \{p+q\mid p\in P, q\in Q\}. \end{aligned}$$

Let *H* be a finite-rank free abelian group. Below, we take the vector space *V* to be $H_1(H,\mathbb {R})\cong H\otimes _{\mathbb {Z}}\mathbb {R}$.

#### Definition 2.9

A polytope in $H_1(H,\mathbb {R})$ is called *integral* if it is the convex hull of some finite subset of the lattice $H\subset H_1(H,\mathbb {R})\cong H\otimes _{\mathbb {Z}}\mathbb {R}$. The *polytope group* $\mathcal {P}(H)$ of *H* is the abelian group generated by formal differences $P-Q$ of non-empty integral polytopes *P*, *Q* in $H_1(H,\mathbb {R})$ with addition $(P-Q)+(P'-Q')=(P+P')-(Q+Q')$ and relations $P-Q=P'-Q'$ if $P+P'=Q+Q'$.

The unit of the group is the one-vertex polytope $\{0\}$, which we will denote by 0. Also, instead of writing $P - 0$ for a polytope *P* we will simply write *P*, and every element of $\mathcal {P}(H)$ of this form will be referred to as a *single* polytope.

Let *D* be a skew field and let *DH* be the (untwisted) group ring.

#### Definition 2.10

The *Newton polytope* *P*(*p*) of an element $p=\sum _{h\in H}d_h h\in DH$ is the convex hull of the *support* ${{\,\textrm{supp}\,}}(p)=\{h\in H\mid d_h\ne 0\}$ in $H_1(H,\mathbb {R})$.

#### Definition 2.11

The group homomorphism

$$\begin{aligned}P:({{\,\textrm{Ore}\,}}(DH))^{\times }_{\textrm{ab}}\rightarrow \mathcal {P}(H),~P(q^{-1}p)=P(p)-P(q)\end{aligned}$$

is called the *polytope homomorphism* of *DH*.

The well-definedness of *P* is an easy exercise using the Ore condition. That *P* is a group homomorphism is proved in [27, Lemma 3.12].

The following is a special case of [27, Theorem 3.14].

#### Theorem 2.12

Let *A* be a square matrix over *DH* with ${{\,\mathrm{\det }\,}}_{{{\,\textrm{Ore}\,}}(DH)}(A)\ne 0$. Then $P({{\,\mathrm{\det }\,}}_{{{\,\textrm{Ore}\,}}(DH)}(A))$ is a single polytope.

### A lemma about matrices over skew fields

In this subsection, we prove a technical lemma about matrices, which will be used several times in the sequel. Let *D* be a skew field and let *t* be a central variable. Let $D(\!(t)\!)$ be the ring of Laurent power series of *t* with coefficient in *D*. Consider a matrix *M* of the form

$$\begin{aligned}M=\textrm{Id}+N\cdot t,\end{aligned}$$

where $N=(n_{ij})\in M_n(D(\!(t)\!))$ and each entry of *N* has *t*-order at least 0, i.e., ${{\,\textrm{ord}\,}}_t n_{ij}\geqslant 0$ for all *i*, *j*. In order to compute $\det _{D(\!(t)\!)}M$, we use the following process to turn *M* into an upper-triangular matrix:

- $(*)$ This process consists of *n* steps. In the ${i}^{\textrm{th}}$ step, we use elementary row operations to eliminate all (*j*, *i*)-entries with $j>i$. In other words, for each $j>i$, we add a suitable left multiple of the ${i}^{\textrm{th}}$ row to the ${j}^{\textrm{th}}$ row so that the resulting matrix has 0 as its (*j*, *i*)-entry.

Note that in order to carry out the $(*)$ process, at the $i^{\textrm{th}}$ step we need the resulting matrix to have a non-zero (*i*, *i*)-entry, which is a priori unclear and thus it is a priori unclear whether the *n* steps in process $(*)$ can all be carried out. The following lemma affirms the feasibility of $(*)$.

#### Lemma 2.13

After each step of process $(*)$, we will get a matrix $M'=\textrm{Id}+N'\cdot t$, where each entry of $N'$ has *t*-order at least 0. In particular, $M'$ has non-zero diagonal entries, and thus all *n* steps of process $(*)$ can be carried out and the matrix obtained from process $(*)$, say ${\overline{M}}=({\overline{m}}_{ij})$, is upper-triangular and we have for all *i*, ${\overline{m}}_{ii}=1+{\overline{n}}_{ii}\cdot t$, where ${{\,\textrm{ord}\,}}_t {\overline{n}}_{ii}\geqslant 0$. In particular, *M* is invertible.

#### Proof

First, after step 0 (that is, before we do anything), the lemma is clear.

Suppose after step $i-1$, we get a matrix $M'=(m'_{jk})=\textrm{Id}+N'\cdot t$, where each entry of $N'$ has *t*-order at least 0. Consider step *i*. Let $j>i$. Step *i* turns each entry $m'_{jk}$ into

$$\begin{aligned}m''_{jk}=m'_{jk}-m'_{ji}(m'_{ii})^{-1}m'_{ik}.\end{aligned}$$

Note that ${{\,\textrm{ord}\,}}_t (m'_{ji}(m'_{ii})^{-1}m'_{ik})\geqslant 1$. If $j\ne k$ then ${{\,\textrm{ord}\,}}_t m'_{jk}\geqslant 1$ and thus ${{\,\textrm{ord}\,}}_t m''_{jk}\geqslant 1$. If $j=k$ then $m'_{jk}=1+n'_{jk}\cdot t$ for some $n'_{jk}\in D(\!(t)\!)$ with ${{\,\textrm{ord}\,}}_t n'_{jk}\geqslant 0$, and thus $m''_{jk}=1+n''_{jk}\cdot t$ for some $n''_{jk}\in D(\!(t)\!)$ with ${{\,\textrm{ord}\,}}_t n''_{jk}\geqslant 0$. $\square$

### Behavior of the degree function under representations of skew fields

Let *D* be a skew field and let *t* be a central variable. Let $n\in \mathbb {N}^+$ and suppose that there is a ring homomorphism $\sigma :D\rightarrow M_n(D)$. (Recall that ring homomorphisms are unital, i.e., they send the identity to the identity.) We extend $\sigma$ to a ring homomorphism (still denoted by) $\sigma :D(\!( t )\!) \rightarrow M_n(D(\!( t )\!))$ by

$$\begin{aligned} \sigma \left( \sum ^{\infty }_{i=k} d_it^i \right) =\sum ^{\infty }_{i=k}\sigma (d_i)\cdot t^i. \end{aligned}$$

The goal of this subsection is:

#### Lemma 2.14

For every $k\in \mathbb {N}^+$ and $A \in M_k(D [t^\pm ])$ we have

$$\begin{aligned}\deg _t{{\,\mathrm{\det }\,}}_{D(t)}\sigma (A)=n\cdot \deg _t{{\,\mathrm{\det }\,}}_{D(t)}A,\end{aligned}$$

where $\sigma (A)$ is the matrix obtained by applying $\sigma$ to every entry of *A*.

Before the proof of Lemma 2.14, we state a useful corollary.

#### Corollary 2.15

The representation $\sigma$ extends to a ring homomorphism $D(t)\rightarrow M_n(D(t))$.

#### Proof

By Lemma 2.14 applied with $k=1$, the matrix $\sigma (p)$ is invertible in $M_n(D(t))$ for every non-zero $p\in D[t]$. By the universal property of the Ore localization, $\sigma$ extends to a ring homomorphism

$$\begin{aligned}D(t)={{\,\textrm{Ore}\,}}(D[t])\rightarrow {{\,\textrm{Ore}\,}}(M_n(D[t]),T)=M_n(D(t)),\end{aligned}$$

where $T\subset M_n(D[t])$ is the set of non-zero divisors. Here, the last equality follows from the universal property of the Ore localization and the following two observations about the natural inclusion $M_n(D[t])\hookrightarrow M_n(D(t))$:

(i) Every matrix $A\in T$ is invertible in $M_n(D(t))$. Indeed, suppose *A* is not invertible over $M_n(D(t))$. Then there is a non-zero matrix $B\in M_n(D(t))$ such that $B\cdot A=0$. Using the Ore condition, one can find a non-zero element $p\in D[t]$ such that $p\cdot B$ is a matrix over *D*[*t*]. But then *A* is a non-trivial zero divisor as $p\cdot B\cdot A=0$, a contradiction.
(ii) Every ring homomorphism $\phi :M_n(D[t])\rightarrow R$ that maps every element of *T* to an invertible element uniquely extends to a ring homomorphism $M_n(D(t))\rightarrow R$. Indeed, for every matrix $A\in M_n(D(t))$, there exists a non-zero element $p\in D[t]$ such that $p\cdot A$ is a matrix over *D*[*t*]. Note that $p\cdot \textrm{Id}\in T$ and thus $\phi (p\cdot \textrm{Id})$ is invertible in *R*. So if $\phi$ can be extended to $M_n(D(t))$, it has to map *A* to $\phi (p\cdot \textrm{Id})^{-1}\cdot \phi (p\cdot A)$, which shows uniqueness. To extend $\phi$, define $\phi (A)=\phi (p\cdot \textrm{Id})^{-1}\cdot \phi (p\cdot A)$. We have to check that this is well defined. Assume that there is another non-zero element $q\in D[t]$ such that $q\cdot A\in M_n(D[t])$. Then by the Ore condition there exist $r,s\in D[t], s\ne 0$ such that $rp=sq$. In particular we have $rp\ne 0$ and also $r\ne 0$. We have
  $$\begin{aligned} \phi (p\cdot \textrm{Id})^{-1}\cdot \phi (p\cdot A)&=\phi (p\cdot \textrm{Id})^{-1}\cdot \phi (r\cdot \textrm{Id})^{-1}\cdot \phi (rp\cdot A)\\&=\phi (rp\cdot \textrm{Id})^{-1}\cdot \phi (rp\cdot A)\\&=\phi (sq\cdot \textrm{Id})^{-1}\cdot \phi (sq\cdot A)\\&=\phi (q\cdot \textrm{Id})^{-1}\cdot \phi (s\cdot \textrm{Id})^{-1}\cdot \phi (sq\cdot A)\\&=\phi (q\cdot \textrm{Id})^{-1}\cdot \phi (q\cdot A). \end{aligned}$$

$$\square $$

The lemma below is the first step towards proving Lemma 2.14.

#### Lemma 2.16

For all $$z\in D(\!( t )\!)$$,

$$\begin{aligned} {{\,\textrm{ord}\,}}_t {{\,\mathrm{\det }\,}}_{D(\!( t )\!)} \sigma (z) = n\cdot {{\,\textrm{ord}\,}}_t z. \end{aligned}$$

#### Proof

The lemma is trivial if $z=0$. So let $z=\sum ^{\infty }_{i=\ell }d_i t^i\in D(\!( t )\!)\smallsetminus \{0\}$, where $d_i\in D$ and $d_{\ell }\ne 0$. Then

$$\begin{aligned} z=d_{\ell }\cdot t^{\ell }\cdot \left( \sum ^{\infty }_{i=\ell }d^{-1}_{\ell } d_i t^{i-\ell }\right) . \end{aligned}$$

Thus

$$\begin{aligned} {{\,\textrm{ord}\,}}_t{{\,\mathrm{\det }\,}}_{D(\!( t )\!)}\sigma (z)= & {} {{\,\textrm{ord}\,}}_t{{\,\mathrm{\det }\,}}_{D(\!( t )\!)}\sigma (d_{\ell })+{{\,\textrm{ord}\,}}_t{{\,\mathrm{\det }\,}}_{D(\!( t )\!)}\sigma (t^{\ell })\\{} & {} + {{\,\textrm{ord}\,}}_t{{\,\mathrm{\det }\,}}_{D(\!( t )\!)}\sigma \left( \sum ^{\infty }_{i=\ell }d^{-1}_{\ell } d_i t^{i-\ell }\right) . \end{aligned}$$

Since $d_{\ell }\ne 0$, $\sigma (d_{\ell })$ is an invertible matrix over *D*. Thus,

$$\begin{aligned} {{\,\textrm{ord}\,}}_t{{\,\mathrm{\det }\,}}_{D(\!( t )\!)}(\sigma (d_{\ell }))=0. \end{aligned}$$

By Lemma 2.13, we have

$$\begin{aligned} {{\,\textrm{ord}\,}}_{t}{{\,\mathrm{\det }\,}}_{D(\!( t )\!)}\sigma \left( \sum ^{\infty }_{i=\ell }d^{-1}_{\ell } d_i t^{i-\ell }\right) =0. \end{aligned}$$

Note also that

$$\begin{aligned} {{\,\textrm{ord}\,}}_{t}{{\,\mathrm{\det }\,}}_{D(\!( t )\!)}(\textrm{Id}_n\cdot t^{\ell })=n\ell . \end{aligned}$$

We thus have ${{\,\textrm{ord}\,}}_t{{\,\mathrm{\det }\,}}_{D(\!( t )\!)}\sigma (z)=n\ell =n\cdot {{\,\textrm{ord}\,}}_t z$, as desired. $\square$

#### Proof of Lemma 2.14

First consider $z\in D(t)$. Let $\alpha :D(t)\rightarrow D(\!( t )\!)$ be the embedding given in Sect. 2.4 and let $z'={{\,\mathrm{\det }\,}}^c_{D(t)}\sigma (z)$ be the canonical representative of the Dieudonné determinant. Then

$$\begin{aligned} {{\,\textrm{ord}\,}}_t{{\,\mathrm{\det }\,}}_{D(t)}\sigma (z)={{\,\textrm{ord}\,}}_t z'={{\,\textrm{ord}\,}}_t \alpha (z')={{\,\textrm{ord}\,}}_t{{\,\mathrm{\det }\,}}_{D(\!( t )\!)}\sigma (z)=n\cdot {{\,\textrm{ord}\,}}_t z, \end{aligned}$$

where the last equality follows from Lemma 2.16.

Let $\beta :D(t) \rightarrow D(t)$ be the ring automorphism constructed in Sect. 2.4. We also view $\beta$ as an automorphism of the semi-group $(D(t))^{\times }_{\textrm{ab}}\sqcup \{0\}$. We have

$$\begin{aligned} {{\,\textrm{ord}\,}}_t\beta ({{\,\mathrm{\det }\,}}_{D(t)}\sigma (z))={{\,\textrm{ord}\,}}_t{{\,\mathrm{\det }\,}}_{D(t)}\beta (\sigma (z)) ={{\,\textrm{ord}\,}}_t{{\,\mathrm{\det }\,}}_{D(t)}\sigma (\beta (z))=n\cdot {{\,\textrm{ord}\,}}_t\beta (z), \end{aligned}$$

where the third equality follows from Lemma 2.16.

Equation (1) then implies

2 $$\begin{aligned} \deg _t{{\,\mathrm{\det }\,}}_{D(t)}\sigma (z)=n\cdot \deg _t z. \end{aligned}$$

Now consider the matrix *A*. By elementary row operations over *D*(*t*) we can turn *A* into an upper-triangular matrix over *D*(*t*). In more details, there are elementary matrices $U_1,\dots , U_\kappa \in M_k\big (D(t)\big )$ whose diagonal entries are all $\pm 1$ such that $B=(\prod ^\kappa _{i=1}U_i)A\in D(t)$ is an upper-triangular matrix. So

$$\begin{aligned} \deg _t{{\,\mathrm{\det }\,}}_{D(t)} B=\deg _t(\pm {{\,\mathrm{\det }\,}}_{D(t)}A)=\deg _t{{\,\mathrm{\det }\,}}_{D(t)} A. \end{aligned}$$

The matrix $\sigma (B)$ is a block-wise upper-triangular matrix. Note that ${{\,\mathrm{\det }\,}}_{D(t)}\sigma (U_i)=\pm 1$ for all *i*. Thus

$$\begin{aligned}{{\,\mathrm{\det }\,}}_{D(t)}\sigma (B)=\left( \prod ^k_{i=1}{{\,\mathrm{\det }\,}}_{D(t)}\sigma (U_i)\right) \cdot {{\,\mathrm{\det }\,}}_{D(t)}\sigma (A)=\pm {{\,\mathrm{\det }\,}}_{D(t)}\sigma (A).\end{aligned}$$

As *B* is upper-triangular, ${{\,\mathrm{\det }\,}}^c_{D(t)} B$ is the image of the product of its diagonal entries in $(D(t))^{\times }_{\textrm{ab}}$. As $\sigma (B)$ is block-wise upper-triangular, ${{\,\mathrm{\det }\,}}_{D(t)}\sigma (B)$ is the product of the Dieudonné determinants of the diagonal blocks. Combining these with equation (2), we see that

$$\begin{aligned} \deg _t{{\,\mathrm{\det }\,}}_{D(t)}\sigma (B)=n\cdot \deg _t{{\,\mathrm{\det }\,}}_{D(t)}B=n\cdot \deg _t{{\,\mathrm{\det }\,}}_{D(t)} A. \end{aligned}$$

$$\square $$

### Locally indicable groups and Linnell skew fields

A group *G* is *locally indicable* if every non-trivial finitely generated subgroup of *G* admits an epimorphism onto $\mathbb {Z}$.

#### Example 2.17

All free groups are locally indicable. More generally, except for the fundamental group of the projective plane $P^2$, all surface groups are locally indicable. Indeed, suppose $S\ne P^2$ is a surface. If *S* is not closed, then $\pi _1(S)$ is free and thus is locally indicable. If $S=S^2$ is the 2-sphere, then $\pi _1(S)$ is of course locally indicable. In all other cases $b_1(S)\ne 0$ and thus $\pi _1(S)$ has a surjection onto $\mathbb {Z}$. The kernel *K* of this surjection corresponds to an infinite cyclic cover of *S* and thus is a free group. So $\pi _1(S)$ is a semi-direct product $\pi _1(S)=K\rtimes \mathbb {Z}$ with *K* free, and thus is locally indicable.

The following lemmata are well known to experts. We include proofs for completeness.

#### Lemma 2.18

Suppose that *N* and *Q* are locally indicable groups and a group *G* fits into a short exact sequence

$$\begin{aligned}1\rightarrow N \rightarrow G \rightarrow Q\rightarrow 1.\end{aligned}$$

Then *G* is locally indicable.

#### Proof

Let *H* be a non-trivial finitely generated subgroup of *G*. Let $\phi :G\rightarrow Q$ be the quotient map with $\ker \phi =N$. Then $\phi (H)$ is a finitely generated subgroup of *Q*. If $\phi (H)\ne \{1\}$, then, as *Q* is locally indicable, $\phi (H)$ has a surjection onto $\mathbb {Z}$ and thus so does *H*. If $\phi (H)=\{1\}$ then $H\leqslant N$ and, as *N* is locally indicable, *H* has a surjection onto $\mathbb {Z}$. $\square$

#### Lemma 2.19

Free products of locally indicable groups are locally indicable.

#### Proof

Let $G={*}_{i\in I}G_i$ be a free product of locally indicable groups $G_i$ and let $\{1\}< H\leqslant G$ be a finitely generated subgroup of *G*. For each *i*, let $\alpha _i:G\rightarrow G_i$ be the natural homomorphism. If $\alpha _i(H)\ne \{1\}$ for some *i*, then *H* has a surjection onto $\mathbb {Z}$ as $G_i$ is locally indicable. So it suffices to prove that the subgroup $\bigcap _{i\in I}\ker (\alpha _i)$ is locally indicable. By the Kurosh subgroup theorem, $\bigcap _{i\in I}\ker (\alpha _i)$ is a free product:

$$\begin{aligned}\bigcap _{i\in I}\ker (\alpha _i)=F*({*}_{j\in J}K_j),\end{aligned}$$

where *F* is a free group and each $K_j$ is a conjugate of a subgroup of some $G_i$. As $\bigcap _{i\in I}\ker (\alpha _i)$ is normal in *G*, each $K_j$ is isomorphic to a subgroup of the intersection $\bigcap _{i\in I}\ker (\alpha _i)\cap G_{i'}$ for some $i'\in I$. As $\bigcap _{i\in I}\ker (\alpha _i)\cap G_{i'}=\{1\}$ for all $i'\in I$, $K_j=\{1\}$ for all *j* and $\bigcap _{i\in I}\ker (\alpha _i)=F$ is a free group, which is locally indicable. $\square$

To obtain more examples of locally indicable groups we briefly recall some notions from the theory of 3-manifolds. For details the reader is referred to the book [1]. Let *M* be a compact connected orientable irreducible 3-manifold with empty or toroidal boundary. *M* is called *non-positively curved* if there is a Riemannian metric on the interior of *M* with non-positive sectional curvature. *M* is called *geometric* if *M* supports one of the geometries $S^3,S^2\times \mathbb {R}^1,\mathbb {R}^3,\textrm{NIL},\textrm{SOL},\widetilde{\textrm{SL}_2(\mathbb {R})},\mathbb {H}^3$, $\mathbb {H}^2\times \mathbb {R}$.

#### Proposition 2.20

Suppose that *M* is a compact connected orientable 3-manifold with empty or toroidal boundary. Then $\pi _1(M)$ is virtually locally indicable, i.e., $\pi _1(M)$ has a finite-index locally indicable subgroup.

#### Proof

First consider the case where *M* is prime. If *M* is not irreducible, then $M=S^1\times S^2$ and $\pi _1(M)=\mathbb {Z}$ is locally indicable. So let us assume that *M* is irreducible. If *M* is not a closed graph manifold, then *M* is non-positively curved. Indeed, the non-positive curvature of *M* follows from the resolution of the Virtually Haken Conjecture [2], Thurston’s Hyperbolization Theorem [47] and work of Leeb [32]. Work of Agol, Duchamp, Gruenberg, Haglund, Kahn, Krob, Marković, Liu, Perelman, Przytycki, Rhemtulla, and Wise (see [1, (G.30)] for an explanation) imply that if *M* is non-positively curved then $\pi _1(M)$ is virtually bi-orderable and thus is virtually locally indicable [33].

If *M* is a non-geometric closed graph manifold, then by [30, Lemma 2.1] *M* has a finite-sheeted cover *N* whose Seifert pieces are products of circles with orientable surfaces of genus at least two. We claim that $b_1(N)>0$. Indeed, $\pi _1(N)$ acts on a finite graph (*V*, *E*) with vertex stabilizers the fundamental group of its Seifert pieces and edge stabilizers isomorphic to $\mathbb {Z}^2$. By [6, Theorem 2], there is a long exact sequence

$$\begin{aligned}\cdots \rightarrow \bigoplus _{e\in E}H_1(\mathrm {Stab(e)},\mathbb {Q})\rightarrow \bigoplus _{v\in V}H_1(\textrm{Stab}(v),\mathbb {Q})\rightarrow H_1(\pi _1(N),\mathbb {Q})\rightarrow \cdots \end{aligned}$$

For each $v\in V$ and each edge *e* incident to *v*, $\textrm{Stab}(v)$ is the fundamental group of a Seifert piece $S^1\times \Sigma$, where $\Sigma$ is a surface of genus at least two, and *e* corresponds to a boundary component of $\Sigma$ (if *e* is a loop then it corresponds to two boundary components). Therefore, $b_1(S^1\times \Sigma )\geqslant \deg (v)+4$. So

$$\begin{aligned} b_1(N)=&\dim _{\mathbb {Q}} H_1(\pi _1(N),\mathbb {Q})\\ \geqslant&\sum _{v\in V}\dim _{\mathbb {Q}}H_1(\textrm{Stab}(v),\mathbb {Q})-\sum _{e\in E}\dim _{\mathbb {Q}}H_1(\textrm{Stab}(e),\mathbb {Q})\\ \geqslant&\sum _{v\in V}\deg (v)+4|V|-2|E|\\ =&4|V|>0. \end{aligned}$$

We claim that $\pi _1(N)$ is locally indicable. Let $H\leqslant \pi _1(N)$ be a finitely generated non-trivial subgroup. If *H* is of finite index, then $b_1(H)\geqslant b_1(N)>0$. If *H* is of infinite index, then the proof of [21, Theorem 6.1] (see also [22, Lemma 2]) shows that $b_1(H)\geqslant 1$. So in any case *H* has a surjection onto $\mathbb {Z}$.

Now suppose that *M* is a closed geometric graph manifold. Then *M* cannot support the $\mathbb {H}^3$ geometry. If *M* supports one of the geometries $S^3,S^2\times \mathbb {R}^1,\mathbb {R}^3,\textrm{NIL},\textrm{SOL}$, then by [18, Lemmata 8.1, 9.2, 10.1, 11.1], $\pi _1(M)$ is either virtually free abelian or virtually $\mathbb {Z}\rtimes \mathbb {Z}$, and thus is virtually locally indicable.

If *M* supports the $\widetilde{\textrm{SL}_2(\mathbb {R})}$ geometry then $\pi _1(M)$ is a semi-direct product $\pi _1(M)=\mathbb {Z}\rtimes F$ for some non-cyclic free group *F* and thus is locally indicable.

If *M* supports the $\mathbb {H}^2\times \mathbb {R}$ geometry then $\pi _1(M)$ is virtually a product $\mathbb {Z}\times F$ where *F* is a non-cyclic free group, and thus is virtually locally indicable. This finishes the proof for the case where *M* is prime.

In the general case, the prime decomposition theorem implies that $\pi _1(M)$ is a free product:

$$\begin{aligned}\pi _1(M)={*}^n_{i=1}\pi _1(M_i),\end{aligned}$$

where $M_i$ are prime 3-manifolds. By the previous part of the proof, for each *i* there is a finite-index locally indicable normal subgroup $H_i$ of $\pi _1 (M_i)$. Let *H* be the kernel of the natural homomorphism

$$\begin{aligned}\pi _1(M)\rightarrow \bigoplus _{i=1}^n(\pi _1(M_i)/H_i).\end{aligned}$$

Then *H* is a finite-index normal subgroup of $\pi _1(M)$. By the Kurosh subgroup theorem, *H* is a free product:

$$\begin{aligned}H=F*({*}_{j\in J}K_j),\end{aligned}$$

where *F* is a free group and each $K_j$ is a conjugate of a subgroup of some $\pi _1(M_i)$. As *H* is normal, each $K_j$ is isomorphic to a subgroup of $H\cap \pi _1(M_i)=H_i$ for some *i*, which is locally indicable. By Lemma 2.19, *H* is locally indicable, being a free product of locally indicable groups. $\square$

Let *G* be a locally indicable group. By [26, Theorem 1.1], *G* satisfies the Atiyah conjecture over $\mathbb {C}$. Let $\mathcal {D}_G$ be the division closure of $\mathbb {C}G$ in $\mathcal {U}_G$, the algebra of affiliated operators of the group von Neumann algebra of *G*. Since *G* is obviously torsion-free, $\mathcal {D}_ G$ is a skew field [38, Lemma 10.39] and is called the *Linnell skew field* of $\mathbb {C}G$. See also [35].

### Hughes-free skew fields

The notion of Hughes-freeness was introduced by Hughes [23] in order to prove isomorphism between certain skew fields.

#### Definition 2.21

Let *R* be a ring. An *R-field* consists of a skew field *D* and a ring homomorphism $\beta :R\rightarrow D$. The skew field *D* is called an *epic* *R-field* if *D* is the skew field generated by $\beta (R)$.

#### Definition 2.22

Let *DG* be a twisted group ring with *D* a skew field and *G* a locally indicable group. An epic *DG*-field $\beta :DG\rightarrow E$ is *Hughes-free* if for every non-trivial finitely generated subgroup *H* of *G*, every normal subgroup *N* of *H* with $H/N\cong \mathbb {Z}$, and every $h_1,\cdots , h_n\in H$ in distinct cosets of *N* in *H*, the sum $E_N\beta (h_1)+\cdots +E_N\beta (h_n)$ is direct, where $E_N$ is the division closure of $\beta (DN)$ in *E*, and *DN* is the subring of *DG* generated by *D* and *N*.

#### Example 2.23

Let *G* be a locally indicable group. Then its Linnell skew field is a Hughes-free $\mathbb {C}G$-field [26, Corollary 6.2].

### Specialization, universality and Lewin groups

Let *R* be a ring.

#### Definition 2.24

Given two epic *R*-fields $\beta :R\rightarrow D$ and $\beta ':R\rightarrow D'$, a *specialization* of *D* to $D'$ with respect to *R* is a pair $(S,\alpha )$ where *S* is a subring of *D* containing ${{\,\textrm{im}\,}}\beta$, the map $\alpha :S\rightarrow D'$ is a ring homomorphism with $\alpha \circ \beta =\beta '$, and every element in *S* not mapped to 0 by $\alpha$ is invertible in *S*.

#### Definition 2.25

An epic *R*-field $\beta :R\rightarrow D$ is called the *universal* *R-field* if for every epic *R*-field $D'$ there is a specialization of *D* to $D'$ with respect to *R*. If in addition the map $R\rightarrow D$ is injective, then *D* is called *the universal field of fractions* of *R*.

#### Definition 2.26

A group *G* is *Lewin* if for every twisted group ring *DG* with *D* a skew field, there is a Hughes-free universal *DG*-field.

Let *G* be a finitely generated Lewin group. By [25, Proposition 4.1], *G* is locally indicable and thus there is a natural embedding $\tau :\mathbb {C}G \rightarrow \mathcal {D}_G$ of $\mathbb {C}G$ into its Linnell skew field $\mathcal {D}_G$.

Let $q:G\twoheadrightarrow G_{\textrm{fab}}$ be the natural quotient homomorphism of *G* onto its maximal free abelian quotient $G_{\textrm{fab}}$. Let *X* be a basis of $G_{\textrm{fab}}$ and let $x\in X$. Consider the group ring $\mathcal {D}_G G_{\textrm{fab}}$. Since we are interested in computing $\deg _x$, the difference between the highest and the lowest power of *x* with respect to the basis *X*, we will denote ${{\,\textrm{Ore}\,}}(\mathcal {D}_G G_{\textrm{fab}})$ by $\mathcal {D}_G(X)$ to emphasize the role played by *X*.

Let $\sigma :G\rightarrow {{\,\textrm{GL}\,}}_n(\mathbb {C})$ be a complex representation of *G* of finite dimension *n*. For reasons that will be clear later, we are interested in the representations

$$\begin{aligned} \sigma \otimes _{\mathbb {Z}} q :&G\rightarrow {{\,\textrm{GL}\,}}_n(\mathbb {C}(X)),~~ (\sigma \otimes _{\mathbb {Z}} q)(g)=\sigma (g)q(g),\\ \sigma \otimes _{\mathbb {C}}\tau \otimes _{\mathbb {Z}} q :&G\rightarrow {{\,\textrm{GL}\,}}_n(\mathcal {D}_G(X)),~~(\sigma \otimes _{\mathbb {C}}\tau \otimes _{\mathbb {Z}} q)(g)=\sigma (g)\tau (g)q(g). \end{aligned}$$

Let $M\in M_m(\mathbb {Z}G)$ be a square matrix over $\mathbb {Z}G$. By applying $\sigma \otimes _{\mathbb {Z}} q$ and ${\sigma \otimes _{\mathbb {C}}\tau \otimes _{\mathbb {Z}} q}$ to every entry of *M* we obtain matrices $(\sigma \otimes _{\mathbb {Z}} q)(M)\in M_{mn}(\mathbb {C}(X))$ and $(\sigma \otimes _{\mathbb {C}}\tau \otimes _{\mathbb {Z}} q)(M)\in M_{mn}(\mathcal {D}_G(X))$, respectively.

#### Lemma 2.27

We have the inequality

$$\begin{aligned}\deg _x{{\,\mathrm{\det }\,}}_{\mathbb {C}(X)}(\sigma \otimes _{\mathbb {Z}}q)(M)\leqslant \deg _x{{\,\mathrm{\det }\,}}_{\mathcal {D}_G(X)} (\sigma \otimes _{\mathbb {C}}\tau \otimes _{\mathbb {Z}} q) (M).\end{aligned}$$

#### Proof

We would like to apply [13, Proposition 4.1]. Let $Y=X\smallsetminus \{x\}$. Consider the ring $R=\mathbb {C}G[Y^{\pm }]$. The ring $\mathbb {C}G[X^{\pm }]=R[x^{\pm }]$ is the ring of Laurent polynomials over *R*. Consider two *R*-fields

$$\begin{aligned} \beta :&R\rightarrow \mathcal {D}_G(Y),~~\beta (g)=\tau (g),\beta (y)=y \text { for all }g\in G,y\in Y,\\ \beta ' :&R\rightarrow \mathbb {C}(Y),~~\beta '(g)=1, \beta '(y)=y \text { for all }g\in G,y\in Y. \end{aligned}$$

Consider the representation

$$\begin{aligned}\sigma \otimes _{\mathbb {C}}\textrm{id}_{\mathbb {C}G}\otimes _{\mathbb {Z}}q:G \rightarrow {{\,\textrm{GL}\,}}_n(R[x^{\pm }]).\end{aligned}$$

As above, by applying $\sigma \otimes _{\mathbb {C}}\textrm{id}_{\mathbb {C}G}\otimes _{\mathbb {Z}}q$ to every entry of *M* we get a matrix $(\sigma \otimes _{\mathbb {C}}\textrm{id}_{\mathbb {C}G}\otimes _{\mathbb {Z}}q)(M)$. Note that $\beta '$ can be extended to a map from $R[x^{\pm }]$ to $\mathbb {C}(X)$ by setting $\beta '(x)=x$. With this convention we can then apply $\beta '$ to each entry of the matrix $(\sigma \otimes _{\mathbb {C}}\textrm{id}_{\mathbb {C}G}\otimes _{\mathbb {Z}}q)(M)$ to get a square matrix $\beta '((\sigma \otimes _{\mathbb {C}}\textrm{id}_{\mathbb {C}G}\otimes _{\mathbb {Z}}q)(M))$ over $\mathbb {C}(X)$. Note that

$$\begin{aligned}(\sigma \otimes _{\mathbb {Z}}q)(M)=\beta '((\sigma \otimes _{\mathbb {C}}\textrm{id}_{\mathbb {C}G}\otimes _{\mathbb {Z}}q)(M)).\end{aligned}$$

Similarly, we can extend $\beta$ to a map from $R[x^{\pm }]$ to $\mathcal {D}_G(X)$ by setting $\beta (x)=x$. We have

$$\begin{aligned}(\sigma \otimes _{\mathbb {C}}\tau \otimes _{\mathbb {Z}} q) (M)=\beta ((\sigma \otimes _{\mathbb {C}}\textrm{id}_{\mathbb {C}G}\otimes _{\mathbb {Z}}q)(M)).\end{aligned}$$

By [25, Theorem 3.7], $\mathcal {D}_G$ is the universal field of fractions of $\mathbb {C}G$. Therefore, there exists a specialization of $\mathcal {D}_G(Y)$ to $\mathbb {C}(Y)$ with respect to *R*. The desired result thus follows from [13, Proposition 4.1]. $\square$

### Rational semirings

In this and the next two subsections we recall results about rational semirings that are necessary in the study of twisted $\ell ^2$-Betti numbers. By a *semiring* *R* we mean a set together with an associative commutative addition and an associative multiplication with identity element $1_R$ which is distributive over the addition. Let *U* be a group and let *R* be a semiring. We say that *R* is a *rational* *U-semiring* if

(i) There is a map $\diamond :R\rightarrow R, r\mapsto r^{\diamond }$, called the *rational structure* on *R*.
(ii) *R* is a *U*-*biset*, i.e., *U* acts on both sides of *R* in a compatible way: $(ur)v=u(rv)$ for all $u,v\in U,r\in R$.
(iii) For every $u,v\in U$ and $r\in R$, $(urv)^{\diamond }=v^{-1}r^{\diamond }u^{-1}$.

#### Example 2.28

Let *G* be a group and let *R* be a ring with a ring homomorphism $\sigma :\mathbb {C}G\rightarrow R$. Then *R* is a $\mathbb {C}^{\times } G$-biset with the action given by

$$\begin{aligned}(c_1g_1,c_2g_2,r)\mapsto \sigma (c_1g_1)\cdot r\cdot \sigma (c_2g_2)\end{aligned}$$

for all $c_1,c_2\in \mathbb {C}^{\times },g_1,g_2\in G,r\in R$. Let *S* be the division closure of $\sigma (\mathbb {C}G)$ in *R*. Then *S* is a rational $\mathbb {C}^{\times }G$-semiring under the rational map given by the following: if $s\in S$ is invertible in *R* then $s^{\diamond }=s^{-1}$; otherwise, $s^{\diamond }=0$.

A *morphism of rational* *U-semirings* $\Phi :R_1\rightarrow R_2$ is a map such that

(i) $$\Phi (r+r')=\Phi (r)+\Phi (r')$$;
(ii) $$\Phi (rr')=\Phi (r)\Phi (r')$$ and $$\Phi (1_{R_1})=1_{R_2}$$;
(iii) $\Phi (r^{\diamond })=\Phi (r)^{\diamond }$ for all $r\in R_1$;
(iv) $\Phi (urv)=u\Phi (r)v$ for all $u,v\in U,r\in R_1$.

Below, we recall the construction of the *universal rational* *U-semiring* ${{\,\textrm{Rat}\,}}(U)$. It is characterized by the following universal property:

#### Lemma 2.29

[8, Lemma 4.7] If *R* is a rational *U*-semiring, then there exists a unique morphism of rational *U*-semirings $\Phi :{{\,\textrm{Rat}\,}}(U)\rightarrow R$.

Before defining ${{\,\textrm{Rat}\,}}(U)$, we present some definitions and notation:

- If *X* is a set, then the free additive semigroup on *X* is $\mathbb {N}X\smallsetminus \{0\}$. Here, our convention is $0\in \mathbb {N}$. Note that if *X* is a multiplicative monoid with a *U*-biset structure, then $\mathbb {N}X\smallsetminus \{0\}$ is naturally a *U*-semiring.
- If *X* is a *U*-biset, then $X^{\times ^n_U}$ is the set of equivalence classes of words in *X* of length *n* with respect to the relation generated by
  $$\begin{aligned}x_1x_2\cdots (x_iu)x_{i+1}\cdots x_n\sim x_1x_2\cdots x_i(ux_{i+1})\cdots x_n\end{aligned}$$
  for all $x_1,x_2,\cdots ,x_n\in X, u\in U$. The multiplicative free monoid on *X* over *U* is defined as
  $$\begin{aligned}U\natural X=\bigcup ^{\infty }_{n=0}X^{\times ^n_U}\end{aligned}$$
  where by definition $X^{\times ^0_U}=U$. The monoid structure is defined as follows: for $x,y\in U\natural X$, if $x,y\in U$, then $xy\in U$ is just the product under the group operation of *U*; if $x\in U$ and $y\in (U\natural X)\smallsetminus U$, then *xy* is given by the left action of *U* on $(U\natural X)\smallsetminus U$; if $x\in (U\natural X)\smallsetminus U$ and $y\in U$, then *xy* is given by the right action of *U* on $(U\natural X)\smallsetminus U$; finally, if $x,y\in (U\natural X)\smallsetminus U$, then *xy* is the concatenation of *x* and *y*. Observe that $\mathbb {N}[U\natural X]\smallsetminus \{0\}$ is naturally a *U*-semiring.
- If *X* is a *U*-biset, then $X^{\diamond }$ denotes a disjoint copy of *X* together with a bijective map $X\rightarrow X^{\diamond },x\mapsto x^{\diamond }$, and a *U*-biset structure given by
  $$\begin{aligned}ux^{\diamond }v=(v^{-1}xu^{-1})^{\diamond }\end{aligned}$$
  for all $$u,v\in U,x\in X$$.

The *universal rational* *U-semiring* is defined as follows:

- First consider the *U*-semiring $\mathbb {N}U\smallsetminus \{0\}$ and set $X_0=\emptyset ,X_1=(\mathbb {N}U\smallsetminus \{0\})^{\diamond }$.
- Suppose $n\geqslant 1$, $X_n$ is a *U*-biset and $X_{n-1}$ is a *U*-sub-biset of $X_n$. Consider the *U*-semiring $\mathbb {N}[U\natural X_n]\smallsetminus \{0\}$ and the *U*-sub-biset $\mathbb {N}[U\natural X_n]\smallsetminus \mathbb {N}[U\natural X_{n-1}]$. Define
  $$\begin{aligned} X_{n+1}=(\mathbb {N}[U\natural X_n]\smallsetminus \mathbb {N}[U\natural X_{n-1}])^{\diamond }\cup X_n. \end{aligned}$$
- Then $X=\bigcup _{n\geqslant 0} X_n$ is a *U*-biset. Let
  $$\begin{aligned} {{\,\textrm{Rat}\,}}(U)=\mathbb {N}[U\natural X]\smallsetminus \{0\}. \end{aligned}$$

For later reference, we note the following.

#### Theorem 2.30

([8, Lemma 5.4 and Theorem 5.7]) If $\alpha \in {{\,\textrm{Rat}\,}}(U)$, then there exists a subgroup $\textrm{source}(\alpha )\leqslant U$ with the following properties.

(i) $\textrm{source}(\alpha )$ is finitely generated and $\alpha \in {{\,\textrm{Rat}\,}}(\textrm{source}(\alpha ))\cdot U$.
(ii) If *V* is a subgroup of *U* such that $\alpha \in {{\,\textrm{Rat}\,}}(V)\cdot U$, then $\textrm{source}(\alpha )\leqslant V$.

An element $\alpha \in {{\,\textrm{Rat}\,}}(U)$ is called *primitive* if $\alpha \in {{\,\textrm{Rat}\,}}(\textrm{source}(\alpha ))$.

### Trees and complexity

Let $\mathcal {T}$ be the set of all finite rooted trees up to isomorphism. Here we recall that $\mathcal {T}$ has a well-order satisfying certain properties and is a *U*-semiring for any group *U*. The order will be used to define a complexity on elements of ${{\,\textrm{Rat}\,}}(U)$.

Denote by $0_{\mathcal {T}}$ the one-vertex tree. If $0_{\mathcal {T}}\ne X\in \mathcal {T}$, denote by $\textrm{fam}(X)$ the finite family of finite rooted trees obtained from *X* by deleting the root and all incident edges, where the root of an element $Y\in \textrm{fam}(X)$ is the unique vertex of *Y* that is incident to the root of *X*. We denote by $\exp (X)$ the tree obtained from *X* by adding a new vertex which is declared to be the root of $\exp (X)$, and a new edge joining it to the root of *X*.

Let $X,Y\in \mathcal {T}$. The sum $X+Y\in \mathcal {T}$ is the rooted tree obtained by identifying the roots of *X*, *Y* and declaring it to be the root of $X+Y$. The product $X\cdot Y$ is defined as follows: if one of *X*, *Y* is $0_{\mathcal {T}}$, then $X\cdot Y=0_{\mathcal {T}}$ by definition; if $X,Y\ne 0_{\mathcal {T}}$, the product $X\cdot Y$ is obtained by adding pairwise elements of $\textrm{fam}(X)$ with elements of $\textrm{fam}(Y)$, and then connecting all the resulting finite rooted trees by adding a new vertex with incident edges to their roots, and declaring the new vertex to be the root of $X\cdot Y$, i.e.,

$$\begin{aligned}X\cdot Y=\sum _{\begin{array}{c} X'\in \textrm{fam}(X)\\ Y'\in \textrm{fam}(Y) \end{array}}\exp (X'+Y').\end{aligned}$$

The rational map of $\mathcal {T}$ is given by

$$\begin{aligned}X^{\diamond }=\exp ^2(X).\end{aligned}$$

The group *U* acts on both sides of $\mathcal {T}$ by the trivial action. With these operations, $\mathcal {T}$ is a rational *U*-semiring.

Let $\mathcal {T}_n\subset \mathcal {T}$ be the subset consisting of all elements with at most *n* edges. The following defines a well-order on $\mathcal {T}$ [8, Lemma 3.3]:

- $0_{\mathcal {T}}$ is the least element of $\mathcal {T}$.
- Suppose that $\mathcal {T}_{n-1}$ has already been ordered for some $n\geqslant 1$. Let $X,Y\in \mathcal {T}_n\smallsetminus \{0_{\mathcal {T}}\}$. Let $\log (X)$ be the largest element of $\mathcal {T}_{n-1}$ in $\textrm{fam}(X)$, so $\exp (\log (X))$ is a summand of *X*, and denote its complement by $X-\exp (\log (X))\subset \mathcal {T}_{n-1}$. Define $X>Y$ if either $\log (X)>\log (Y)$ or $\log (X)=\log (Y)$ and $X-\exp (\log (X))>Y-\exp (\log (Y))$.

By Lemma 2.29, there is a unique map

$$\begin{aligned} {{\,\textrm{Tree}\,}}:{{\,\textrm{Rat}\,}}(U)\cup \{0\}\rightarrow \mathcal {T} \end{aligned}$$

that maps 0 to $0_{\mathcal {T}}$. For $\alpha \in {{\,\textrm{Rat}\,}}(U)\cup \{0\}$, the image ${{\,\textrm{Tree}\,}}(\alpha )$ is called the *complexity* of $\alpha$.

#### Remark 2.31

If $V\leqslant U$ is a subgroup then by Lemma 2.29 there is a unique map

$$\begin{aligned}{{\,\textrm{Tree}\,}}_V:{{\,\textrm{Rat}\,}}(V)\cup \{0\}\rightarrow \mathcal {T}\end{aligned}$$

that maps 0 to $0_{\mathcal {T}}$. If $\alpha \in {{\,\textrm{Rat}\,}}(V)\cup \{0\}\subset {{\,\textrm{Rat}\,}}(U)\cup \{0\}$, then ${{\,\textrm{Tree}\,}}_V(\alpha )={{\,\textrm{Tree}\,}}(\alpha )$ for all $\alpha \in {{\,\textrm{Rat}\,}}(V)\cup \{0\}$, i.e., the complexity of $\alpha$ does not depend on whether we consider $\alpha$ as an element of ${{\,\textrm{Rat}\,}}(V)\cup \{0\}$ or ${{\,\textrm{Rat}\,}}(U)\cup \{0\}$.

### Tree complexity associated to groups

Let *G* be a locally indicable group, let $\tau :\mathbb {C}G \rightarrow \mathcal {D}_G$ be the natural embedding into the Linnell skew field, and let $\sigma :G \rightarrow \textrm{GL}_n(\mathbb {C})$ be a finite-dimensional representation.

For every subgroup $H\leqslant G$, we think of the Linnell skew field $\mathcal {D}_H$ as a subring of $\mathcal {D}_G$. Let ${\widetilde{\mathcal {D}}}_H$ be the division closure of $(\sigma \otimes _{\mathbb {C}}\tau )(\mathbb {C}H)$ in $M_n(\mathcal {D}_G)$. Note that by the definition of the division closure, $\widetilde{\mathcal {D}}_H$ is a subring of $M_n({\mathcal {D}}_H)$, that is,

3 $$\begin{aligned} {\widetilde{\mathcal {D}}}_H\leqslant M_n(\mathcal {D}_H). \end{aligned}$$

The map $\sigma \otimes _{\mathbb {C}}\tau$ is a ring homomorphism from $\mathbb {C}H$ to $M_n(\mathcal {D}_G)$. Then under the rational map given by Example 2.28, ${\widetilde{\mathcal {D}}}_H$ is a rational $\mathbb {C}^{\times }H$-semiring. Lemma 2.29 then gives a map

$$\begin{aligned}\Phi _H:{{\,\textrm{Rat}\,}}(\mathbb {C}^{\times }H)\cup \{0\}\rightarrow {\widetilde{\mathcal {D}}}_H.\end{aligned}$$

#### Lemma 2.32

The image ${{\,\textrm{im}\,}}(\Phi _H)$ equals ${\widetilde{\mathcal {D}}}_H$.

#### Proof

First note that ${{\,\textrm{im}\,}}(\Phi _H)$ is a ring. Indeed, since $-\textrm{Id}\in {{\,\textrm{im}\,}}(\Phi _H)$, for every $x\in {{\,\textrm{im}\,}}(\Phi _H)$, we also have $-x=(-1)\cdot x\in {{\,\textrm{im}\,}}(\Phi _H)$.

Second, note that the target of $\Phi _H$ is ${\widetilde{\mathcal {D}}}_H$, and so we automatically have the inclusion ${{\,\textrm{im}\,}}(\Phi _H)\subset {\widetilde{\mathcal {D}}}_H$. To prove the reverse containment, first note that ${{\,\textrm{im}\,}}(\Phi _H)$ contains $(\sigma \otimes _{\mathbb {C}}\tau )(\mathbb {C}H)$. Let $x\in {{\,\textrm{im}\,}}(\Phi _H)$. Then there exists $\alpha \in {{\,\textrm{Rat}\,}}(\mathbb {C}^{\times }H)\cup \{0\}$ such that $\Phi _H(\alpha )=x$. If *x* is invertible in $M_n(\mathcal {D}_G)$, then *x* is invertible in ${\widetilde{\mathcal {D}}}_H$, and then $x^{-1}=x^{\diamond }=\Phi _H(\alpha ^{\diamond })\in {{\,\textrm{im}\,}}(\Phi _H)$. So ${{\,\textrm{im}\,}}(\Phi _H)$ contains the division closure of $(\sigma \otimes _{\mathbb {C}}\tau )(\mathbb {C}H)$ in $M_n(\mathcal {D}_G)$, that is, ${\widetilde{\mathcal {D}}}_H$. $\square$

Let $\mathcal {T}$ be the set of finite rooted trees. As in Sect. 2.13, we get a map

$$\begin{aligned}{{\,\textrm{Tree}\,}}:{{\,\textrm{Rat}\,}}(\mathbb {C}^{\times }H)\cup \{0\}\rightarrow \mathcal {T}.\end{aligned}$$

The *H*-*complexity* of an element $x\in {\widetilde{\mathcal {D}}}_H$ is defined as

$$\begin{aligned}{{\,\textrm{Tree}\,}}_H(x)=\min \{{{\,\textrm{Tree}\,}}(\alpha )\mid \alpha \in {{\,\textrm{Rat}\,}}(\mathbb {C}^{\times }H)\cup \{0\}, \Phi _H(\alpha )=x\}.\end{aligned}$$

By Lemma 2.32, ${{\,\textrm{Tree}\,}}_H$ is defined on the whole of ${\widetilde{\mathcal {D}}}_H$. We say that

$$\begin{aligned}\alpha \in {{\,\textrm{Rat}\,}}(\mathbb {C}^{\times }H)\cup \{0\}\end{aligned}$$

*realizes the* *H-complexity* of *x* if $\Phi _H(\alpha )=x$ and ${{\,\textrm{Tree}\,}}(\alpha )={{\,\textrm{Tree}\,}}_H(x)$.

Now suppose that *H* is finitely generated and $H=N\rtimes \langle t \rangle$ for some normal subgroup $N\lhd H$ and infinite-order element $t\in H$. For simplicity, we denote $\tau (t) \in \mathcal {D}_H$ by *t* and $\sigma (t)\cdot \tau (t) \in {\widetilde{\mathcal {D}}}_H$ by *s*. Note that conjugation by *t* induces an automorphism $\mathcal {D}_N\rightarrow \mathcal {D}_N,x\mapsto txt^{-1}$. Indeed, $\mathcal {D}_N$ is the division closure of $\tau (\mathbb {C}N)$ in $\mathcal {D}_G$, and hence $t \mathcal {D}_N t^{-1}$ is the division closure of $t\tau (\mathbb {C}N)t^{-1} = \tau (t\mathbb {C}Nt^{-1}) = \tau (\mathbb {C}N)$. Therefore, $t \mathcal {D}_N t^{-1}$ and $\mathcal {D}_N$ are division closures of the same ring in $\mathcal {D}_G$, and hence coincide.

We now see that the conjugation $A\mapsto sAs^{-1}$ induces an automorphism of $M_n(\mathcal {D}_N)$. Also, similarly to the above proof, one can show that the conjugation $A\mapsto sAs^{-1}$ induces an automorphism of ${\widetilde{\mathcal {D}}}_N$. Therefore, we can form $\mathcal {D}_N(\!(t)\!)$, ${\widetilde{\mathcal {D}}}_N(\!(s)\!)$ and $M_n(\mathcal {D}_N)(\!(s)\!)$, the rings of twisted Laurent power series with twisting structures given by these conjugation automorphisms. It is a standard fact that $\mathcal {D}_H$ can be identified with a subring of $\mathcal {D}_N(\!(t)\!)$; it quickly follows for example from [28, Proposition 2.23]. It is clear that $M_n(\mathcal {D}_N(\!(t)\!))=M_n(\mathcal {D}_N)(\!(s)\!)$. Hence, the containment (3) implies that ${\widetilde{\mathcal {D}}}_H$ and ${\widetilde{\mathcal {D}}}_N$ can be identified with subrings of $M_n(\mathcal {D}_N)(\!(s)\!)$. The following is essentially [26, Proposition 5.1].

#### Proposition 2.33

Let $x\in {\widetilde{\mathcal {D}}}_H$ and assume that for every $0\ne y\in {\widetilde{\mathcal {D}}}_H$ such that ${{\,\textrm{Tree}\,}}_H(y)<{{\,\textrm{Tree}\,}}_H(x)$, *y* is invertible in ${\widetilde{\mathcal {D}}}_H$. Then $x\in {\widetilde{\mathcal {D}}}_N(\!(s)\!)$.

Moreover, write *x* as a Laurent power series

$$\begin{aligned}x=\sum _i x_is^i,\end{aligned}$$

where $x_i\in {\widetilde{\mathcal {D}}}_N$ for all *i*. Then

$$\begin{aligned}{{\,\textrm{Tree}\,}}_H(x_i)\leqslant {{\,\textrm{Tree}\,}}_H(x)\end{aligned}$$

for all *i*, and equality holds for some *i* if and only if $x=x_is^i$.

#### Proof

We will apply [26, Proposition 5.1] with

$$\begin{aligned}\mathcal {A}=M_n(\mathcal {D}_N),\quad \mathcal {P}=M_n(\mathcal {D}_N)(\!(s)\!), \quad \mathcal {D}_{N,\mathcal {P}}={\widetilde{\mathcal {D}}}_N, \quad \mathcal {D}_{H,\mathcal {P}}={\widetilde{\mathcal {D}}}_H\end{aligned}$$

in the notation of the proposition. We need to verify that ${\widetilde{\mathcal {D}}}_N$ (resp. ${\widetilde{\mathcal {D}}}_H$) is the division closure of $(\sigma \otimes _{\mathbb {C}}\tau )(\mathbb {C}N)$ (resp. $(\sigma \otimes _{\mathbb {C}}\tau )(\mathbb {C}H)$) in $M_n(\mathcal {D}_N)(\!(s)\!)$, where we think of $\sigma \otimes _{\mathbb {C}}\tau$ as a ring homomorphism from $\mathbb {C}N$ (resp. $\mathbb {C}H$) to $M_n(\mathcal {D}_N)(\!(s)\!)$.

Consider ${\widetilde{\mathcal {D}}}_H$. First, suppose that $y\in {\widetilde{\mathcal {D}}}_H$ is invertible in $M_n(\mathcal {D}_N)(\!(s)\!)=M_n(\mathcal {D}_N(\!(t)\!))$. By (3), the entries of the matrix *y* lie in the skew field $\mathcal {D}_H$. So *y* is invertible in $M_n(\mathcal {D}_H)$, and thus in $M_n(\mathcal {D}_G)$. So *y* is invertible in ${\widetilde{\mathcal {D}}}_H$. So ${\widetilde{\mathcal {D}}}_H$ contains the division closure of $(\sigma \otimes _{\mathbb {C}}\tau )(\mathbb {C}H)$ in $M_n(\mathcal {D}_N)(\!(s)\!)$, say *R*. As a subring of ${\widetilde{\mathcal {D}}}_H$, *R* can be identified with a subring of $M_n(\mathcal {D}_G)$. Let $z\in R$ be such that *z* is invertible in $M_n(\mathcal {D}_G)$. By (3), $z\in {\widetilde{\mathcal {D}}}_H$ is a matrix over $\mathcal {D}_H$. So *z* is invertible in $M_n(\mathcal {D}_H)$, and thus in $M_n(\mathcal {D}_N(\!(t)\!))$. So *z* is invertible in *R*. This implies that $R\geqslant {\widetilde{\mathcal {D}}}_H$, and therefore finishes the verification that ${\widetilde{\mathcal {D}}}_H$ is the division closure of $(\sigma \otimes _{\mathbb {C}}\tau )(\mathbb {C}H)$ in $M_n(\mathcal {D}_N)(\!(s)\!)$. The verification for ${\widetilde{\mathcal {D}}}_N$ is similar and straightforward.

By [26, Proposition 5.1], we have $x\in {\widetilde{\mathcal {D}}}_N(\!(s)\!)$. Write *x* as a Laurent power series $x=\sum _i x_is^i$, where $x_i\in {\widetilde{\mathcal {D}}}_N$ for all *i*. Now, [26, Proposition 5.1] implies that ${{\,\textrm{Tree}\,}}_H(x_is^i)\leqslant {{\,\textrm{Tree}\,}}_H(x)$ for all *i*, and equality holds for some *i* if and only if $x=x_is^i$. To finish the proof, simply note that for all $z\in {\widetilde{\mathcal {D}}}_N$ and all *i*, ${{\,\textrm{Tree}\,}}_H(z)={{\,\textrm{Tree}\,}}_H(zs^i)$. $\square$

## Agrarian invariants

The notion of agrarian groups was introduced in [27], but the idea dates back to Malcev [40]. The notion of agrarian invariants was later introduced and studied by Henneke and the first author in [19].

### Agrarian maps

Let *G* be a group. An *agrarian map* of *G* is a finite dimensional left linear representation $\sigma :G\rightarrow {{\,\textrm{GL}\,}}_n(D)$ of *G* over a skew field *D*, where $\textrm{GL}_n(D)$ denotes the group of invertible $n\times n$-matrices over *D*.

#### Remark 3.1

In [19], the authors define an agrarian map to be a 1-dimensional representation $G\rightarrow {{\,\textrm{GL}\,}}_1(D)$ over a skew field. As we will see in Examples 3.4 and 3.18, general finite-dimensional representations arise naturally in the study of twisted invariants. We therefore generalize the work of [19] and define an agrarian map to be a general finite-dimensional representation.

### Rationalization

If *G* is finitely generated, then it has a maximal free abelian quotient denoted $G_{\textrm{fab}}$. Let $q:G\twoheadrightarrow G_{\textrm{fab}}$ be the natural quotient map, and let $D G_{\textrm{fab}}$ and $\mathbb {Q}G_{\textrm{fab}}$ be the untwisted group rings. View $q:G\rightarrow {{\,\textrm{Ore}\,}}(\mathbb {Q}G_{\textrm{fab}})$ as another representation and form the tensor product representation

$$\begin{aligned}\sigma \otimes _{\mathbb {Z}} q:G\rightarrow {{\,\textrm{GL}\,}}_n({{\,\textrm{Ore}\,}}(DG_{\textrm{fab}})),~ (\sigma \otimes _{\mathbb {Z}} q)(g)=\sigma (g)q(g),\end{aligned}$$

called the *rationalization* of $\sigma$. (Since $\mathbb {Q}G_{\textrm{fab}}$ is commutative, ${{\,\textrm{Ore}\,}}(\mathbb {Q}G_{\textrm{fab}})$ is of course a familiar field of rational functions in multiple variables.)

For the rest of this subsection suppose $n=1$, i.e., we have a homomorphism $\sigma :G\rightarrow D^{\times }$. In [19], Henneke and the first author introduce the following rationalization of the agrarian map $\sigma$. Let $K=\ker q$. As explained in Example 2.3, by picking a set-theoretic section $s:G_{\textrm{fab}}\rightarrow G$, we obtain a twisted group ring $(\mathbb {Z}K)G_{\textrm{fab}}$ with a natural isomorphism $\mathbb {Z}G\cong (\mathbb {Z}K) G_{\textrm{fab}}$. The maps *s* and $\sigma$ together induce a twisted group ring structure $D *G_{\textrm{fab}}$ (here the notation is used to distinguish the twisted group ring $D *G_{\textrm{fab}}$ from the untwisted group ring $D G_{\textrm{fab}}$). The restriction $\sigma |_{\mathbb {Z}K}:\mathbb {Z}K\rightarrow D$ naturally induces a ring homomorphism $(\mathbb {Z}K) G_{\textrm{fab}}\rightarrow D *G_{\textrm{fab}}$ between the twisted group rings. Note that there is a natural embedding $D*G_{\textrm{fab}}\hookrightarrow {{\,\textrm{Ore}\,}}(D*G_{\textrm{fab}})$. By definition, the *HK-rationalization* of $\sigma$, denoted $\widetilde{\sigma }:\mathbb {Z}G\rightarrow {{\,\textrm{Ore}\,}}(D *G_{\textrm{fab}})$, is the composition

$$\begin{aligned}\mathbb {Z}G\cong (\mathbb {Z}K) G_{\textrm{fab}}\rightarrow D*G_{\textrm{fab}}\rightarrow {{\,\textrm{Ore}\,}}(D*G_{\textrm{fab}}).\end{aligned}$$

The following Lemma 3.2 can be easily extracted from the proof of [25, Proposition 3.5]. We provide the proof for the convenience of the reader. Roughly speaking, Lemma 3.2 implies that $\sigma \otimes _{\mathbb {Z}}q$ and $\widetilde{\sigma }$ are equivalent for the purpose of defining agrarian invariants. The reader is referred to Remark 3.15 below for the precise meaning of this equivalence.

#### Lemma 3.2

There is an isomorphism $\alpha :{{\,\textrm{Ore}\,}}(D*G_{\textrm{fab}}) \rightarrow {{\,\textrm{Ore}\,}}(DG_{\textrm{fab}})$ such that the following hold.

(i) $$\alpha \circ \widetilde{\sigma }=\sigma \otimes _{\mathbb {Z}}q$$.
(ii) For all $x\in D G_{\textrm{fab}}$, view *x* and $\alpha (x)$ as functions $x,\alpha (x):G_{\textrm{fab}}\rightarrow D$. Then ${{\,\textrm{supp}\,}}(x)={{\,\textrm{supp}\,}}(\alpha (x))$.

#### Proof

For all $d\in D$ and $h\in G_{\textrm{fab}}$, let

$$\begin{aligned}\alpha (d*h)=d\cdot \sigma (s(h))\cdot h,\end{aligned}$$

where the product $d*h$ on the left-hand side is considered as an element of $D *G_{\textrm{fab}}$, and the right-hand side product is considered as an element of $DG_{\textrm{fab}}$. By extending $\alpha$ linearly across $D *G_{\textrm{fab}}$ we get a map $\alpha :D *G_{\textrm{fab}} \rightarrow DG_{\textrm{fab}}$. Item (ii) follows immediately.

We check that $\alpha$ is a ring homomorphism. First, $\alpha$ obviously preserves addition. Second, note that the identity of $D*G_{\textrm{fab}}$ is $\sigma (s(1))^{-1}*1$. Indeed, for all $d\in D$ and $h\in G_{\textrm{fab}}$,

$$\begin{aligned} \Big (\sigma \big (s(1)\big )^{-1}*1\Big )\cdot (d*h)&=\Big (\sigma \big (s(1)\big )^{-1}\sigma \big (s(1)\big )d\sigma \big (s(1)\big )^{-1}\\&\quad \sigma \big (s(1)\big )\sigma \big (s(h)\big )\sigma \big (s(h)\big )^{-1}\Big )*h\\&=d*h. \end{aligned}$$

Since $\alpha (\sigma (s(1))^{-1}*1)=\sigma (s(1))^{-1}\cdot \sigma (s(1))\cdot 1=1$, the function $\alpha$ preserves the identity.

Third, for all $d_1,d_2\in D$ and $h_1,h_2\in G_{\textrm{fab}}$,

$$\begin{aligned} \alpha \big ((d_1*h_1)\cdot (d_2*h_2)\big )&=\alpha \Big (\Big (d_1\sigma \big (s(h_1)\big )d_2\sigma \big (s(h_1)\big )^{-1}\\&\quad \sigma \big (s(h_1)\big )\sigma \big (s(h_2)\big )\sigma \big (s(h_1h_2)\big )^{-1}\Big )*(h_1h_2)\Big )\\&=d_1\sigma \big (s(h_1)\big )d_2\sigma \big (s(h_2)\big )\cdot (h_1h_2), \\ \alpha (d_1*h_1)\cdot \alpha (d_2*h_2)&=d_1\sigma \big (s(h_1)\big )h_1\cdot d_2\sigma \big (s(h_2)\big )h_2 \\&=d_1\sigma \big (s(h_1)\big )d_2\sigma \big (s(h_2)\big )\cdot (h_1h_2). \end{aligned}$$

Thus, $\alpha$ preserves multiplication. This show that $\alpha$ is a ring homomorphism.

We define an inverse of $\alpha$ as follows. Let $\beta :DG_{\textrm{fab}}\rightarrow D *G_{\textrm{fab}}$ be given by setting

$$\begin{aligned}\beta (dh)=\Big (d\sigma \big (s(h)\big )^{-1}\Big )*h,\end{aligned}$$

for all $d\in D,h\in G_{\textrm{fab}}$, and then extending linearly across $DG_{\textrm{fab}}$. It is easy to check that $\beta$ is indeed the inverse of $\alpha$, and thus $\alpha :D*G_{\textrm{fab}} \rightarrow DG_{\textrm{fab}}$ is a ring isomorphism.

Therefore, $\alpha$ extends to a ring isomorphism between the Ore localizations. It remains to check item (i). Let $g\in G$, let $h=q(g)\in G_{\textrm{fab}}$. Then

$$\begin{aligned}\alpha (\widetilde{\sigma }(g))=\alpha (\sigma (gs(h)^{-1})*h)=\sigma (gs(h)^{-1})\sigma (s(h))\cdot h=\sigma (g)\cdot h=\sigma (g)q(g).\end{aligned}$$

as desired. $$\square $$

### Agrarian Betti numbers

In the sequel, tensor products happen between left and right modules, i.e., if *R* is a ring, then $M\otimes _R N$ is the tensor product of a right *R*-module *M* and a left *R*-module *N*. Moreover, if $S\subset M,T\subset N$ are not submodules, then we define

$$\begin{aligned} S\otimes _R T=\{s\otimes t\mid s\in S, t\in T\}. \end{aligned}$$

Let $(C_{*},\partial _{*})$ be a chain complex of free right $\mathbb {Z}G$-modules.

#### Convention 3.3

In the sequel, $D^n$ will denote the *n*-dimension right *D*-module with column vectors as elements. We will write $D_{\sigma }^n$ for the $\mathbb {Z}G$-*D*-bimodule that is $D^n$ as a set with the left action induced by $\sigma$ (i.e., $g\cdot v=\sigma (g)\cdot v$ for all $g\in G,v\in D^n_{\sigma }$) and the right action given by coordinate-wise multiplication.

If *G* is finitely generated, let $q:G\rightarrow G_{\textrm{fab}}$ be the natural quotient map onto the maximal free abelian quotient $G_{\textrm{fab}}$ of *G* and let *X* be a basis of the free abelian group $G_{\textrm{fab}}$. For simplicity we will write $({{\,\textrm{Ore}\,}}(DG_{\textrm{fab}}))^n_{\sigma }$ (resp. $(D(X))_{\sigma }^n$) for $({{\,\textrm{Ore}\,}}(DG_{\textrm{fab}}))^n_{\sigma \otimes _{\mathbb {Z}}q}$ (resp. $(D(X))_{\sigma \otimes _{\mathbb {Z}}q}^n$). Note that the left action of *G* on $({{\,\textrm{Ore}\,}}(DG_{\textrm{fab}}))^n_{\sigma }\cong (D(X))_{\sigma }^n$ is given by $g\cdot v=\sigma (g)q(g)\cdot v$ for all $g\in G,v\in ({{\,\textrm{Ore}\,}}(DG_{\textrm{fab}}))^n_{\sigma }\cong (D(X))_{\sigma }^n$.

Tensoring $C_{*}$ with $D_{\sigma }^n$ over $\mathbb {Z}G$ gives rise to a chain complex $C_{*}\otimes _{\mathbb {Z}G} D_{\sigma }^n$, which is a complex of right *D*-modules. So $H_{*}(C_{*}\otimes _{\mathbb {Z}G} D_{\sigma }^n)$ is also a right *D*-module. The $i^{th}$ $\sigma$*-agrarian Betti number* of $C_{*}$ is

$$\begin{aligned}b^{\sigma }_i(C_{*})=\dim _D H_i(C_{*}\otimes _{\mathbb {Z}G} D_{\sigma }^n).\end{aligned}$$

If $C_{*}$ is a projective resolution of $\mathbb {Z}$ over $\mathbb {Z}G$, then we obtain the $i^{th}$ $\sigma$*-agrarian Betti number* of *G*:

$$\begin{aligned}b^{\sigma }_i(G)=\dim _D H_i(G, D_{\sigma }^n)\end{aligned}$$

where the homology is computed with respect to the representation $\sigma$.

#### Example 3.4

(Twisted $\ell ^2$-Betti numbers) Suppose that *G* is locally indicable (twisted $\ell ^2$-Betti numbers can be defined for any group, but for the purpose of this paper we restrict ourselves to locally indicable ones). Let $\eta :G\rightarrow {{\,\textrm{GL}\,}}_n(\mathbb {C})$ be a finite-dimensional complex representation. Recall that $\mathcal {D}_G$ denotes the Linnell skew field of *G* and $\tau :\mathbb {Z}G\rightarrow \mathcal {D}_G$ denotes the natural embedding. Let

$$\begin{aligned}\sigma =\eta \otimes _{\mathbb {C}}\tau :G\rightarrow {{\,\textrm{GL}\,}}_n(\mathcal {D}_G),~\sigma (g)=\eta (g)\tau (g).\end{aligned}$$

Then the $i^{th}$ *twisted* $\ell ^2$*-Betti number* of $C_{*}$ with respect to $\eta$ is

$$\begin{aligned}b^{(2),\eta }_{i}(C_{*})=b^{\sigma }_i(C_{*}).\end{aligned}$$

If $\eta$ is the trivial representation $G\rightarrow {{\,\textrm{GL}\,}}_1(\mathbb {C})$ that sends every $g\in G$ to 1, then we denote $b^{(2),\eta }_{i}(C_{*})$ by $b^{(2)}_i(C_{*})$ and call it the $i^{th}$ *(untwisted)* $\ell ^2$*-Betti number* of $C_{*}$.

#### Remark 3.5

A widely used definition of $\ell ^2$-Betti numbers is the following. Let $\mathcal {N}(G)$ be the von Neumann alebra of *G*. Then

$$\begin{aligned}b^{(2)}_i(C_{*})=\dim _{\mathcal {N}(G)}H_i(C_{*}\otimes _{\mathbb {Z}G}\mathcal {N}(G)),\end{aligned}$$

where $\dim _{\mathcal {N}(G)}$ denotes the von Neumann dimension. By [15, Theorem 3.6 (2)], this definition is the same as the one in Example 3.4 when *G* is locally indicable.

#### Lemma 3.6

Suppose that *G* is finitely generated with $G_{\textrm{fab}}$ non-trivial. Then $b^{\sigma \otimes _{\mathbb {Z}} q}_0(G)=0$.

#### Proof

There exists a presentation $G=\langle X \mid \mathcal {R} \rangle$ with an element $x\in X$ such that *q*(*x*) is an element of a basis *Y* of $G_{\textrm{fab}}$. Below, we denote ${{\,\textrm{Ore}\,}}(DG_{\textrm{fab}})$ by *D*(*Y*) and when we talk about the *q*(*x*)-order, we mean the order with respect to *Y*.

Construct a *K*(*G*, 1) CW-complex *BG* from the presentation complex of $G=\langle X \mid \mathcal {R} \rangle$ by adding cells of dimension greater than or equal to 3, and let *EG* be the universal cover of *BG*. Consider the cellular chain complex of *EG* consisting of right *G*-modules:

$$\begin{aligned}\cdots \rightarrow C_1\xrightarrow []{\partial _1}C_0\rightarrow 0.\end{aligned}$$

Let *p* be the unique 0-dimensional cell of *BG* and let *e* be the edge of *BG* labeled by *x*. There exists a lift ${\widetilde{p}}$ (resp. ${\widetilde{e}}$) of *p* (resp. *e*) such that

$$\begin{aligned}\partial _1({\widetilde{e}})={{\widetilde{p}}}\cdot (1-x).\end{aligned}$$

Consider the map

$$\begin{aligned}\partial _1\otimes _{\mathbb {Z}G} \textrm{id}_{(D(Y))_{\sigma }^n}:C_1\otimes _{\mathbb {Z}G} ((D(Y))_{\sigma }^n\rightarrow C_0\otimes _{\mathbb {Z}G} (D(Y))_{\sigma }^n.\end{aligned}$$

Let

$$\begin{aligned}\partial '_1:({\widetilde{e}}\cdot (\mathbb {Z}G))\otimes _{\mathbb {Z}G} ((D(Y))_{\sigma }^n\rightarrow C_0\otimes _{\mathbb {Z}G} ((D(Y))_{\sigma }^n\end{aligned}$$

be the restriction of $\partial _1\otimes _{\mathbb {Z}G} \textrm{id}_{((D(Y))_{\sigma }^n}$. Note that for every column vector $v\in (D(Y))_{\sigma }^n$, we have

$$\begin{aligned}\partial '_1({{\widetilde{e}}}\otimes v)=({{\widetilde{p}}} \cdot (1-x))\otimes v={{\widetilde{p}}} \otimes (v-\sigma (x)\cdot q(x)\cdot v).\end{aligned}$$

Let $\mathcal {B}$ be the standard *D*(*Y*)-basis of $((D(Y))_{\sigma }^n$. Then under the bases $\{{\widetilde{e}}\}\otimes _{\mathbb {Z}G} \mathcal {B}$ and $\{{\widetilde{p}}\}\otimes _{\mathbb {Z}G} \mathcal {B}$, the matrix representative of $\partial '_1$ has the form

$$\begin{aligned}\textrm{Id}-\sigma (x)\cdot q(x).\end{aligned}$$

Since $\sigma (x)$ is a matrix over *D*, each entry of $\sigma (x)q(x)$ has *q*(*x*)-order at least 1. So Lemma 2.13 implies that $\textrm{Id}-\sigma (x)\cdot q(x)$ is invertible, and thus $\partial '_1$ is surjective. It follows that $\partial _1\otimes _{\mathbb {Z}G} \textrm{id}_{(D(Y))_{\sigma }^n}$ is surjective as well and thus $b^{\sigma \otimes _{\mathbb {Z}} q}_0(G)=0$. $\square$

The $\sigma$*-agrarian Euler characteristic* of $C_{*}$ is

$$\begin{aligned}\chi ^{\sigma }(C_{*})=\sum ^{\infty }_{i=0}(-1)^i b^{\sigma }_i(C_{*})\end{aligned}$$

provided that the sum is well defined, i.e., only finitely many of the values $b^{\sigma }_i(C_{*})$ are non-zero, and all non-zero terms are finite.

If in addition $C_{*}$ is a free resolution of $\mathbb {Z}$ over $\mathbb {Z}G$, then we obtain the $\sigma$*-agrarian Euler characteristic* of *G*:

$$\begin{aligned}\chi ^{\sigma }(G)=\sum ^{\infty }_{i=0}(-1)^i b^{\sigma }_i(G).\end{aligned}$$

#### Proposition 3.7

If $C_{*}$ is *finite*, i.e., each $C_i$ has finite rank and there are only finitely many non-zero modules $C_i$, then

$$\begin{aligned}\sum ^{\infty }_{i=0}(-1)^i b^{\sigma }_i(C_{*})=n\cdot \sum ^{\infty }_{i=0}(-1)^i{{\,\textrm{rk}\,}}_{\mathbb {Z}G}C_i=n\cdot \chi (C_{*}),\end{aligned}$$

where $\chi (C_{*})$ denotes the usual Euler characteristic of $C_{*}$. In particular,

$$\begin{aligned}\chi ^{\sigma }(G)=n\cdot \chi (G)\end{aligned}$$

if *G* is of type *F*, i.e., there is a *K*(*G*, 1) space that is a finite CW-complex.

#### Proof

For all *i*, decompose $C_i\otimes _{\mathbb {Z}G} D_{\sigma }^n$ as

$$\begin{aligned}C_i\otimes _{\mathbb {Z}G} D_{\sigma }^n=B_i\oplus H_i\oplus C'_i,\end{aligned}$$

where $B_i$ is the image of $\partial _i\otimes _{\mathbb {Z}G} \textrm{id}_{D_{\sigma }^n}$, $H_i\cong H_i(C_{*}\otimes _{\mathbb {Z}G} D_{\sigma }^n)$, and $\partial _i\otimes _{\mathbb {Z}G} \textrm{id}_{D_{\sigma }^n}$ maps $C'_i$ isomorphically onto $B_{i-1}$ and restricts to the trivial map on $B_i \oplus H_i$. The decomposition is possible as $C_i\otimes _{\mathbb {Z}G} D_{\sigma }^n$ is a right-module over the skew field *D*. Then

$$\begin{aligned}\dim _D B_i=\dim _D C'_{i+1}.\end{aligned}$$

Therefore,

$$\begin{aligned}\sum ^{\infty }_{i=0}(-1)^i \dim _D H_i=\sum ^{\infty }_{i=0} (-1)^i \dim _D (C_i\otimes _{\mathbb {Z}G} D_{\sigma }^n)=n\cdot \sum ^{\infty }_{i=0} (-1)^i {{\,\textrm{rk}\,}}_{\mathbb {Z}G}(C_i).\end{aligned}$$

$$\square $$

#### Proposition 3.8

Suppose that *G* is finitely generated and is the fundamental group of the mapping torus $T_f$ of a cellular self-map $f:Y\rightarrow Y$ of a connected CW-complex *Y* with finite *d*-skeleton. Let $C_{*}$ be the cellular chain complex of the universal cover $\widetilde{T_f}$ of $T_f$. Then for $i\leqslant d$,

$$\begin{aligned}b^{\sigma \otimes _{\mathbb {Z}} q}_i(C_{*})=0.\end{aligned}$$

#### Proof

For each *i*, by lifting each *i*-cell of $Y\subset T_f$ to an *i*-cell in the universal cover $\widetilde{T_f}$, we obtain a set $\mathcal {B}_i\subset C_i$. Let

$$\begin{aligned}\mathcal {A}_{i+1}=\{\Delta \times [0,1]\mid \Delta \in \mathcal {B}_i\},\\A_{i+1}={{\,\textrm{span}\,}}_{\mathbb {Z}G}\mathcal {A}_i,~~B_i={{\,\textrm{span}\,}}_{\mathbb {Z}G}\mathcal {B}_i.\end{aligned}$$

(Here the subscript keeps track of dimension and so we use $\mathcal {A}_{i+1}$ instead of $\mathcal {A}_i$.) Then $\mathcal {A}_i\cup \mathcal {B}_i$ is a $\mathbb {Z}G$-basis of $C_i=A_i\oplus B_i$. Let *V* be the standard basis of $({{\,\textrm{Ore}\,}}(D G_\textrm{fab}))_{\sigma }^n$. Then $\mathcal {A}_i\otimes _{\mathbb {Z}G} V$ (resp. $\mathcal {B}_i\otimes _{\mathbb {Z}G} V$) is an ${{\,\textrm{Ore}\,}}(D G_\textrm{fab})$-basis of $A_i\otimes _{\mathbb {Z}G} ({{\,\textrm{Ore}\,}}(D G_\textrm{fab}))_{\sigma }^n$ (resp. $B_i\otimes _{\mathbb {Z}G} ({{\,\textrm{Ore}\,}}(D G_\textrm{fab}))_{\sigma }^n$).

Now suppose $i\leqslant d$. Let

$$\begin{aligned}P_i:C_i\otimes _{\mathbb {Z}G} ({{\,\textrm{Ore}\,}}(D G_\textrm{fab}))_{\sigma }^n\rightarrow B_i\otimes _{\mathbb {Z}G} ({{\,\textrm{Ore}\,}}(D G_\textrm{fab}))_{\sigma }^n\end{aligned}$$

be the projection corresponding to the direct sum decomposition

$$\begin{aligned}C_i\otimes _{\mathbb {Z}G} ({{\,\textrm{Ore}\,}}(D G_\textrm{fab}))_{\sigma }^n=(A_i\otimes _{\mathbb {Z}G} ({{\,\textrm{Ore}\,}}(D G_\textrm{fab}))_{\sigma }^n)\oplus (B_i\otimes _{\mathbb {Z}G} ({{\,\textrm{Ore}\,}}(D G_\textrm{fab}))_{\sigma }^n),\end{aligned}$$

and let $\partial '_{i+1}$ be the restriction of $\partial _{i+1}\otimes _{\mathbb {Z}G} \textrm{id}_{({{\,\textrm{Ore}\,}}(D G_\textrm{fab}))_{\sigma }^n}$ to $A_{i+1}$.

The natural map $T_f\rightarrow S^1$ that maps *Y* to a single point induces a group homomorphism $G\rightarrow \mathbb {Z}$, which factors through a homomorphism $G_{\textrm{fab}}\rightarrow \mathbb {Z}$. Let *X* be a basis of the free abelian group $G_{\textrm{fab}}$ such that there exists $t\in X$ that is mapped to a generator of $\mathbb {Z}$ by the homomorphism $G_{\textrm{fab}}\rightarrow \mathbb {Z}$. Below, we denote ${{\,\textrm{Ore}\,}}(DG_{\textrm{fab}})$ by *D*(*X*) and when we talk about *t*-order, we mean the order with respect to the basis *X*.

The matrix representative of $P_i\circ \partial '_{i+1}$ under the bases $\mathcal {A}_{i+1}\otimes _{\mathbb {Z}G} V$ and $\mathcal {B}_i\otimes _{\mathbb {Z}G} V$ has the form $\textrm{Id}+M\cdot t$ where *M* is a matrix over $D[X\smallsetminus \{t\}]$. In particular, each entry of $M\cdot t$ has *t*-order at least 1. By Lemma 2.13, $P_i\otimes _{\mathbb {Z}G} \partial '_{p+1}$ is invertible. In particular,

$$\begin{aligned} \dim _{D(X)}{{\,\textrm{im}\,}}(\partial _{i+1}\otimes _{\mathbb {Z}G} \textrm{id}_{(D(X))_{\sigma }^n})&\geqslant \dim _{D(X)}{{\,\textrm{im}\,}}(\partial '_{i+1}\otimes _{\mathbb {Z}G} \textrm{id}_{(D(X))_{\sigma }^n}) \\&= \dim _{D(X)} (B_i\otimes _{\mathbb {Z}G} (D(X))_{\sigma }^n) \\&= n\cdot |\mathcal {B}_i|. \end{aligned}$$

The same argument with $i-1$ in place of *i* shows that

$$\begin{aligned}&\hspace{-10mm} \dim _{D(X)} \ker (\partial _i\otimes _{\mathbb {Z}G} \textrm{id}_{(D(X))_{\sigma }^n})\\&= \dim _{D(X)}C_i\otimes _{\mathbb {Z}G} (D(X))_{\sigma }^n-\dim _{D(X)} {{\,\textrm{im}\,}}(\partial _i\otimes _{\mathbb {Z}G} \textrm{id}_{(D(X))_{\sigma }^n})\\&\leqslant n\cdot (|\mathcal {A}_i|+|\mathcal {B}_i|)-n\cdot |\mathcal {B}_{i-1}|\\&= n\cdot |\mathcal {B}_i|, \end{aligned}$$

where the last equality follows from $|\mathcal {A}_i|=|\mathcal {B}_{i-1}|$.

So

$$\begin{aligned} b^{\sigma \otimes _{\mathbb {Z}} q}_i(C_{*})&=\dim _{D(X)} \ker (\partial _i\otimes _{\mathbb {Z}G} \textrm{id}_{(D(X))_{\sigma }^n})-\dim _{D(X)}{{\,\textrm{im}\,}}(\partial _{i+1}\otimes _{\mathbb {Z}G} \textrm{id}_{(D(X))_{\sigma }^n})\\&\leqslant 0. \end{aligned}$$

As the reverse inequality automatically holds, the desired result follows. $\square$

#### Corollary 3.9

Suppose that *G* is a (type *F*)-by-(infinite cyclic) group. Then $b^{\sigma \otimes _{\mathbb {Z}} q}_{*} (G)\,{=}\,0$.

### Agrarian torsion

Suppose that $C_{*}$ is finite, that it comes with a preferred basis $\mathcal {B}_{C_{*}}$, and that $C_{*}$ is $\sigma$*-acyclic*, i.e., $b^{\sigma }_i(C_{*})=0$ for all *i*, which implies that $C_*\otimes _{\mathbb {Z}G} D_{\sigma }^n$ is contractible (see, e.g., [44, Proposition 1.7.4]). Let $\gamma$ (resp. *d*) be a chain contraction (resp. the boundary map) of $C_{*}\otimes _{\mathbb {Z}G} D_{\sigma }^n$. Then $d+\gamma :C_{\textrm{even}}\otimes _{\mathbb {Z}G} D_{\sigma }^n \rightarrow C_{\textrm{odd}}\otimes _{\mathbb {Z}G} D_{\sigma }^n$ is an isomorphism of right *D*-modules, where $C_{\textrm{even}}$ (resp. $C_{\textrm{odd}}$) is the direct sum of the even (resp. odd) dimensional components of $C_{*}$. Tensoring the preferred basis of $C_{*}$ with the standard basis *V* of $D_{\sigma }^n$ gives rise to a preferred basis $\mathcal {B}_{*}=\mathcal {B}_{C_{*}}\otimes _{\mathbb {Z}G}V$ for $C_{*}\otimes _{\mathbb {Z}G} D_{\sigma }^n$. Represent $d+\gamma$ by a matrix *M* over *D* using $\mathcal {B}_{*}$. The $\sigma$*-agrarian torsion*, denoted by $\rho _{\sigma }(C_{*})$, is the Dieudonné determinant ${{\,\mathrm{\det }\,}}_D M$.

#### Remark 3.10

By [7, (15.3)], the value of $\rho _{\sigma }(C_{*})$ does not depend on the choice of $\gamma$. In more details, the chain complex $C_{*}\otimes _{\mathbb {Z}G} D_{\sigma }^n$ is an acyclic $(M_n(D),\{\textrm{Id}\})$-complex in the notation of [7, (15.3)], which states that different choices of the chain contraction yield the same Whitehead torsion. As the agrarian torsion is the Dieudonné determinant of the Whitehead torsion, it does not depend on the choice of the chain contraction.

For computational purposes we record the following:

#### Remark 3.11

Suppose that $\mathcal {B}'_{*}$ is another basis of $C_{*}\otimes _{\mathbb {Z}G} D_{\sigma }^n$ and the change of basis matrix from $\mathcal {B}_{*}$ to $\mathcal {B}'_{*}$ has Dieudonné determinant $\pm 1$. Let *N* be the matrix representative over $d+\gamma$ under the new basis $\mathcal {B}'_{*}$. Then ${{\,\mathrm{\det }\,}}_D N=\pm {{\,\mathrm{\det }\,}}_D M$, since the Dieudonné determinant is multiplicative.

#### Remark 3.12

Let $f:A\rightarrow B$ be a homomorphism between based finite-rank free right $\mathbb {Z}G$-modules and suppose the matrix representative of *f* under the chosen bases is *M* (so *f* coincides with left-multiplication by *M*). Tensoring these bases with the standard basis for $D_{\sigma }^n$ yields bases for $A\otimes _{\mathbb {Z}G} D_{\sigma }^n$ and $B\otimes _{\mathbb {Z}G} D_{\sigma }^n$. The matrix representative of $f\otimes _{\mathbb {Z}G} \textrm{id}_D$ under these bases is given by $\sigma (M)$ (since $A\otimes _{\mathbb {Z}G} D_{\sigma }^n$ and $B\otimes _{\mathbb {Z}G} D_{\sigma }^n$ are right *D*-modules, $f\otimes _{\mathbb {Z}G} \textrm{id}_D$ is given by left-multiplication by $\sigma (M)$). Here, $\sigma (M)$ is the matrix obtained by applying $\sigma$ to each entry of *M*.

### Agrarian polytope and agrarian norm

Now further suppose that *G* is finitely generated. Recall that there is a natural map $q :G\twoheadrightarrow G_{\textrm{fab}}$ of *G* onto its maximal free abelianization $G_{\textrm{fab}}$. Let $\sigma :G\rightarrow {{\,\textrm{GL}\,}}_n(D)$ be a representation over a skew field *D*. Suppose that $C_{*}$ is $(\sigma \otimes _{\mathbb {Z}} q)$-acyclic. Then the $\sigma$*-agrarian polytope* with respect to $C_{*}$ is $P(\rho _{\sigma \otimes _{\mathbb {Z}} q}(C_{*}))$, where

$$\begin{aligned}P:({{\,\textrm{Ore}\,}}(DG_{\textrm{fab}}))^{\times }_{\textrm{ab}}\rightarrow \mathcal {P}(G_{\textrm{fab}})\end{aligned}$$

is the polytope homomorphism defined in Sect. 2.6. Recall that $P(\rho _{\sigma \otimes _{\mathbb {Z}} q}(C_{*}))$ is a difference of two Newton polytopes: $P(\rho _{\sigma \otimes _{\mathbb {Z}} q}(C_{*}))=P_1-P_2$. Let $\phi \in H^1(G,\mathbb {Z})$ be a character. The $\sigma$*-agrarian norm* of $\phi$ is defined as

$$\begin{aligned} \Vert \phi \Vert _{\sigma ,C_{*}}&=\max \big \{k{{\,\textrm{ord}\,}}_x(z)\mid z\in P_1\}-\min \big \{k{{\,\textrm{ord}\,}}_x(z)\mid z\in P_1\}\\&-\max \big \{k{{\,\textrm{ord}\,}}_x(z)\mid z\in P_2\}+\min \big \{k{{\,\textrm{ord}\,}}_x(z)\mid z\in P_2\}. \end{aligned}$$

Note that the maxima and minima exist as both $P_1$ and $P_2$ are compact.

In general, the agrarian norm need not be a semi-norm, but we will prove in Sect. 6 that it is a semi-norm in many interesting cases, justifying the terminology. Our proof relies on the following observation:

#### Lemma 3.13

If $P(\rho _{\sigma \otimes _{\mathbb {Z}} q}(C_{*}))$ is a single polytope, i.e., $P_2=0$, then $\Vert \phi \Vert _{\sigma ,C_{*}}$ is a semi-norm.

It will be convenient to work with a second definition of $\Vert \cdot \Vert _{\sigma ,C_{*}}$: First find a character $\psi \in H^1(G,\mathbb {Z})$ such that $\phi =k\psi$ for some $k\in \mathbb {N}$ and $\psi$ is a primitive integral character. Choose a basis *X* of $G_{\textrm{fab}}$ such that there is an $x\in X$ with $\psi (x)=1$ and $\psi (y)=0$ for all $y\in X\smallsetminus \{x\}$. Define

$$\begin{aligned}\Vert \phi \Vert _{\sigma ,C_{*}}=k\cdot \deg _x \rho _{\sigma \otimes _{\mathbb {Z}} q}(C_{*}),\end{aligned}$$

where $\deg _x$ is the notion introduced in Sect. 2.4 and is computed with respect to the basis *X*.

#### Remark 3.14

The equivalence between the two definitions of $\Vert \cdot \Vert _{\sigma ,C_{*}}$ can be seen as follows. For every $z\in P_1\cup P_2$, the value $\psi (z)$ is computed by first writing *z* as a monomial in terms of the elements of *X*, and then letting $\psi (z)$ to be the power of *x* in this monomial, i.e.,

$$\begin{aligned} \psi (z)={{\,\textrm{ord}\,}}_x(z), \end{aligned}$$

where ${{\,\textrm{ord}\,}}_x$ is computed with respect to the basis *X*. Therefore,

$$\begin{aligned} \Vert \phi \Vert _{\sigma ,C_{*}}&=\max \big \{k{{\,\textrm{ord}\,}}_x(z)\mid z\in P_1\}-\min \big \{k{{\,\textrm{ord}\,}}_x(z)\mid z\in P_1\}\\&-\max \big \{k{{\,\textrm{ord}\,}}_x(z)\mid z\in P_2\}+\min \big \{k{{\,\textrm{ord}\,}}_x(z)\mid z\in P_2\}\\&=k\cdot \deg _x \rho _{\sigma \otimes _{\mathbb {Z}} q}(C_{*}). \end{aligned}$$

#### Remark 3.15

In the case $n=1$, [19] provides an alternative definition for the agrarian polytope, which we call the *HK-polytope* for $\sigma$ for the moment. The HK-polytope for $\sigma$ is defined using $\widetilde{\sigma }:G \rightarrow {{\,\textrm{Ore}\,}}(D *G_{\textrm{fab}})$, the HK-rationalization of $\sigma$, where $D *G_{\textrm{fab}}$ denotes the twisted group ring of Sect. 3.2. We sketch the construction here and refer the reader to [19] for details. The HK-polytope is defined only when $b^{\widetilde{\sigma }}_i(C_{*})=0$ for all *i*, so let us assume this is indeed the case. Generalizing Sect. 2.6, one can define a polytope homomorphism ${{\widetilde{P}}}:{{\,\textrm{Ore}\,}}(D *G_{\textrm{fab}})^{\times }_{\textrm{ab}}\rightarrow \mathcal {P}(G_{\textrm{fab}})$. Then the HK-polytope for $\sigma$ is ${{\widetilde{P}}}(\rho _{\widetilde{\sigma }}(C_{*}))$, where $\rho _{\widetilde{\sigma }}$ denotes the $\widetilde{\sigma }$-agrarian torsion.

The isomorphism $\alpha :{{\,\textrm{Ore}\,}}(D *G_{\textrm{fab}})\rightarrow {{\,\textrm{Ore}\,}}(DG_{\textrm{fab}})$ provided by Lemma 3.2 implies that $b^{\widetilde{\sigma }}_i(C_{*})=0$ if and only if $b^{\sigma \otimes _{\mathbb {Z}}q}_i(C_{*})=0$. So the HK-polytope for $\sigma$ is well defined if and only if our $\sigma$-agrarian polytope is well defined. Moreover, let $x\in D *G_{\textrm{fab}}$ and consider $\alpha (x)$. View *x* and $\alpha (x)$ as functions $x,\alpha (x):G_{\textrm{fab}}\rightarrow D$. Then ${{\,\textrm{supp}\,}}(x)={{\,\textrm{supp}\,}}(\alpha (x))$. It follows that the HK-polytope coincides with our agrarian polytope. The benefit of our approach is that it only uses the untwisted group ring, which is computationally simpler.

Before giving examples of the agrarian norm we would like to first prove its homotopy invariance. The proof of the following proposition combines ideas of [19] and [27].

#### Proposition 3.16

(Homotopy invariance) Let $C_{*},C'_{*}$ be homotopy equivalent finite based chain complexes of free $\mathbb {Z}G$-modules. Suppose that $C_{*}$ is $(\sigma \otimes _{\mathbb {Z}} q)$-acyclic. Then so is $C'_{*}$ and there is an equality between the agrarian polytopes

$$\begin{aligned}P(\rho _{\sigma \otimes _{\mathbb {Z}}q}(C_{*}))=P(\rho _{\sigma \otimes _{\mathbb {Z}}q}(C'_{*})).\end{aligned}$$

In particular, there is an equality between the corresponding agrarian norms

$$\begin{aligned}\Vert \cdot \Vert _{\sigma ,C_{*}}=\Vert \cdot \Vert _{\sigma ,C'_{*}}.\end{aligned}$$

#### Proof

For simplicity, denote ${{\,\textrm{Ore}\,}}(DG_{\textrm{fab}})$ by *E* and write $E_{\sigma }^n$ for the *G*-*E*-bimodule which is the same as $E^n$ as a set with the left *G*-action given by $\sigma \otimes _{\mathbb {Z}}q$ and a right *E*-module structure given by coordinate-wise multiplication. Let $f:C_{*}\rightarrow C'_{*}$ be a (chain) homotopy equivalence. Then $f\otimes _{\mathbb {Z}G} \textrm{id}_{E_{\sigma }^n}$ is a homotopy equivalence between $C_{*}\otimes _{\mathbb {Z}G} E_{\sigma }^n$ and $C'_{*}\otimes _{\mathbb {Z}G} E_{\sigma }^n$. Thus, ${C'_{*}\otimes _{\mathbb {Z}G} E_{\sigma }^n}$ is acyclic.

Consider the mapping cone ${{\,\textrm{cone}\,}}_{*}(f)$ with basis the union of bases of $C_{*}$ and $C'_{*}$. Since *f* is a homotopy equivalence, ${{\,\textrm{cone}\,}}_{*}(f)$ is contractible and hence its Whitehead torsion $\rho ({{\,\textrm{cone}\,}}_{*}(f))$ is defined. Moreover, ${{\,\textrm{cone}\,}}_{*}(f)\otimes _{\mathbb {Z}G} E_{\sigma }^n$ is also contractible and has (see Remark 3.12)

$$\begin{aligned} \rho _{\sigma \otimes _{\mathbb {Z}}q}({{\,\textrm{cone}\,}}_{*}(f))={{\,\mathrm{\det }\,}}_{E}\left( (\sigma \otimes _{\mathbb {Z}}q)\left( \rho \left( {{\,\textrm{cone}\,}}_{*}(f)\right) \right) ^{-1}\right) . \end{aligned}$$

There is a short exact sequence

$$\begin{aligned} 0\rightarrow C'_{*}\rightarrow {{\,\textrm{cone}\,}}_{*}(f) \rightarrow \Sigma C_{*} \rightarrow 0, \end{aligned}$$

where $\Sigma C_{*}$ is the suspension of $C_{*}$. Since

$$\begin{aligned}{{\,\textrm{cone}\,}}_{*}(f\otimes _{\mathbb {Z}G} \textrm{id}_{E_{\sigma }^n})={{\,\textrm{cone}\,}}_{*}(f)\otimes _{\mathbb {Z}G} E_{\sigma }^n\end{aligned}$$

and

$$\begin{aligned}\Sigma (C_{*}\otimes _{\mathbb {Z}G} E_{\sigma }^n)=\Sigma C_{*} \otimes _{\mathbb {Z}G} E_{\sigma }^n,\end{aligned}$$

the above short exact sequence is still exact after tensoring with $E_{\sigma }^n$. Now, [7, (17.2)] (thinking of these modules as $(D,\{\pm 1\})$-modules in the sense of [7]) yields

$$\begin{aligned}{} & {} \rho _{\sigma \otimes _{\mathbb {Z}}q}(C'_{*})\cdot (\rho _{\sigma \otimes _{\mathbb {Z}}q}(C_{*}))^{-1}\\{} & {} \quad =\rho _{\sigma \otimes _{\mathbb {Z}}q}({{\,\textrm{cone}\,}}_{*}(f))={{\,\mathrm{\det }\,}}_{E}\left( (\sigma \otimes _{\mathbb {Z}}q)\left( \rho \left( {{\,\textrm{cone}\,}}_{*}(f)\right) \right) ^{-1}\right) ,\end{aligned}$$

and thus

$$\begin{aligned} P(\rho _{\sigma \otimes _{\mathbb {Z}}q}(C'_{*}))-P(\rho _{\sigma \otimes _{\mathbb {Z}}q}(C_{*}))=P\left( {{\,\mathrm{\det }\,}}_{E}\left( (\sigma \otimes _{\mathbb {Z}}q)\left( \rho \left( {{\,\textrm{cone}\,}}_{*}(f)\right) \right) ^{-1}\right) \right) . \end{aligned}$$

Since $(\sigma \otimes _{\mathbb {Z}}q)(\rho ({{\,\textrm{cone}\,}}_{*}(f)))$ is a matrix over $DG_{\textrm{fab}}$, $P((\sigma \otimes _{\mathbb {Z}}q)(\rho ({{\,\textrm{cone}\,}}_{*}(f))))$ is a single polytope by Theorem 2.12. Since $\rho ({{\,\textrm{cone}\,}}_{*}(f))$ is invertible over $\mathbb {Z}G$, ${(\sigma \otimes _{\mathbb {Z}}q)((\rho ({{\,\textrm{cone}\,}}_{*}(f)))^{-1})}$ is well defined and is a matrix over $DG_{\textrm{fab}}$. Theorem 2.12 then implies that

$$\begin{aligned}P\left( (\sigma \otimes _{\mathbb {Z}}q)\left( \left( \rho \left( {{\,\textrm{cone}\,}}_{*}(f)\right) \right) ^{-1}\right) \right) \end{aligned}$$

is also a single polytope. We have

$$\begin{aligned}P((\sigma \otimes _{\mathbb {Z}}q)(\rho ({{\,\textrm{cone}\,}}_{*}(f))))+P\left( (\sigma \otimes _{\mathbb {Z}}q)\left( \left( \rho \left( {{\,\textrm{cone}\,}}_{*}(f)\right) \right) ^{-1}\right) \right) =P(\textrm{Id})=0,\end{aligned}$$

and so $P((\sigma \otimes _{\mathbb {Z}}q)(\rho ({{\,\textrm{cone}\,}}_{*}(f)))){=}-P((\sigma \otimes _{\mathbb {Z}}q)((\rho ({{\,\textrm{cone}\,}}_{*}(f)))^{-1}))$. But since both are single polytopes, $P((\sigma \otimes _{\mathbb {Z}}q)(\rho ({{\,\textrm{cone}\,}}_{*}(f)))){=}P((\sigma \otimes _{\mathbb {Z}}q)((\rho ({{\,\textrm{cone}\,}}_{*}(f)))^{-1})) =0$. $\square$

Suppose that *G* is of type *F* and is $(\sigma \otimes _{\mathbb {Z}} q)$-acyclic. Let $C_{*},C'_{*}$ be two finite type based free resolutions of $\mathbb {Z}$ over $\mathbb {Z}G$. The above proposition then implies that $\Vert \cdot \Vert _{\sigma ,C_{*}}=\Vert \cdot \Vert _{\sigma ,C'_{*}}$ and thus the agrarian norm does not depend on the choice of resolution. In this case, we will simply denote $\Vert \cdot \Vert _{\sigma ,C_{*}}$ by $\Vert \cdot \Vert _{\sigma }$ and call it the $\sigma$*-agrarian norm* of *G*.

#### Example 3.17

(Thurston norm) Suppose that *G* is type *F* locally indicable and has vanishing $\ell ^2$-Betti numbers. Let $\tau :\mathbb {C}G\hookrightarrow \mathcal {D}_G$ be the embedding of $\mathbb {C}G$ into the Linnell skew field. Then the *Thurston norm*, denoted by $\Vert \cdot \Vert _T$, is the $\tau$-agrarian norm $\Vert \cdot \Vert _{\tau }$. If *G* is the fundamental group of a connected orientable irreducible 3-manifold $M\ne S^1\times D^2$ with empty or toroidal boundary, then $\Vert \cdot \Vert _T$ is exactly the classical Thurston semi-norm of *M* by combining Theorem 5.1 below and [15, Theorem 0.2]. The corresponding agrarian polytope $P(\rho _{\tau \otimes _{\mathbb {Z}}q})$ is the universal $L^2$-torsion polytope defined in [14, Section 3.2], and $2P(\rho _{\tau \otimes _{\mathbb {Z}}q})$ is the dual Thurston polytope [14, Theorem 3.35].

#### Example 3.18

(Twisted Alexander norm) Suppose that *G* is (type *F*)-by-(infinite cyclic) and $\sigma :G\rightarrow {{\,\textrm{GL}\,}}_n(\mathbb {C})$ is a complex representation. The *twisted Alexander norm with respect to* $\sigma$ is the agrarian norm $\Vert \cdot \Vert _{\sigma }$. If $\sigma$ is the trivial representation $G\rightarrow {{\,\textrm{GL}\,}}_1(\mathbb {C})$ that sends every $g\in G$ to 1, then $\Vert \cdot \Vert _{\sigma }$ is called the *(untwisted) Alexander norm*.

## Twisted $\ell ^2$-Betti numbers

This section is devoted to the proof of the following result.

### Theorem 4.1

Let $F\rightarrow E \rightarrow B$ be a fibration of connected finite CW-complexes, or more generally, topological spaces that are homotopy equivalent to connected finite CW-complexes. Suppose that $\pi _1(B)$ is virtually locally indicable. If *F* is simply connected, or more generally, if the map $\pi _1(E)\rightarrow \pi _1(B)$ induced by the fibration is an isomorphism, then:

(i) For all $i \in {\mathbb {N}}$ we have $b^{(2)}_i(E)\leqslant \sum ^i_{j=0}b_j(F)\cdot b^{(2)}_{i-j}(B)$.
(ii) If the homology of *F* with $\mathbb {C}$-coefficients is non-zero in at most two degrees, 0 and *n* with $n\geqslant \max \{2, \dim B \}$, then for every $i \in {\mathbb {N}}$ we have
  $$\begin{aligned}b^{(2)}_i(E)=b^{(2)}_i(B)+b_n(F)\cdot b^{(2)}_{i-n}(B).\end{aligned}$$
(iii) If *B* is a closed aspherical manifold of odd dimension and satisfies the Singer Conjecture, then for all *i*, we have
  $$\begin{aligned}b^{(2)}_i(E)=0.\end{aligned}$$
(iv) If *B* is a closed aspherical manifold with $\dim B=2n$ that satisfies the Singer Conjecture, then for all *i*, we have
  $$\begin{aligned}b^{(2)}_i(E)=b_{i-n}(F) \cdot b^{(2)}_n(B).\end{aligned}$$
  If in addition *B* is a closed negatively curved Riemannian manifold, then by the Singer Conjecture, $b^{(2)}_n(B)>0$. For every *i* such that $b_i(F)>0$, we have
  $$\begin{aligned}b^{(2)}_{n+i}(E)>0.\end{aligned}$$
  In particular, if *F* is a closed orientable manifold, then
  $$\begin{aligned}b^{(2)}_{n+\dim F}(E)>0.\end{aligned}$$

We prove this theorem by giving an affirmative answer to the following Question 4.2 due to Lück for locally indicable groups.

### Question 4.2

(Lück) Let *G* be a group, $\sigma :G\rightarrow {{\,\textrm{GL}\,}}_n(\mathbb {C})$ a complex representation of *G*, and $C_{*}$ a chain complex of $\mathbb {Z}G$-modules. Is it true that

$$\begin{aligned}b^{(2),\sigma }_i(C_{*})=n\cdot b^{(2)}_i(C_{*})\end{aligned}$$

for all *i*?

Our strategy to answer Question 4.2 is to interpret (twisted) $\ell ^2$-Betti numbers as special cases of agrarian Betti numbers and then extend every complex representation of a locally indicable group *G* to a representation of $\mathcal {D}_G$. The details will be given in Theorems 4.3 and 4.4. For the rest of this section, let *G* be a locally indicable group, $\mathcal {D}_G$ the Linnell skew field of *G*, $\tau :\mathbb {C}G\hookrightarrow \mathcal {D}_G$ the natural inclusion, and $\sigma :G \rightarrow \textrm{GL}_n(\mathbb {C})$ a complex representation. Consider the left *G*-module $\mathcal {D}_{\sigma }^n$, which is the same as $\mathcal {D}_G^n$ as a set, with the left *G*-action given by the tensor product representation $\sigma \otimes _{\mathbb {C}}\tau :G\rightarrow \textrm{GL}_n(\mathcal {D}_G)$ and a right $\mathcal {D}_G$-module structure given by coordinate-wise multiplication. Here, once again the subscript indicates that the left *G*-action on $\mathcal {D}_{\sigma }^n$ is given by $\sigma \otimes _{\mathbb {C}}\tau$. By Example 3.4, the (twisted) $\ell ^2$-Betti numbers are special cases of agrarian Betti numbers:

$$\begin{aligned} b^{(2)}_i(C_{*})&=\dim _{\mathcal {D}_G} H_i(C_{*}\otimes _{\mathbb {Z}G} \mathcal {D}_G)=b^{\tau }_i(C_{*}),\\ b^{(2),\sigma }_i(C_{*})&=\dim _{\mathcal {D}_G} H_i(C_{*}\otimes _{\mathbb {Z}G} \mathcal {D}_{\sigma }^n)=b^{\sigma \otimes _{\mathbb {C}} \tau }_i(C_{*}) \end{aligned}$$

for all *i*. So Lück’s question will be answered if we can relate $b^{\sigma \otimes _{\mathbb {C}} \tau }_i(C_{*})$ to $b^{\tau }_i(C_{*})$.

### Theorem 4.3

Let *G* be a locally indicable group, $\tau :G\rightarrow \mathcal {D}_G$ the natural map of *G* into its Linnell skew field, and $\sigma :G\rightarrow {{\,\textrm{GL}\,}}_n(\mathbb {C})$ a finite-dimensional complex representation. Then $\sigma \otimes _{\mathbb {C}} \tau$ extends to a ring homomorphism $\widetilde{\sigma }:\mathcal {D}_G\rightarrow M_n(\mathcal {D}_G)$.

### Proof

For all $H\leqslant G$, let ${\widetilde{\mathcal {D}}}_H$ be the division closure of $(\sigma \otimes _{\mathbb {C}}\tau )(\mathbb {C}H)$ in $M_n(\mathcal {D}_G)$. Let ${{\,\textrm{Rat}\,}}(\mathbb {C}^{\times }G)$ be the universal rational $\mathbb {C}^{\times }G$-semiring. Section 2.14 gives us a map

$$\begin{aligned}\Phi :{{\,\textrm{Rat}\,}}(\mathbb {C}^{\times }G)\cup \{0\}\rightarrow {\widetilde{\mathcal {D}}}_G.\end{aligned}$$

By Remark 2.31 and Lemma 2.32, $\Phi ({{\,\textrm{Rat}\,}}(\mathbb {C}^{\times }H)\cup \{0\})={\widetilde{\mathcal {D}}}_H$, where we think of ${{\,\textrm{Rat}\,}}(\mathbb {C}^{\times }H)\cup \{0\}$ as a subset of ${{\,\textrm{Rat}\,}}(\mathbb {C}^{\times }G)\cup \{0\}$.

Let $\mathcal {T}$ be the set of finite rooted trees. Section 2.14 gives us the notion of *G*-complexity ${{\,\textrm{Tree}\,}}_G(x)\in \mathcal {T}$ of $x\in {\widetilde{\mathcal {D}}}_G$. We first prove that ${\widetilde{\mathcal {D}}}_G$ is a skew field by inducting on the *G*-complexity. Our proof uses the idea of the proof of [26, Theorem 6.1].

Consider a non-zero element $x\in {\widetilde{\mathcal {D}}}_G$. If ${{\,\textrm{Tree}\,}}_G(x)=1_{\mathcal {T}}$, then $x\in (\sigma \otimes _{\mathbb {C}}\tau )(\mathbb {C}^{\times }G)$ is invertible. Now assume that ${{\,\textrm{Tree}\,}}_G(x)>1_{\mathcal {T}}$ and that for all $0\ne y\in {\widetilde{\mathcal {D}}}_G$ with ${{\,\textrm{Tree}\,}}_G(y)<{{\,\textrm{Tree}\,}}_G(x)$, *y* is invertible in ${\widetilde{\mathcal {D}}}_G$. Take $\alpha \in {{\,\textrm{Rat}\,}}(\mathbb {C}^{\times }G)$ realizing the *G*-complexity of *x*. By Theorem 2.30 (i) we may assume that $\alpha$ is primitive because multiplying by an element in $\mathbb {C}^{\times }G$ does not change the complexity nor the conclusion about the invertibility of *x*. Set *H* to be the image of $\textrm{source}(\alpha )$ under the homomorphism $\mathbb {C}^{\times }G\rightarrow \mathbb {C}^{\times }G/\mathbb {C}^{\times }=G$. Then $\alpha \in {{\,\textrm{Rat}\,}}(\mathbb {C}^{\times }H)$ and so $x\in \Phi ({{\,\textrm{Rat}\,}}(\mathbb {C}^{\times }H))={\widetilde{\mathcal {D}}}_H$. If $H=\{1\}$, then ${\widetilde{\mathcal {D}}}_H=\mathbb {C}\cdot \textrm{Id}$ and since $x\ne 0$, it is invertible. If $H\ne \{1\}$, then by Theorem 2.30 (i), *H* is finitely generated, and thus there exists a normal subgroup $N\lhd H$ and an element $t\in H$ of infinite order such that $H=N\rtimes \langle t \rangle$.

Consider the *H*-complexity ${{\,\textrm{Tree}\,}}_H$ given by Sect. 2.14. Note that for all $0\ne y\in {\widetilde{\mathcal {D}}}_H$ with ${{\,\textrm{Tree}\,}}_H(y)<{{\,\textrm{Tree}\,}}_H(x)$, we have

4 $$\begin{aligned} {{\,\textrm{Tree}\,}}_G(y)\leqslant {{\,\textrm{Tree}\,}}_H(y)<{{\,\textrm{Tree}\,}}_H(x)={{\,\textrm{Tree}\,}}_G(x). \end{aligned}$$

Indeed, by definition we have ${{\,\textrm{Tree}\,}}_G(y)\leqslant {{\,\textrm{Tree}\,}}_H(y)$. Note that ${{\,\textrm{Tree}\,}}_G(x)={{\,\textrm{Tree}\,}}(\alpha )$, where the latter is computed by thinking of $\alpha$ as an element of ${{\,\textrm{Rat}\,}}(\mathbb {C}^{\times }G)$. Note also that ${{\,\textrm{Tree}\,}}_H(x)\leqslant {{\,\textrm{Tree}\,}}(\alpha )$, where the latter is computed by thinking of $\alpha$ as an element of ${{\,\textrm{Rat}\,}}(\mathbb {C}^{\times }H)$. As pointed out by Remark 2.31, the two ways of computing ${{\,\textrm{Tree}\,}}(\alpha )$ yield the same answer. Thus, we also have ${{\,\textrm{Tree}\,}}_H(x)\leqslant {{\,\textrm{Tree}\,}}_G(x)$. The induction hypothesis together with (4) then says that *y* is invertible in ${\widetilde{\mathcal {D}}}_G$.

For simplicity, denote $\tau (t)$ by *t* and $\sigma (t)\cdot \tau (t)$ by *s*. Let

$$\begin{aligned}\mathcal {D}_N(\!(t)\!), \quad {\widetilde{\mathcal {D}}}_N(\!(s)\!), \quad M_n(\mathcal {D}_N)(\!(s)\!)\end{aligned}$$

be the twisted Laurent power series rings given by Sect. 2.14. By Proposition 2.33 we have $x\in {\widetilde{\mathcal {D}}}_N(\!(s)\!)$. So *x* can be written as a Laurent power series $x=\sum _i x_is^i$ with $x_i\in {\widetilde{\mathcal {D}}}_N$. We claim that there are at least two non-zero summands in $\sum _i x_is^i$. Otherwise, we would have $\alpha \in {{\,\textrm{Rat}\,}}(\mathbb {C}^{\times }N)t^i$ for some *i*, and so $\textrm{source}(\alpha )\subset \mathbb {C}^{\times }N$, and hence $H\leqslant N$, a contradiction.

Thus, Proposition 2.33 implies that ${{\,\textrm{Tree}\,}}_H(x_i)<{{\,\textrm{Tree}\,}}_H(x)$ for all *i*. Inequality (4) implies that

$$\begin{aligned}{{\,\textrm{Tree}\,}}_G(x_i)<{{\,\textrm{Tree}\,}}_G(x).\end{aligned}$$

Thus, the induction hypothesis implies that if $x_i\ne 0$ then it is invertible in ${\widetilde{\mathcal {D}}}_G$, and thus in ${\widetilde{\mathcal {D}}}_N$ and $M_n(\mathcal {D}_N)$, by (3). So *x* is invertible in $M_n(\mathcal {D}_N)(\!(s)\!)=M_n(\mathcal {D}_N(\!(t)\!))$, by Remark 2.5. Note that *x* belongs to the subring $M_n(\mathcal {D}_H)$. So *x* is invertible in $M_n(\mathcal {D}_H)$, and thus in $M_n(\mathcal {D}_G)$. So *x* is invertible in ${\widetilde{\mathcal {D}}}_G$. Therefore, ${\widetilde{\mathcal {D}}}_G$ is a skew field.

We will now show that ${\widetilde{\mathcal {D}}}_G$ is a Hughes-free $\mathbb {C}G$-field. Let $H'\leqslant G$ be any non-trivial finitely generated subgroup and suppose $H'=N'\rtimes \langle t' \rangle$ for some normal subgroup $N'\lhd H'$ and an infinite-order element $t'\in H'$. Since $\mathcal {D}_G$ is a Hughes-free $\mathbb {C}G$-field, by considering every entry of the matrices, we see that the sum

$$\begin{aligned}M_n(\mathcal {D}_{N'})+M_n(\mathcal {D}_{N'})\cdot \tau (t')+\cdots +M_n(\mathcal {D}_{N'})\cdot \tau (t')^f\end{aligned}$$

is direct for every $f\in \mathbb {N}^+$. The containment (3) then implies that the sum

$$\begin{aligned}{\widetilde{\mathcal {D}}}_{N'}+{\widetilde{\mathcal {D}}}_{N'}\cdot \sigma (t')\tau (t')+\cdots +{\widetilde{\mathcal {D}}}_{N'}\cdot \sigma (t')^f\tau (t')^f\end{aligned}$$

is also direct, and thus ${\widetilde{\mathcal {D}}}_G$ is a Hughes-free $\mathbb {C}G$-field. The main result of [23] then implies that there exists a ring homomorphism $\widetilde{\sigma }:\mathcal {D}_G\rightarrow M_n(\mathcal {D}_G)$ that extends $\sigma \otimes _{\mathbb {C}}\tau$. $\square$

Consider the $\mathcal {D}_G$-$\mathcal {D}_G$-bimodule *M*, that is the same as $\mathcal {D}_G^n$ as a set, with the left $\mathcal {D}_G$-module structure given by $c\bullet v=\widetilde{\sigma }(c)\cdot v$ for all $c\in \mathcal {D}_G,v\in M$, and the right $\mathcal {D}_G$-module structure given by coordinate-wise multiplication. Given any right $\mathcal {D}_G$-module *U*, the action $\bullet$ then induces a tensor product $U\otimes _{\mathcal {D}_G} M$, $(u\cdot c)\otimes v=u\otimes (c\bullet v)=u\otimes (\widetilde{\sigma }(c)\cdot v)$ for all $u\in U,c\in \mathcal {D}_G,v\in M$. Note that $U\otimes _{\mathcal {D}_G}M\cong M^{\dim _{\mathcal {D}_G}U}$ as a right $\mathcal {D}_G$-module and thus

5 $$\begin{aligned} \dim _{\mathcal {D}_G} U\otimes _{\mathcal {D}_G} M = n\cdot \dim _{\mathcal {D}_G} U. \end{aligned}$$

Here we adopt the convention $0\cdot \infty =0$ and $n\cdot \infty =\infty$ for all $n>0$.

### Theorem 4.4

Let *G* be a locally indicable group, let $C_{*}$ be a $\mathbb {Z}G$-chain complex, and let $\sigma :G\rightarrow {{\,\textrm{GL}\,}}_n(\mathbb {C})$ be a linear representation of *G*. Then for all *i* we have

$$\begin{aligned}b^{(2),\sigma }_i(C_{*})=n\cdot b^{(2)}_i(C_{*}).\end{aligned}$$

### Proof

Identify

$$\begin{aligned}C_{*}\otimes _{\mathbb {Z}G}\mathcal {D}_{\sigma }^n\cong C_{*}\otimes _{\mathbb {Z}G} \mathcal {D}_G \otimes _{\mathcal {D}_G} M.\end{aligned}$$

As a left-module over the division ring $\mathcal {D}_G$ (with the action given by $\bullet$), *M* is free and thus for all *i*

$$\begin{aligned}H_i(C_{*}\otimes _{\mathbb {Z}G} \mathcal {D}_G \otimes _{\mathcal {D}_G} M)\cong H_i(C_{*}\otimes _{\mathbb {Z}G} \mathcal {D}_G)\otimes _{\mathcal {D}_G} M.\end{aligned}$$

The desired result then follows from (5). $\square$

Question 4.2 arises naturally in the process of computing $\ell ^2$-Betti numbers of fibrations. The following argument can be easily extracted from the proof of [39, Lemma 5.4]. We reproduce it here for the convenience of the reader. Let $F\rightarrow E\rightarrow B$ be a fibration of connected finite CW-complexes, or more generally, topological spaces that are homotopy equivalent to connected finite CW-complexes, such that $\pi _1(B)$ is locally indicable and the induced homomorphism $\pi _1(E)\rightarrow \pi _1(B)$ is bijective (e.g., when *F* is simply connected). The Leray–Serre spectral sequence then yields

6 $$\begin{aligned} \small E^2_{p,q}{} & {} =H_p(C_{*}({\widetilde{B}})\otimes _{\mathbb {Z}[{\pi _1(B)}]}(H_q(F,\mathbb {C})\otimes _{\mathbb {C}} \mathcal {D}_{\pi _1(B)}))\nonumber \\{} & {} \Rightarrow H_{p+q}(C_{*}({\widetilde{E}})\otimes _{\mathbb {Z}[{\pi _1(E)}]}\mathcal {D}_{\pi _1(B)}), \end{aligned}$$

where $\mathbb {Z}\pi _1(B)$ acts on $H_q(F,\mathbb {C})\otimes _{\mathbb {C}} \mathcal {D}_{\pi _1(B)}$ by the diagonal action and $\mathbb {Z}\pi _1(E)$ acts on $\mathcal {D}_{\pi _1(B)}$ via the induced isomorphism $\pi _1(E)\cong \pi _1(B)$. Thus, $E^2_{p,q}$ is the $\ell ^2$-homology of *B* twisted by the representation $\eta :B\rightarrow \textrm{GL}(H_q(F,\mathbb {C}))$. Theorem 4.4 then implies

7 $$\begin{aligned} \dim _{\mathcal {D}_{\pi _1(B)}}E^2_{p,q}=b^{(2),\eta }_p(B)=b_q(F)\cdot b^{(2)}_p(B). \end{aligned}$$

Below, we prove Theorem 4.1, and Corollaries 1.2 and 1.3.

### Proof of Theorem 4.1

Suppose first that $\pi _1(B)$ is locally indicable. Since $\mathcal {D}_{\pi _1(B)}$ is a skew field, the spectral sequence (6) implies that $H_n(C_{*}({\widetilde{E}})\otimes _{\mathbb {Z}[{\pi _1(E)}]}\mathcal {D}_{\pi _1(B)})$ is a a direct sum of subquotients of $E^2_{i,n-i}$ for $i=0,1,\cdots ,n$. Together with (7), this implies (i).

Suppose that the assumption of (ii) holds. Then Theorem 4.4 implies that the spectral sequence (6) stabilizes at the $E^2$-page with

$$\begin{aligned} \dim _{\mathcal {D}_{\pi _1(B)}} E^2_{p,q}= {\left\{ \begin{array}{ll} b^{(2)}_p(B),&{}\text {if }q=0\\ b_n(F)\cdot b^{(2)}_p(B),&{}\text {if }q=n\\ 0,&{}\text {otherwise.} \end{array}\right. } \end{aligned}$$

Item (ii) follows from a computation using the spectral sequence (6).

Suppose that the assumption of (iii) holds. Then the Singer Conjecture implies that $b^{(2)}_{*}(B)=0$. Theorem 4.4 implies that the $E^2$-page of the spectral sequence (6) is 0, from which (iii) follows.

Suppose that the assumption of (iv) holds. Then Theorem 4.4 implies that the spectral sequence (6) stabilizes at the $E^2$-page with

$$\begin{aligned} \dim _{\mathcal {D}_{\pi _1(B)}} E^2_{p,q}= {\left\{ \begin{array}{ll} b_q(F)\cdot b^{(2)}_n(B), &{}\text {if } p=n\\ 0, &{}\text {otherwise.} \end{array}\right. } \end{aligned}$$

Item (iv) follows from computation using the spectral sequence (6). This finishes the proof for the special case where $\pi _1(B)$ is locally indicable.

Let us consider the general case where $\pi _1(B)\cong \pi _1(E)$ are virtually locally indicable. Let ${\widehat{B}}$ be a *d*-sheeted cover of *B* for some *d* such that $\pi _1({\widehat{B}})$ is locally indicable, and let ${\widehat{E}}$ be the pullback of $E\rightarrow B$ along ${\widehat{B}}\rightarrow B$. Then we have a fibration

$$\begin{aligned}F\rightarrow {\widehat{E}} \rightarrow {\widehat{B}}\end{aligned}$$

with $\pi _1({\widehat{E}})=\pi _1({\widehat{B}})$ locally indicable.

Note that $\dim B\leqslant n$ if and only if $\dim {\widehat{B}}\leqslant n$, *B* is a closed aspherical manifold if and only if so is ${\widehat{B}}$, and ${\widehat{B}}$ is a Riemannian manifold with negative sectional curvature as so is *B*. By the above, items (i), (ii), (iii), (iv) hold with ${\widehat{E}}$ in place of *E* and ${\widehat{B}}$ in place of *B*. By [38, Theorem 1.35 (9)], we have for all *i*

$$\begin{aligned}b^{(2)}_i({\widehat{E}})=d\cdot b^{(2)}_i(E),~~b^{(2)}_i({\widehat{B}})=d\cdot b^{(2)}_i(B),\end{aligned}$$

which finishes the proof. $\square$

### Proof of Corollary 1.2

In this case, the spectral sequence (6) has only one non-zero column, and thus stabilizes. By Example 2.17, $\pi _1(B)$ is locally indicable. Thus, the desired result follows from Theorem 4.4 and

$$\begin{aligned} b^{(2)}_i(B)= {\left\{ \begin{array}{ll} -\chi (B),&{}\text {if }i=1\\ 0,&{}\text {otherwise.} \end{array}\right. } \end{aligned}$$

$$\square $$

### Proof of Corollary 1.3

By Proposition 2.20, $\pi _1(B)$ is virtually locally indicable. Thus, the corollary follows from Theorem 4.1 (i) and the computation of the $\ell ^2$-Betti number of 3-manifolds [37, Theorem 0.1]. $\square$

### Remark 4.5

Combining Theorem 4.1, Proposition 2.20 and [37, Theorem 0.1], one can obtain a general version of Corollary 1.3 for all compact connected orientable 3-manifolds *B* with empty or toroidal boundary. Instead of having $b^{(2)}_{*}(E)=0$ we will have that $b^{(2)}_{*}(E)$ can be computed by a homological spectral sequence that stabilizes at the $E^2$-page. We leave the precise statement to the reader.

## Agrarian norm and Euler characteristic

If *M* is a closed connected orientable irreducible 3-manifold and $\phi \in H^1(M,\mathbb {Z})$ is a character induced by a fibration $F\rightarrow M\rightarrow S^1$ of *M* over the circle $S^1$, then $\Vert \phi \Vert _T=-\chi (F)$, where $\Vert \phi \Vert _T$ is the Thurston norm of $\phi$ [48]. The goal of the current section is Theorem 5.1 below, which generalizes the above result of [48]. In Sects. 6 and 7, we will apply Theorem 5.1 to deduce the equality between the twisted Alexander and Thurston norms for fibered characters.

### Theorem 5.1

Let *G* be a type *F* group, $\sigma :G\rightarrow {{\,\textrm{GL}\,}}_n(D)$ a representation over a skew field *D*, $\phi \in H^1(G,\mathbb {Z})$ be a primitive character and $q:G\rightarrow G_{\textrm{fab}}$ the natural quotient map from *G* onto its maximal free abelian quotient $G_{\textrm{fab}}$. If $b_{*}^{\sigma \otimes _{\mathbb {Z}}q}(G)=0$, then $\Vert \phi \Vert _{\sigma }$ and $\chi ^{\sigma \otimes _{\mathbb {Z}} q}(\ker \phi )$ are well defined and

$$\begin{aligned}\Vert \phi \Vert _{\sigma }=-\chi ^{\sigma \otimes _{\mathbb {Z}} q}(\ker \phi ).\end{aligned}$$

Below, we use the notation of the above theorem. Let $H=\ker \phi$, let $t\in G$ such that $\phi (t)=1$, let ${\bar{t}}=q(t)$, let *X* be a basis of $G_{\textrm{fab}}$ such that ${\bar{t}}\in X$ and $\phi (x)=0$ for all $x\in X\smallsetminus \{{\bar{t}}\}$, let *BG* be a finite *K*(*G*, 1) CW-complex, let *EG* be the universal cover of *BG*, let ${\bar{q}}:G\twoheadrightarrow G_{\textrm{fab}}/\langle {\bar{t}}\rangle$ the natural quotient map, and let $Y=X\smallsetminus \{{\bar{t}}\}$. To emphasize the role played by *X* and *Y*, we denote ${{\,\textrm{Ore}\,}}(DG_{\textrm{fab}})$ by *D*(*X*) and ${{\,\textrm{Ore}\,}}(DL)$ by *D*(*Y*), where *L* is the subgroup of $G_{\textrm{fab}}$ generated by *Y*. We write $(D(Y)[{\bar{t}}^{\pm }])_{\sigma }^n$ for the $\mathbb {Z}G$-$D(Y)[{\bar{t}}^{\pm }]$-bimodule that equals $(D(Y)[{\bar{t}}^{\pm }])^n$ as a set, with the left action of *G* induced by $\sigma$ and the right action given by coordinate-wise multiplication. And we write $(D(Y))_{\sigma }^n$ for the $\mathbb {Z}H$-*D*(*Y*)-bimodule defined in the same manner as in Convention 3.3.

As *D*(*Y*) is a sub-skew field of *D*(*x*), *D*(*X*) is flat over *D*(*Y*). It follows that for all *k*, we have

$$\begin{aligned}\dim _{D(X)}H_k(C_{*}(EG)\otimes _{\mathbb {Z}H} (D(X))_{\sigma }^n)=\dim _{D(Y)}H_k(C_{*}(EG)\otimes _{\mathbb {Z}H} (D(Y))_{\sigma }^n),\end{aligned}$$

where $(C_{*}(EG),\partial ^{EG}_{*})$ is the cellular chain complex of *EG* and the right-hand side tensor product is taken with respect to the representation $\sigma \otimes _{\mathbb {Z}} {\bar{q}}$. Therefore, $\chi ^{\sigma \otimes _{\mathbb {Z}} q}(H)$ is well defined if and only if so is $\chi ^{\sigma \otimes _{\mathbb {Z}} {\bar{q}}}(H)$, and if they are both well defined,

8 $$\begin{aligned} \chi ^{\sigma \otimes _{\mathbb {Z}} q}(H)=\chi ^{\sigma \otimes _{\mathbb {Z}} {\bar{q}}}(H). \end{aligned}$$

Note that there is an isomorphism of chain complexes of *D*(*Y*)-modules

9 $$\begin{aligned} C_{*}(EG)\otimes _{\mathbb {Z}H} (D(Y))_{\sigma }^n \xrightarrow []{\cong } C_{*}(EG)\otimes _{\mathbb {Z}G} (D(Y)[{\bar{t}}^{\pm }])_{\sigma }^n, \end{aligned}$$

which maps $e\otimes d$ to $e\otimes d$ for all $e\in C_{*}(EG)$ and $d\in (D(Y))_{\sigma }^n$. The inverse of this map sends $e\otimes d{\bar{t}}^k$ to $(et^k)\otimes (\sigma (t))^{-k}(d)$. Note also the following isomorphism of chain complexes of *D*(*X*)-modules

10 $$\begin{aligned} C_{*}(EG)\otimes _{\mathbb {Z}G} (D(Y)[{\bar{t}}^{\pm }])_{\sigma }^n\otimes _{D(Y)[{\bar{t}}^{\pm }]}D(X)\cong C_{*}(EG)\otimes _{\mathbb {Z}G} (D(X))_{\sigma }^n. \end{aligned}$$

For simplicity, let

$$\begin{aligned}(C_{*},\partial _{*})=(C_{*}(EG)\otimes _{\mathbb {Z}G} (D(Y)[{\bar{t}}^{\pm }])_{\sigma }^n,\partial ^{EG}_{*}\otimes _{\mathbb {Z}G} \textrm{id}_{(D(Y)[{\bar{t}}^{\pm }])_{\sigma }^n}).\end{aligned}$$

For $k\in \mathbb {N}$, let $\mathcal {B}^{EG}_k$ be a $\mathbb {Z}G$-basis of $C_k(EG)$ consisting of cells of dimension *k*, and let *V* be the standard $D(Y)[{\bar{t}}^\pm ]$-basis of $(D(Y)[{\bar{t}}^{\pm }])_{\sigma }^n$. Then

$$\begin{aligned}\mathcal {B}_k=\{\Delta \otimes v\mid \Delta \in \mathcal {B}^{EG}_k,v\in V\}\subset C_{*}\end{aligned}$$

is a $D(Y)[{\bar{t}}^{\pm }]$-basis for $C_{*}$. By definition, $\Vert \phi \Vert _{\sigma }$ is computed using the basis $\mathcal {B}_{*}$. But in order to prove the theorem we will use another basis that is equivalent to $\mathcal {B}_{*}$.

### Lemma 5.2

There are two families of subsets of $C_{*}$, $\{\mathcal {B}'_k\}^{\infty }_{k=0}$ and $\{\mathcal {B}''_k\}^{\infty }_{k=0}$, such that the following hold for every *k*.

(i) $\mathcal {B}'_k\cup \mathcal {B}''_k$ is a basis of $C_k$ and the change of basis matrix *M* from $\mathcal {B}_k$ to $\mathcal {B}'_k\cup \mathcal {B}''_k$ satisfies ${{\,\mathrm{\det }\,}}_{D(X)}(M)=\pm 1$, where we think of *M* as a matrix over *D*(*X*) to take the determinant.
(ii) Denote by $\textrm{span}_{D(Y)[{\bar{t}}^{\pm }]}$ the linear span over $D(Y)[{\bar{t}}^{\pm }]$. Then
  $$\begin{aligned} \partial _k(\textrm{span}_{D(Y)[{\bar{t}}^{\pm }]}(\mathcal {B}'_k))&\subset \textrm{span}_{D(Y)[{\bar{t}}^{\pm }]}(\mathcal {B}''_{k-1}),\\ \textrm{span}_{D(Y)[{\bar{t}}^{\pm }]}(\mathcal {B}''_k)&=\ker \partial _k. \end{aligned}$$
(iii) Let ${\overline{\partial }}_k:\textrm{span}_{D(Y)[{\bar{t}}^{\pm }]}(\mathcal {B}'_k)\rightarrow \textrm{span}_{D(Y)[{\bar{t}}^{\pm }]}(\mathcal {B}''_{k-1})$ be the restriction of $\partial _k$. Then the matrix representative of ${\overline{\partial }}_k$ under the bases $\mathcal {B}'_k$ and $\mathcal {B}''_{k-1}$, denoted $[{\overline{\partial }}_k]$, is a diagonal matrix over $D(Y)[{\bar{t}}^{\pm }]$ with non-zero diagonal entries.

### Proof

We prove the lemma by an induction on *k*. First note that (i) through (iii) hold for $k=0$ with $\mathcal {B}'_0=\emptyset ,\mathcal {B}''_0=\mathcal {B}_0$. Now suppose that we have found $\{\mathcal {B}'_k\}^K_{k=0}$ and $\{\mathcal {B}''_k\}^K_{k=0}$ that satisfy (i) through (iii) for $k\leqslant K$. Let $M_{K+1}$ be the matrix representative of $\partial _{k+1}$ under the bases $\mathcal {B}_{K+1}$ and $\mathcal {B}'_K\cup \mathcal {B}''_K$.

The Laurent polynomial ring $D(Y)[{\bar{t}}^{\pm }]$ is a principal ideal domain. By the Euclidean algorithm, we can multiply $M_{K+1}$ on the left and right by elementary matrices over $D(Y)[{\bar{t}}^{\pm }]$ whose diagonal entries are $\pm 1$ to turn $M_{K+1}$ into a diagonal matrix $N_{K+1}$ over $D(Y)[{\bar{t}}^{\pm }]$. The left (resp. right) multiplication of elementary matrices corresponds to the change of the basis $\mathcal {B}'_K\cup \mathcal {B}''_K$ (resp. $\mathcal {B}_{K+1}$). Since non-zero elements of $\textrm{span}_{D(Y)[{\bar{t}}^{\pm }]}(\mathcal {B}'_K)$ have non-zero boundaries, we have $\partial _{K+1}(C_{K+1})\subset \textrm{span}_{D(Y)[{\bar{t}}^{\pm }]}(\mathcal {B}''_K)$. Therefore, we may assume that the change of basis process leaves $\mathcal {B}'_K$ invariant and turns $\mathcal {B}''_K$ into another basis of $\textrm{span}_{D(Y)[{\bar{t}}^{\pm }]}(\mathcal {B}''_K)$. Since $\partial _K(\textrm{span}_{D(Y)[{\bar{t}}^{\pm }]}(\mathcal {B}''_K))=0$, such a change of basis process will not change $[{\overline{\partial }}_K]$. Thus, we can modify $\mathcal {B}''_K$ while still have (i) through (iii) hold for $k\leqslant K$.

Let $\mathcal {B}'_{K+1}$ (resp. $\mathcal {B}''_{K+1}$) be the part of the new basis of $C_{K+1}$ corresponding to the non-zero (resp. zero) diagonal entries of $N_{K+1}$. Then (i) and (ii) follow immediately. Item (iii) is equivalent to $|\mathcal {B}'_{K+1}|=|\mathcal {B}''_K|$. Consider

$$\begin{aligned}C_{K+1}\otimes _{D(Y)[{\bar{t}}^{\pm }]}D(X)\xrightarrow []{\partial _{K+1}\otimes _{D(Y)[{\bar{t}}^{\pm }]} \textrm{id}_{D(X)}}C_K\otimes _{D(Y)[{\bar{t}}^{\pm }]}D(X).\end{aligned}$$

And consider the subsets

$$\begin{aligned} \mathcal {B}'_{K+1}\otimes \{1\}, \mathcal {B}''_{K+1}\otimes \{1\}&\subset C_{K+1}\otimes _{D(Y)[{\bar{t}}^{\pm }]}D(X),\\ \mathcal {B}_K\otimes \{1\}, \mathcal {B}''_K\otimes \{1\}&\subset C_K\otimes _{D(Y)[{\bar{t}}^{\pm }]}D(X). \end{aligned}$$

Since *D*(*X*) is flat over $D(Y)[{\bar{t}}^{\pm }]$, $(\mathcal {B}'_{K+1}\cup \mathcal {B}''_{K+1})\otimes \{1\}$ (resp. $(\mathcal {B}'_K\cup \mathcal {B}''_K)\otimes \{1\}$) is a *D*(*X*)-basis of $C_{K+1}\otimes _{D(Y)[{\bar{t}}^{\pm }]}D(X)$ (resp. $C_K\otimes _{D(Y)[{\bar{t}}^{\pm }]}D(X)$).

Let *N* be the matrix representative of $\partial _{K+1}\otimes _{D(Y)[{\bar{t}}^{\pm }]} \textrm{id}_{D(X)}$ under the bases $(\mathcal {B}'_{K+1}\cup \mathcal {B}''_{K+1})\otimes \{1\}$ and $(\mathcal {B}'_K\cup \mathcal {B}''_K)\otimes \{1\}$. Then *N* is the $(|\mathcal {B}'_{K+1}|+|\mathcal {B}''_{K+1}|)\times (|\mathcal {B}'_K|+|\mathcal {B}''_K|)$-matrix with $[{\overline{\partial }}_{K+1}]$ at the top left corner and 0 elsewhere. In particular,

$$\begin{aligned}|\mathcal {B}'_{K+1}|={{\,\textrm{rk}\,}}_{D(X)} (\partial _{K+1}\otimes _{D(Y)[{\bar{t}}^{\pm }]} \textrm{id}_{D(X)}),\end{aligned}$$

where ${{\,\textrm{rk}\,}}_{D(X)}$ denotes the rank of the image. Similarly,

$$\begin{aligned}|\mathcal {B}'_K|={{\,\textrm{rk}\,}}_{D(X)} (\partial _K\otimes _{D(Y)[{\bar{t}}^{\pm }]} \textrm{id}_{D(X)}),\end{aligned}$$

and thus

$$\begin{aligned}|\mathcal {B}''_K|=\dim _{D(X)}\ker (\partial _K\otimes _{D(Y)[{\bar{t}}^{\pm }]} \textrm{id}_{D(X)}).\end{aligned}$$

As $b^{\sigma \otimes _{\mathbb {Z}}q}(G)=0$, $C_{*}\otimes _{D(Y)[{\bar{t}}^{\pm }]} (D(X))_{\sigma }^n$ is acyclic. Combining with equation (10) this yields

$$\begin{aligned}{{\,\textrm{rk}\,}}_{D(X)}(\partial _{K+1}\otimes _{D(Y)[{\bar{t}}^{\pm }]} \textrm{id}_{D(X)})=\dim _{D(X)}\ker (\partial _K\otimes _{D(Y)[{\bar{t}}^{\pm }]} \textrm{id}_{D(X)}),\end{aligned}$$

from which (iii) follows. $\square$

### Proof of Theorem 5.1

Fix $k\in \mathbb {N}$. Let $e_1,\cdots , e_{\ell _k}$ be the elements of $\mathcal {B}''_k$ and let $f_1,\cdots , f_{\ell _k}$ be the diagonal entries of $[{\overline{\partial }}_{k+1}]$. For $i=1,2,\cdots , \ell _k$, let

$$\begin{aligned}S_{k,i}=\{e_i\cdot {\bar{t}}^j \mid j=0,1,\cdots , (\deg _{{\bar{t}}}f_i)-1\},\end{aligned}$$

where we use the notion of degree introduced in Sect. 2.4. Let

$$\begin{aligned}S_k=\bigcup ^{\ell _k}_{i=1}S_{k,i}\subset C_k.\end{aligned}$$

There is a *D*(*Y*)-module homomorphism from $\textrm{span}_{D(Y)[{\bar{t}}^{\pm }]}(\mathcal {B}''_k)$ to $\textrm{span}_{D(Y)}(S_k)$ that sends each $e_i\cdot {\bar{t}}^j$ in $\textrm{span}_{D(Y)[{\bar{t}}^{\pm }]}(\mathcal {B}''_k)$ to the $e_i\cdot {\bar{t}}^j$ in $\textrm{span}_{D(Y)}(S_k)$ for $j=0,1,\cdots , (\deg _{{\bar{t}}}f_i)-1$. This homomorphism induces a *D*(*Y*)-module isomorphism

$$\begin{aligned}\textrm{span}_{D(Y)[{\bar{t}}^{\pm }]}(\mathcal {B}''_k)/{{\,\textrm{im}\,}}{\overline{\partial }}_{k+1}\cong \textrm{span}_{D(Y)}(S_k).\end{aligned}$$

By (9),

$$\begin{aligned}b^{\sigma \otimes _{\mathbb {Z}}{\bar{q}}}_k(H)=\dim _{D(Y)}(\textrm{span}_{D(Y)[{\bar{t}}^{\pm }]}(\mathcal {B}''_k)/{{\,\textrm{im}\,}}{\overline{\partial }}_{k+1})=\deg _{{\bar{t}}}{{\,\mathrm{\det }\,}}_{D(X)} [{\overline{\partial }}_{k+1}],\end{aligned}$$

where we think of $[\partial _{k+1}]$ as a matrix over *D*(*X*) in order to take the determinant. It then follows from (8) that

11 $$\begin{aligned} \chi ^{\sigma \otimes _{\mathbb {Z}} q}(H)=\sum ^{\infty }_{k=0}(-1)^k \deg _{{\bar{t}}}{{\,\mathrm{\det }\,}}_{D(X)} [\partial _{k+1}]. \end{aligned}$$

On the other hand, by Lemma 5.2 (i) and (iii), $C_{*}$ decomposes as a direct sum of chain complexes of the form

$$\begin{aligned}0\rightarrow \textrm{span}_{D(Y)[{\bar{t}}^{\pm }]}(\mathcal {B}'_k)\xrightarrow []{[{\overline{\partial }}_k]}\textrm{span}_{D(Y)[{\bar{t}}^{\pm }]}(\mathcal {B}''_{k-1})\rightarrow 0.\end{aligned}$$

By tensoring with *D*(*X*) we see that $C_{*}(EG)\otimes _{\mathbb {Z}G} (D(X))_{\sigma }^n$ decomposes as the direct sum of chain complexes of the form

$$\begin{aligned}0\rightarrow \textrm{span}_{D(X)}(\mathcal {B}'_k\otimes \{1\})\xrightarrow []{[{\overline{\partial }}_k]}\textrm{span}_{D(X)}(\mathcal {B}''_{k-1}\otimes \{1\})\rightarrow 0,\end{aligned}$$

where we think of $[\partial _k]$ as a matrix over *D*(*X*).

By Lemma 5.2 (i) and Remark 3.11,

12 $$\begin{aligned} \Vert \phi \Vert _{\sigma }=\sum ^{\infty }_{k=0}(-1)^k\deg _{{\bar{t}}}{{\,\mathrm{\det }\,}}_{D(X)} [\partial _k]. \end{aligned}$$

The desired result follows from equations (11) and (12). $\square$

## Aspherical groups

In this section, we prove the semi-norm property of the agrarian norm and the inequality between the twisted Alexander and Thurston norms for certain aspherical groups. Let *G* be a finitely presentated group and let $q:G\rightarrow G_{\textrm{fab}}$ be the natural homomorphism of *G* onto its maximal free abelian quotient $G_{\textrm{fab}}$. We start with a method to modify a given finite group presentation.

### Lemma 6.1

Let *G* be a group given by a finite presentation

13 $$\begin{aligned} G=\langle X\mid \mathcal {R}\rangle . \end{aligned}$$

Then there exists a finite presentation

14 $$\begin{aligned} G=\langle Y\mid \mathcal {S}\rangle . \end{aligned}$$

such that the tuple $\left( q(y): y \in Y, q(y) \ne 0 \right)$ is a basis for $G_{\textrm{fab}}$ and the presentation complexes of (13) and (14) are homotopy equivalent.

Moreover, if $\phi \in H^1(G,\mathbb {Z})$ is a primitive integral character, then we can further guarantee that there exists $y\in Y$ such that $\phi (y)=1$ and $\phi (y')=0$ for all $y'\in Y\smallsetminus \{y\}$.

This is a standard exercise in applying Nielsen transformations. We outline the argument for the convenience of the reader.

### Proof

(Sketch proof) For $x_i\ne x_j\in X$, by replacing $x_i$ with $x_ix_j$ or $x_ix^{-1}_j$ and doing the corresponding replacement among the relations of $\mathcal {R}$ that contain $x_i$, we obtain a presentation $G=\langle X'\mid \mathcal {R}'\rangle$. We call the passage from $\langle X\mid \mathcal {R}\rangle$ to $\langle X'\mid \mathcal {R}'\rangle$ a *Nielsen transformation*. Let *K* (resp. $K'$) be the presentation complex of $\langle X\mid \mathcal {R}\rangle$ (resp. $\langle X'\mid \mathcal {R}'\rangle$). Then to pass from *K* to $K'$, one can subdivide the edge labeled by $x_i$ into two edges and identify one of these new edges with $x_j$ or $x^{-1}_j$. This process can be reversed, up to homotopy, and hence *K* and $K'$ are homotopy equivalent. Now, starting from $\langle X\mid \mathcal {R}\rangle$ and inductively performing Nielsen transformations, we can obtain the desired presentation, essentially performing Gaussian elimination in the $\mathbb {Z}$-module $G_{\textrm{fab}}$. $\square$

For the rest of this section, suppose *G* is a semi-direct product $H\rtimes \mathbb {Z}$ with *H* a type *F* subgroup. Let $\sigma :G\rightarrow {{\,\textrm{GL}\,}}_n(D)$ be a representation over a skew field *D*. Then *G* is $(\sigma \otimes _{\mathbb {Z}} q)$-acyclic by Proposition 3.8, and thus the agrarian norm $\Vert \cdot \Vert _{\sigma }$ is well defined. If ${{\,\textrm{rk}\,}}(G_{\textrm{fab}})=0$ then all agrarian norms are trivial. So below we assume that ${{\,\textrm{rk}\,}}(G_{\textrm{fab}})\geqslant 1$.

### Semi-norm property

#### Lemma 6.2

If $G_{\textrm{fab}}\cong \mathbb {Z}$ then $\Vert \cdot \Vert _{\sigma }$ is a semi-norm if and only if $\chi (H)\leqslant 0$.

#### Proof

The desired conclusion follows from Theorem 5.1 and Proposition 3.7. $\square$

Next, let us further suppose that *G* is *aspherical*, i.e., *G* has a finite presentation

15 $$\begin{aligned} G=\langle X\mid \mathcal {R}\rangle \end{aligned}$$

such that the corresponding presentation complex has contractible universal cover. By Lemma 6.1 we may assume that $X=\{x_i\}^{k+m}_{i=1}$ with $\{q(x_i)\}^k_{i=1}$ being a basis for $G_{\textrm{fab}}$ and $q(x_{k+1})=q(x_{k+2})=\cdots =q(x_{k+m})=0$.

#### Proposition 6.3

Suppose that *G* is a group that is (type *F*)-by-(infinite cyclic) and aspherical, and satisfies ${{\,\textrm{rk}\,}}G_{\textrm{fab}}\geqslant 2$. Then for every linear representation $\sigma :G\rightarrow {{\,\textrm{GL}\,}}_n(D)$ of *G* over a skew field *D*, the function $\Vert \cdot \Vert _{\sigma }$ is a semi-norm.

#### Remark 6.4

The above proposition is no longer true if ${{\,\textrm{rk}\,}}G_{\textrm{fab}}\geqslant 2$ is dropped. An easy example is given by $G=\mathbb {Z}$.

#### Proof

Let *K* be the presentation complex of (15). Consider the cellular chain complex of ${\widetilde{K}}$, the universal cover of *K*.
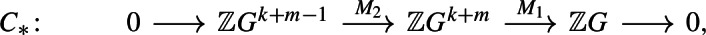


where $M_2$ is a $(k+m)\times (k+m-1)$ matrix over $\mathbb {Z}G$ and

$$\begin{aligned} M_1=(1-x_1,\cdots ,1-x_{k+m}). \end{aligned}$$

Note that we must have $\mathbb {Z}G^{k+m-1}$ in dimension 2 as $\chi (G)=0$ (see, e.g., Proposition 3.7 and Corollary 3.9). The corresponding maps are given by the left multiplication by $M_2$ and $M_1$.

Let
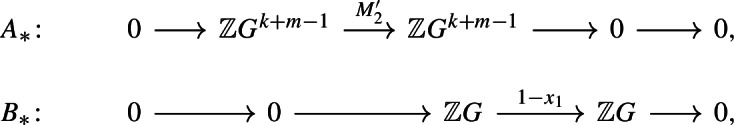


where $M'_2$ is the matrix obtained from $M_2$ by deleting the first row. We then have a short exact sequence of $\mathbb {Z}G$-chain complexes

16 $$\begin{aligned} 0\rightarrow A_{*}\rightarrow C_{*} \rightarrow B_{*} \rightarrow 0 \end{aligned}$$

that in every degree is split.

Let $Y=\{q(x_i)\}^k_{i=1}$. We identify ${{\,\textrm{Ore}\,}}(DG_{\textrm{fab}})$ with *D*(*Y*) to emphasize the role played by *Y*. By tensoring it with $(D(Y))_{\sigma }^n$ using the representation $\sigma \otimes _{\mathbb {Z}} q$, we obtain the sequence

17 $$\begin{aligned} 0\rightarrow A_{*}\otimes _{\mathbb {Z}G} (D(Y))_{\sigma }^n\rightarrow C_{*}\otimes _{\mathbb {Z}G} (D(Y))_{\sigma }^n \rightarrow B_{*}\otimes _{\mathbb {Z}G} (D(Y))_{\sigma }^n \rightarrow 0. \end{aligned}$$

Since the sequence (16) splits in every degree, the sequence (17) is exact.

Consider the unique non-zero differential of $B_{*}\otimes _{\mathbb {Z}G} (D(Y))_{\sigma }^n$, which is given by the left multiplication by the matrix

$$\begin{aligned}(\sigma \otimes _{\mathbb {Z}} q)(1-x_1)=\textrm{Id}+\sigma (-x_1)q(x_1).\end{aligned}$$

Every entry of $\sigma (-x_1)q(x_1)$ has $q(x_1)$-order at least 1. So Lemma 2.13 implies that $(\sigma \otimes _{\mathbb {Z}} q)(1-x_1)$ is invertible, and thus the chain complex $B_{*}\otimes _{\mathbb {Z}G} (D(Y))_{\sigma }^n$ is exact. This, together with the exactness of $C_{*}\otimes _{\mathbb {Z}G}(D(Y))_{\sigma }^n$ (by Corollary 3.9), yields that $A_{*}\otimes _{\mathbb {Z}G} (D(Y))_{\sigma }^n$ is also exact.

Let $\rho _A$ (resp. $\rho _B,\rho _C$) be the Reidemeister torsion of $A_{*}\otimes _{\mathbb {Z}G} (D(Y))_{\sigma }^n$ (resp. $B_{*}\otimes _{\mathbb {Z}G} (D(Y))_{\sigma }^n,C_{*}\otimes _{\mathbb {Z}G} (D(Y))_{\sigma }^n$). Then (see, e.g., [7, (17.2)])

18 $$\begin{aligned} \rho _C=\rho _A\cdot \rho _B. \end{aligned}$$

Let $P:D(Y)\rightarrow \mathcal {P}(G_{\textrm{fab}})$ be the polytope homomorphism. Equation (18) implies

19 $$\begin{aligned} P(\rho _C)=P({{\,\mathrm{\det }\,}}_{D(Y)} (\sigma \otimes _{\mathbb {Z}} q)(M'_2))-P({{\,\mathrm{\det }\,}}_{D(Y)}(\sigma \otimes _{\mathbb {Z}} q)(1-x_1)). \end{aligned}$$

Since $M'_2$ is a square matrix over $\mathbb {Z}G$, $(\sigma \otimes _{\mathbb {Z}} q)(M'_2)$ is a square matrix over $D G_{\textrm{fab}}$. By Theorem 2.12,

$$\begin{aligned}P({{\,\mathrm{\det }\,}}_{D(Y)} (\sigma \otimes _{\mathbb {Z}} q)(M'_2))\in P(D[Y^{\pm }])\end{aligned}$$

is a single polytope, and thus (19) implies

20 $$\begin{aligned} P(\rho _C)\in P(D[Y^{\pm }])-P(D[x^{\pm }_1]). \end{aligned}$$

By the assumption ${{\,\textrm{rk}\,}}G_{\textrm{fab}}\geqslant 2$ we also have $q(x_2)\in Y$. The above argument with $x_2$ in place of $x_1$ yields

$$\begin{aligned}P(\rho _C)\in P(D[Y^{\pm }])-P(D[x^{\pm }_2]),\end{aligned}$$

which together with (20) implies that $P(\rho _C)$ is a single polytope, which by Lemma 3.13 yields the desired result. $\square$

### Inequality between the Alexander and Thurston norms

The inequality between the Alexander and Thurston norms for 3-manifolds was discovered by McMullen [41], whose result was then generalized by Friedl–Kim [12] and Funke and the first author [13]. In the current and subsequent sections, we recover the result of [12] and generalize the result of [13].

#### Theorem 6.5

Suppose that *G* is an aspherical (Lewin type *F*)-by-(infinite cyclic) group. Let $\Vert \cdot \Vert _T$ be the Thurston norm of *G*. Then for all finite dimensional complex representations $\sigma :G\rightarrow {{\,\textrm{GL}\,}}_n(\mathbb {C})$ and all $\phi \in H^1(G,\mathbb {Z})$, one has

$$\begin{aligned} \Vert \phi \Vert _{\sigma }\leqslant n\cdot \Vert \phi \Vert _T. \end{aligned}$$

Moreover, if $\ker \phi$ is of type *F* then we have

21 $$\begin{aligned} \Vert \phi \Vert _{\sigma }=-n\cdot \chi (\ker \phi )=n\cdot \Vert \phi \Vert _T. \end{aligned}$$

#### Proof

Without loss of generality, we may assume that $\phi$ is a primitive integral character. By Lemma 6.1, there is a finite presentation

$$\begin{aligned}G=\langle x_1,\cdots ,x_{k+m}\mid \mathcal {R}\rangle \end{aligned}$$

such that

(i) $Y=\{q(x_i)\}^k_{i=1}$ is a basis for $G_{\textrm{fab}}$;
(ii) $$q(x_{k+1})=q(x_{k+2})=\cdots =q(x_{k+m})=0$$;
(iii) $$\phi (x_1)=1$$ and $$\phi (x_2)=\phi (x_3)=\cdots =\phi (x_k)=0$$.

Let $\mathcal {D}_G$ be the Linnell skew field of *G* and let $\tau :\mathbb {C}G\rightarrow \mathcal {D}_G$ be the natural embedding. We identify ${{\,\textrm{Ore}\,}}(\mathbb {C}G_{\textrm{fab}})$ (resp. ${{\,\textrm{Ore}\,}}(\mathcal {D}_G G_{\textrm{fab}})$) with $\mathbb {C}(Y)$ (resp. $\mathcal {D}_G(Y)$) to emphasize the role played by *Y*. For simplicity, we also denote $q(x_1)$ by *s*. Equation (19) implies

22 $$\begin{aligned} \Vert \phi \Vert _{\sigma }=\deg _s{{\,\mathrm{\det }\,}}_{\mathbb {C}(Y)} (\sigma \otimes _{\mathbb {Z}} q)(M'_2)-\deg _s{{\,\mathrm{\det }\,}}_{\mathbb {C}(Y)} (\sigma \otimes _{\mathbb {Z}} q)(1-x_1). \end{aligned}$$

Similarly,

23 $$\begin{aligned} \Vert \phi \Vert _T =\deg _s{{\,\mathrm{\det }\,}}_{\mathcal {D}_G(Y)} (\tau \otimes _{\mathbb {Z}} q)(M'_2)-\deg _s{{\,\mathrm{\det }\,}}_{\mathcal {D}_G(Y)} (\tau \otimes _{\mathbb {Z}} q)(1-x_1), \end{aligned}$$

Since *G* is Lewin [25, Theorem 3.7 (3)] and the skew fields considered here are $\mathbb {C}$ and $\mathcal {D}_G$, Lemma 2.27 implies

24 $$\begin{aligned} \deg _s{{\,\mathrm{\det }\,}}_{\mathbb {C}(Y)} (\sigma \otimes _{\mathbb {Z}} q)(M'_2) \leqslant \deg _s{{\,\mathrm{\det }\,}}_{\mathcal {D}_G(Y)} (\sigma \otimes _{\mathbb {C}}\tau \otimes _{\mathbb {Z}} q)(M'_2). \end{aligned}$$

Consider the representation $\sigma \otimes _{\mathbb {C}}\tau :G\rightarrow \textrm{GL}_n(\mathcal {D}_G)$. Theorem 4.3 extends $\sigma \otimes _{\mathbb {C}}\tau$ to a ring homomorphism $\widetilde{\sigma }:\mathcal {D}_G\rightarrow M_n(\mathcal {D}_G)$. By repeatedly using Corollary 2.15, we further extends $\widetilde{\sigma }$ to ring homomorphism $\widetilde{\sigma }:\mathcal {D}_G(Y)\rightarrow M_n(\mathcal {D}_G(Y))$ such that $\widetilde{\sigma }(y)=\textrm{Id}\cdot y$ for all $y\in Y$. We have

$$\begin{aligned}\widetilde{\sigma }\big ((\tau \otimes _{\mathbb {Z}}q)(M'_2)\big )=(\sigma \otimes _{\mathbb {C}}\tau \otimes _{\mathbb {Z}} q)(M'_2).\end{aligned}$$

Lemma 2.14 thus implies

25 $$\begin{aligned} \deg _s{{\,\mathrm{\det }\,}}_{\mathcal {D}_G(Y)} (\sigma \otimes _{\mathbb {C}}\tau \otimes _{\mathbb {Z}} q)(M'_2)=n\cdot \deg _s{{\,\mathrm{\det }\,}}_{\mathcal {D}_G(Y)} (\tau \otimes _{\mathbb {Z}} q)(M'_2). \end{aligned}$$

Think of ${{\,\mathrm{\det }\,}}_{\mathbb {C}(Y)}(\sigma \otimes _{\mathbb {Z}} q)(1-x_1)$ as a polynomial in *s* with coefficient in ${\mathbb {C}(Y\smallsetminus \{s\})}$. Then the highest power of *s* in ${{\,\mathrm{\det }\,}}_{\mathbb {C}(Y)}(\sigma \otimes _{\mathbb {Z}} q)(1-x_1)$ is $s^n$ with coefficient ${{\,\mathrm{\det }\,}}_{\mathbb {C}(Y)}\sigma (-x_1)$. The lowest power of *s* in ${{\,\mathrm{\det }\,}}_{\mathbb {C}(Y)}(\sigma \otimes _{\mathbb {Z}} q)(1-x_1)$ is $s^0=1$ with coefficient 1. Thus,

26 $$\begin{aligned} \deg _s{{\,\mathrm{\det }\,}}_{\mathbb {C}(Y)} (\sigma \otimes _{\mathbb {Z}} q)(1-x_1)=n=n\cdot \deg _s{{\,\mathrm{\det }\,}}_{\mathcal {D}_G(Y)} (\tau \otimes _{\mathbb {Z}} q)(1-x_1). \end{aligned}$$

We conclude from (22), (23), (24), (25) and (26) that

$$\begin{aligned}\Vert \phi \Vert _{\sigma }\leqslant \Vert \phi \Vert _{\sigma \otimes _{\mathbb {C}}\tau }=n\cdot \Vert \phi \Vert _T.\end{aligned}$$

If $\ker \phi$ is of type *F*, then by Theorem 5.1,

27 $$\begin{aligned} \Vert \phi \Vert _{\sigma }=-\chi ^{\sigma \otimes _{\mathbb {Z}}q}(\ker \phi ). \end{aligned}$$

By Proposition 3.7, we have

28 $$\begin{aligned} \chi ^{\sigma \otimes _{\mathbb {Z}}q}(\ker \phi )=n\cdot \chi (\ker \phi ). \end{aligned}$$

and

29 $$\begin{aligned} \Vert \phi \Vert _T=-\chi ^{\tau }(\ker \phi ). \end{aligned}$$

By Theorem 5.1 again, we have

30 $$\begin{aligned} \chi ^{\tau }(\ker \phi )=\chi (\ker \phi ). \end{aligned}$$

Equation (21) follows by combining (27), (28), (29) and (30). $\square$

## Application to free-by-cyclic and 3-manifold groups

### Free-by-cyclic groups

Let *G* be a (finitely generated free)-by-(infinite cyclic) group. Then *G* is locally indicable. In particular, the Thurston norm $\Vert \cdot \Vert _T$ of *G* is well defined.

#### Theorem 7.1

For any (finitely generated free)-by-(infinite cyclic) group *G* and any representation $\sigma :G\rightarrow {{\,\textrm{GL}\,}}_n(D)$, the function $\Vert \cdot \Vert _{\sigma }$ is a semi-norm.

Moreover, if $D=\mathbb {C}$ is the field of complex numbers, then for every $\phi \in H^1(G,\mathbb {Z})$,

31 $$\begin{aligned} \Vert \phi \Vert _{\sigma }\leqslant n\cdot \Vert \phi \Vert _T \end{aligned}$$

and equality holds when $\phi$ is a fibered character, i.e., when $\ker \phi$ is finitely generated.

#### Proof

First, suppose $G_{\textrm{fab}}=\mathbb {Z}$. Let $\phi \in H^1(G,\mathbb {Z})$ be the unique (up to sign) primitive integral character. Then $\ker \phi$ is a finitely generated free group, and thus is of type *F* and satisfies $\chi (\ker \phi )\leqslant 0$. That $\Vert \cdot \Vert _{\sigma }$ is a semi-norm follows from Lemma 6.2. Theorem 6.5 implies that

$$\begin{aligned}\Vert \phi \Vert _{\sigma }=-n\cdot \chi (\ker \phi )=n\cdot \Vert \phi \Vert _T.\end{aligned}$$

Suppose ${{\,\textrm{rk}\,}}G_{\textrm{fab}}\geqslant 2$. Since *G* is aspherical (see, e.g., [13, Lemma 3.1]), Proposition 6.3 implies that $\Vert \cdot \Vert _{\sigma }$ is a semi-norm. Since every finitely generated subgroup of *G* is of type *F* [11], a character $\phi \in H^1(G,\mathbb {Z})$ is fibered if and only if $\ker \phi$ is of type *F*. Moreover, by [25, Theorem 1.1 and Theorem 3.7 (2)], *G* is (Lewin type *F*)-by-(infinite cyclic). Inequality (31), as well as the equality for fibered characters, follows from Theorem 6.5. $\square$

### 3-manifold groups

Let *G* be the fundamental group of a closed connected orientable 3-manifold *M* that fibers over $S^1$. Then *G* fits into a short exact sequence

$$\begin{aligned} 1\rightarrow \pi _1(S)\rightarrow G \rightarrow \mathbb {Z}\rightarrow 1.\end{aligned}$$

By Example 2.17, $\pi _1(S)$ and $\mathbb {Z}$ are locally indicable, and thus *G* is locally indicable by Lemma 2.18. In particular, the Thurston norm $\Vert \cdot \Vert _T$ of *G* is well defined. The goal of this subsection is the following.

#### Theorem 7.2

Suppose that *G* is the fundamental group of a closed connected orientable 3-manifold *M* that fibers over $S^1$. Then for any representation $\sigma :G\rightarrow {{\,\textrm{GL}\,}}_n(D)$ of *G* over a skew field *D*, the agrarian norm $\Vert \cdot \Vert _{\sigma }$ is well defined. Moreover,

(i) if $M\ne S^1\times S^2$, then $\Vert \cdot \Vert _{\sigma }$ is a semi-norm;
(ii) if $D=\mathbb {C}$, then for every $\phi \in H^1(G,\mathbb {Z})$,
  32 $$\begin{aligned} \Vert \phi \Vert _{\sigma }\leqslant n\cdot \Vert \phi \Vert _T \end{aligned}$$
  where$\Vert \cdot \Vert _T$ is the Thurston norm of *G*. Moverover, if $\phi$ is a fibered character, then
  33 $$\begin{aligned} \Vert \phi \Vert _{\sigma }= n\cdot \Vert \phi \Vert _T. \end{aligned}$$

#### Proof

There is a closed surface *S* such that *M* decomposes as a fiber bundle $S\rightarrow M\rightarrow S^1$. In particular, *M* is a mapping torus of a cellular self map of a finite connected CW-complex. That $\Vert \cdot \Vert _{\sigma }$ is well defined thus follows from Proposition 3.8.

We have $G=\pi _1(S)\rtimes \mathbb {Z}$. If ${{\,\textrm{rk}\,}}G_{\textrm{fab}}=1$, then items (i) and (ii)follow from Theorem 5.1 and Lemma 6.2. Below we assume ${{\,\textrm{rk}\,}}G_{\textrm{fab}}\geqslant 2$.

Let ${\widetilde{M}}$ be the universal cover of *M*. By the proof of [41, Theorem 5.1], ${\widetilde{M}}$ has a *G*-equivariant CW structure whose cellular chain complex has the form
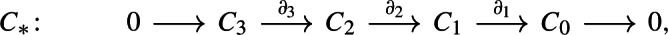


where there are $\mathbb {Z}G$-bases $\{p\}$ of $C_0$, $\{e_i\}^k_{i=1}$ of $C_1$, $\{f_i\}^k_{i=1}$ of $C_2$, $\{t\}$ of $C_3$, and there is a generating set $\{g_i\}^k_{i=1}$ of *G* with the following properties:

(i) $$\partial _1(e_i)=p\cdot (1-g_i),\, \partial _3(t)=\sum ^k_{j=1} f_j\cdot (1-g_j)$$.
(ii) $X=\{q(g_i)\}^m_{i=1}$ is a basis of $G_{\textrm{fab}}$ for some $m\leqslant k$, and $q(g_i)=0$ for $i>m$, where $q:G\rightarrow G_{\textrm{fab}}$ is the natural surjection of *G* onto its maximal free abelian quotient $G_{\textrm{fab}}$.

We denote the matrix representative of $\partial _{*}$ under the above bases by $[\partial _{*}]$.

Consider the chain complexes
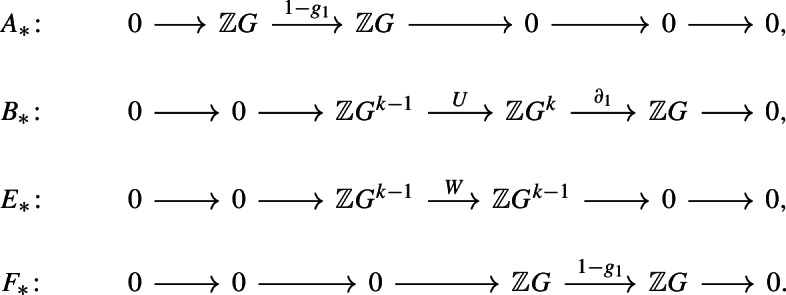


Here, *U* is the matrix obtained from the matrix $[\partial _2]$ by deleting the first column, and *W* is obtained from *U* by deleting the first row.

We then have exact sequences of chain complexes

$$\begin{aligned} 0\rightarrow A_{*} \rightarrow C_{*}\rightarrow B_{*} \rightarrow 0, \\ 0\rightarrow E_{*} \rightarrow B_{*} \rightarrow F_{*} \rightarrow 0, \end{aligned}$$

that in each degree are split.

Let $\sigma :G\rightarrow {{\,\textrm{GL}\,}}_n(D)$ be a representation over a skew field *D*. Recall that $X=\{q(g_i)\}^m_{i=1}$ is a basis of $G_{\textrm{fab}}$. As before we denote ${{\,\textrm{Ore}\,}}(DG_{\textrm{fab}})$ by *D*(*X*) to emphasize the role played by *X*. Below, the $q(g_1)$-order will be taken with respect to *X*.

Upon tensoring with $(D(X))_{\sigma }^n$ via $\sigma \otimes _{\mathbb {Z}} q$, they become the following sequences

34 $$\begin{aligned} 0\rightarrow A_{*}\otimes _{\mathbb {Z}G}(D(X))_{\sigma }^n \rightarrow C_{*}\otimes _{\mathbb {Z}G}(D(X))_{\sigma }^n\rightarrow B_{*}\otimes _{\mathbb {Z}G}(D(X))_{\sigma }^n \rightarrow 0, \end{aligned}$$

35 $$\begin{aligned} 0\rightarrow E_{*}\otimes _{\mathbb {Z}G}(D(X))_{\sigma }^n \rightarrow B_{*}\otimes _{\mathbb {Z}G}(D(X))_{\sigma }^n \rightarrow F_{*}\otimes _{\mathbb {Z}G}(D(X))_{\sigma }^n \rightarrow 0. \end{aligned}$$

Both sequences are exact, since the original sequences split in every degree.

Consider the unique non-zero differentials in $A_{*}\otimes _{\mathbb {Z}G}(D(X))_{\sigma }^n$ and $F_{*}\otimes _{\mathbb {Z}G}(D(X))_{\sigma }^n$, which are given by the left multiplication by the matrix

$$\begin{aligned}(\sigma \otimes _{\mathbb {C}}\tau )(1-g_1)=\textrm{Id}+\sigma (-g_1)q(g_1).\end{aligned}$$

Note that every entry of $\sigma (-g_1)q(g_1)$ has $q(g_1)$-order at least 1. So Lemma 2.13 implies that $(\sigma \otimes _{\mathbb {C}}\tau )(1-g_1)$ is invertible, which in turn implies that $A_{*}\otimes _{\mathbb {Z}G}(D(X))_{\sigma }^n$ and $F_{*}\otimes _{\mathbb {Z}G}(D(X))_{\sigma }^n$ are exact. Since *G* is (type *F*)-by-(infinite cyclic), Corollary 3.9 implies that $C_{*}\otimes _{\mathbb {Z}G}(D(X))_{\sigma }^n$ is exact. It follows that $B_{*}\otimes _{\mathbb {Z}G}(D(X))_{\sigma }^n$ is also exact, which in turn implies that $E_{*}\otimes _{\mathbb {Z}G}(D(X))_{\sigma }^n$ is also exact.

Let $\rho _A$ (resp. $\rho _B,\rho _C,\rho _E,\rho _F$) be the Reidemeister torsion of $A_{*}\otimes _{\mathbb {Z}G} (D(X))_{\sigma }^n$ (resp. $B_{*}\otimes _{\mathbb {Z}G} (D(X))_{\sigma }^n, C_{*}\otimes _{\mathbb {Z}G} (D(X))_{\sigma }^n,E_{*}\otimes _{\mathbb {Z}G} (D(X))_{\sigma }^n,F_{*}\otimes _{\mathbb {Z}G} (D(X))_{\sigma }^n$). Then (see, e.g., [7, (17.2)])

$$\begin{aligned} \rho _C=\rho _A \cdot \rho _B = \rho _A \cdot \rho _E \cdot \rho _F = {{\,\mathrm{\det }\,}}_{D(X)} (\sigma \otimes _{\mathbb {Z}} q)(W) \cdot ({{\,\mathrm{\det }\,}}_{D(X)} (\textrm{Id}-\sigma (g_1)q(g_1)))^{-2}. \end{aligned}$$

Let $P:D(X)\rightarrow \mathcal {P}(G_{\textrm{fab}})$ be the polytope homomorphism. Since *W* is a matrix over $\mathbb {Z}G$, Theorem 2.12 implies that

$$\begin{aligned} P({{\,\mathrm{\det }\,}}_{D(X)} (\sigma \otimes _{\mathbb {Z}} q)(W))\in P(D[X^{\pm }]) \end{aligned}$$

is a single polytope. Therefore,

$$\begin{aligned}P(\rho _C)\in P(D[X^{\pm }])-P(D[q(g_1)^{\pm }]).\end{aligned}$$

We have assumed that ${{\,\textrm{rk}\,}}G_{\textrm{fab}}\geqslant 2$. In particular, $q(g_2)\ne 0$. The above argument with $g_2$ in place of $g_1$ yields that

$$\begin{aligned}P(\rho _C)\in P(D[X^{\pm }])-P(D[q(g_2)^{\pm }])\end{aligned}$$

and thus $P(\rho _C)$ is a single polytope, which means that $\Vert \cdot \Vert _{\sigma }$ is a semi-norm.

We proceed to prove item (ii). Suppose that $D=\mathbb {C}$ is the field of complex numbers. Let $\phi \in H^1(G,\mathbb {Z})\smallsetminus \{0\}$. Without loss of generality, we may assume that $\phi$ is a primitive integral character. Let *Y* be a basis of $G_{\textrm{fab}}$ such that there is $s\in Y$ with $\phi (s)=1$ and $\phi (y)=0$ for all $y\in Y\smallsetminus \{s\}$. For any right $\mathbb {Z}G$-module *N*, $N\otimes _{\mathbb {Z}G}(D(X))_{\sigma }^n$ and $N\otimes _{\mathbb {Z}G}(D(Y))_{\sigma }^n$ are naturally isomorphic. Thus, we obtain the following exact sequences from (34) and (35)

$$\begin{aligned} 0\rightarrow A_{*}\otimes _{\mathbb {Z}G}(\mathbb {C}(Y))_{\sigma }^n \rightarrow C_{*}\otimes _{\mathbb {Z}G}(\mathbb {C}(Y))_{\sigma }^n\rightarrow B_{*}\otimes _{\mathbb {Z}G}(\mathbb {C}(Y))_{\sigma }^n \rightarrow 0,\\ 0\rightarrow E_{*}\otimes _{\mathbb {Z}G}(\mathbb {C}(Y))_{\sigma }^n \rightarrow B_{*}\otimes _{\mathbb {Z}G}(\mathbb {C}(Y))_{\sigma }^n \rightarrow F_{*}\otimes _{\mathbb {Z}G}(\mathbb {C}(Y))_{\sigma }^n \rightarrow 0, \end{aligned}$$

which imply that

36 $$\begin{aligned} \Vert \phi \Vert _{\sigma }=\deg _s{{\,\mathrm{\det }\,}}_{\mathbb {C}(Y)} (\sigma \otimes _{\mathbb {Z}} q)(W) - 2\cdot \deg _s{{\,\mathrm{\det }\,}}_{\mathbb {C}(Y)} (\textrm{Id}-\sigma (g_1)q(g_1)). \end{aligned}$$

Let $\tau :\mathbb {C}G\rightarrow \mathcal {D}_G$ be the natural embedding of $\mathbb {C}G$ into its Linnell skew field. The same argument with $\tau$ in place of $\sigma$ yields

37 $$\begin{aligned} \Vert \phi \Vert _T=\deg _s{{\,\mathrm{\det }\,}}_{\mathcal {D}_G(Y)} (\tau \otimes _{\mathbb {Z}} q)(W) - 2\cdot \deg _s{{\,\mathrm{\det }\,}}_{\mathcal {D}_G(Y)} (\textrm{Id}-\tau (g_1)q(g_1)). \end{aligned}$$

We prove that

38 $$\begin{aligned} \deg _s{{\,\mathrm{\det }\,}}_{\mathbb {C}(Y)} (\textrm{Id}-\sigma (g_1)q(g_1))=n\cdot \deg _s{{\,\mathrm{\det }\,}}_{\mathcal {D}_G(Y)} (\textrm{Id}-\tau (g_1)q(g_1)). \end{aligned}$$

Write $q(g_1)$ as a monomial in *Y*:

$$\begin{aligned}q(g_1)=s^r\cdot \prod _{y\in Y\smallsetminus \{s\}}y^{r_y}, \end{aligned}$$

where we use multiplicative notation for the abelian group $G_{\textrm{fab}}$.

First consider the case $r=0$. Then $\deg _s{{\,\mathrm{\det }\,}}_{\mathbb {C}(Y)} (\textrm{Id}-\sigma (g_1)q(g_1))$ is either 0 or $-\infty$. We have shown that $\textrm{Id}-\sigma (g_1)q(g_1)$ is invertible, so

$$\begin{aligned}\deg _s{{\,\mathrm{\det }\,}}_{\mathbb {C}(Y)} (\textrm{Id}-\sigma (g_1)q(g_1))=0.\end{aligned}$$

Clearly, $\deg _s{{\,\mathrm{\det }\,}}_{\mathcal {D}_G(Y)} (\textrm{Id}-\tau (g_1)q(g_1))=0$. Thus, (38) holds in this case.

Consider the case $r\ne 0$. Without loss of generality we may assume $r>0$ (the case $r<0$ can be analyzed in the same way). Think of

$$\begin{aligned}{{\,\mathrm{\det }\,}}_{\mathbb {C}(Y)} (\textrm{Id}-\sigma (g_1)q(g_1))\end{aligned}$$

as a polynomial in *s* with coefficients in $\mathbb {C}(Y\smallsetminus \{s\})$. Then the highest power of *s* in ${{\,\mathrm{\det }\,}}_{\mathbb {C}(Y)} (\textrm{Id}-\sigma (g_1)q(g_1))$ is $s^{nr}$ with coefficient ${{\,\mathrm{\det }\,}}_{\mathbb {C}}\sigma (g_1)\cdot \prod _{y\in Y\smallsetminus \{s\}}y^{nr_y}$. The lowest power of *s* in ${{\,\mathrm{\det }\,}}_{\mathbb {C}(Y)} (\textrm{Id}-\sigma (g_1)q(g_1))$ is $s^0=1$ with coefficient 1. Thus, $\deg _s{{\,\mathrm{\det }\,}}_{\mathbb {C}(Y)} (\textrm{Id}-\sigma (g_1)q(g_1))=nr$. Since $\deg _s{{\,\mathrm{\det }\,}}_{\mathcal {D}_G(Y)} (\textrm{Id}-\tau (g_1)q(g_1))=r$, equation (38) also holds in this case.

Theorem 4.3 extends the representation $\sigma \otimes _{\mathbb {C}}\tau :G\rightarrow \textrm{GL}_n(\mathcal {D}_G)$ to a ring homomorphism $\widetilde{\sigma }:\mathcal {D}_G\rightarrow M_n(\mathcal {D}_G)$. By repeatedly using Corollary 2.15, we further extend $\widetilde{\sigma }$ to a ring homomorphism (still denoted by) $\widetilde{\sigma }:\mathcal {D}_G(Y)\rightarrow M_n(\mathcal {D}_G(Y))$ such that $\widetilde{\sigma }(y)=y\cdot \textrm{Id}$ for all $y\in Y$. Then

$$\begin{aligned}\widetilde{\sigma }((\tau \otimes _{\mathbb {Z}}q)(W))=(\sigma \otimes _{\mathbb {C}}\tau \otimes _{\mathbb {Z}}q)(W).\end{aligned}$$

Note that $\pi _1(S)$ is Lewin. Indeed, there is a homomorphism $\pi _1(S)\rightarrow \mathbb {Z}$ whose kernel is the fundamental group of a non-compact surface, and thus is free. Therefore, $\pi _1(S)$ is a semi-direct product of a free group with $\mathbb {Z}$. It follows that $\pi _1(S)$ is Lewin and thus so is *G* [25, Theorems 1.1 and 3.7].

Lemmata 2.27 and 2.14 then imply

$$\begin{aligned} \deg _s{{\,\mathrm{\det }\,}}_{\mathbb {C}(Y)} (\sigma \otimes _{\mathbb {Z}} q)(W)\leqslant&\deg _s{{\,\mathrm{\det }\,}}_{\mathcal {D}_G(Y)} (\sigma \otimes _{\mathbb {C}}\tau \otimes _{\mathbb {Z}} q)(W)\\ =&n\cdot \deg _s{{\,\mathrm{\det }\,}}_{\mathcal {D}_G(Y)} (\tau \otimes _{\mathbb {Z}} q)(W), \end{aligned}$$

which, together with (36), (37) and (38) finishes the proof of (32).

If $\phi$ is a fibered character, then $\ker \phi$ is the fundamental group of some closed surface [45] and thus is of type *F*. Theorem 5.1 then implies that

39 $$\begin{aligned} \Vert \phi \Vert _{\sigma }=-\chi ^{\sigma \otimes _{\mathbb {Z}}q}(\ker \phi ). \end{aligned}$$

By Proposition 3.7, we have

40 $$\begin{aligned} \chi ^{\sigma \otimes _{\mathbb {Z}}q}(\ker \phi )=n\cdot \chi (\ker \phi ). \end{aligned}$$

and

41 $$\begin{aligned} \Vert \phi \Vert _T=-\chi ^{\tau }(\ker \phi ). \end{aligned}$$

By Theorem 5.1 again, we have

42 $$\begin{aligned} \chi ^{\tau }(\ker \phi )=\chi (\ker \phi ). \end{aligned}$$

Equation (33) follows by combining (39), (40), (41) and (42). $\square$

## Data Availability

Data sharing not applicable to this article as no datasets were generated or analysed during the current study.

## References

[1] Aschenbrenner, M., Friedl, S., Wilton, H.: 3-manifold groups, EMS Series of Lectures in Mathematics, European Mathematical Society (EMS), Zürich, (2015)

[2] Agol, I.: The virtual Haken conjecture, Doc. Math. 18 (2013), 1045–1087. With an appendix by I. Agol, D. Groves, and J. Manning

[3] Atiyah, M.F.: Elliptic operators, discrete groups and von Neumann algebras, Colloque “Analyse et Topologie” en l’onneur de Henri Cartan (Orsay, 1974), 1976, pp. 43–72. Astérisque, No. 32–33

[4] Boschheidgen, J., Jaikin-Zapirain, A.: Twisted -Betti numbers of sofic groups, arXiv:2201.03268 (2022)

[5] Borel, A.: The -cohomology of negatively curved Riemannian symmetric spaces, Annales Academiae Scientiarum Fennicae. Series A I. Mathematica **10**, 95–105 (1985)

[6] Chiswell, I.: Exact sequences associated with a graph of groups. J. Pure Appl. Algebra **8**(1), 63–74 (1976)

[7] Cohen, M.M.: A course in simple-homotopy theory, Springer-Verlag, New York- Berlin, 1973. Graduate Texts in Mathematics, **10**

[8] Dicks, W., Herbera, D., Sánchez, J.: On a theorem of Ian Hughes about division rings of fractions. Comm. Algebra **32**(3), 1127–1149 (2004)

[9] Dieudonné, J.: Les déterminants sur un corps non commutatif. Bull. Soc. Math. France **71**, 27–45 (1943)

[10] Dodziuk, J.: harmonic forms on rotationally symmetric Riemannian manifolds. Proc. Am. Math. Soc. **77**(3), 395–400 (1979)

[11] Feighn, M., Handel, M.: Mapping tori of free group automorphisms are coherent. Ann. Math. **149**, 1061–1077 (1999)

[12] Friedl, S., Kim, T.: Twisted Alexander norms give lower bounds on the Thurston norm. Trans. Amer. Math. Soc. **360**(9), 4597–4618 (2008)

[13] Funke, F., Kielak, D.: Alexander and Thurston norms, and the Bieri- Neumann-Strebel invariants for free-by-cyclic groups. Geom. Topol. **22**(5), 2647–2696 (2018)

[14] Friedl, S., Lück, W.: Universal -torsion, polytopes and applications to 3-manifolds. Proc. Lond. Math. Soc. **114**(6), 1114–1151 (2017)

[15] Friedl, S., Lück, W.: -Euler characteristics and the Thurston norm. Proc. Lond. Math. Soc. **118**(4), 857–900 (2019)

[16] Friedl, S., Vidussi, S.: Symplectic , subgroup separability, and vanishing Thurston norm. J. Am. Math. Soc. **21**(2), 597–610 (2008)

[17] Friedl, S., Vidussi, S.: A survey of twisted Alexander polynomials. Math. Knots, 45–94 (2011)

[18] Groves, D., Manning, J., Wilton, H.: Recognizing geometric 3-manifold groups using the word problem, arXiv:1210.2101 (2012)

[19] Henneke, F., Kielak, D.: Agrarian and -invariants, arXiv:1809.08470, to appear in Fund. Math. (2018)

[20] Hopf, H.: Differentialgeometrie und topologische gestalt. Jahresber. Dtsch. Math.-Ver. **41**, 209–229 (1932)

[21] Howie, J.: On locally indicable groups. Math. Z. **180**(4), 445–461 (1982)

[22] Howie, J., Short, H.: The band-sum problem. J. London Math. Soc. **31**(3), 571–576 (1985)

[23] Hughes, I.: Division rings of fractions for group rings. Comm. Pure Appl. Math. **23**, 181–188 (1970)

[24] Jaikin-Zapirain, A.: Recognition of being fibered for compact 3-manifolds. Geom. Topol. **24**(1), 409–420 (2020)

[25] Jaikin-Zapirain, A.: The universality of Hughes-free division rings. Selecta Math. (N.S.) **27(4)**, Paper No. 74, 33, (2021)

[26] Jaikin-Zapirain, A., López-Álvarez, D.: The strong Atiyah and Lück approximation conjectures for one-relator groups. Math. Ann. **376**(3–4), 1741–1793 (2020)

[27] Kielak, D.: The Bieri-Neumann-Strebel invariants via Newton polytopes. Invent. Math. **219**(3), 1009–1068 (2020)

[28] Kielak, D.: Residually finite rationally solvable groups and virtual fibring. J. Amer. Math. Soc. **33**(2), 451–486 (2020)

[29] Problems in low-dimensional topology, Geometric topology (Athens, GA, 1993), 1997, pp. 35–473

[30] Kapovich, M., Leeb, B.: 3-manifold groups and nonpositive curvature. Geom. Funct. Anal. **8**(5), 841–852 (1998)

[31] Kirk, P., Livingston, C.: Twisted knot polynomials: inversion, mutation and concordance. Topology **38**(3), 663–671 (1999)

[32] Leeb, B.: 3-manifolds with(out) metrics of nonpositive curvature. Invent. Math. **122**(2), 277–289 (1995)

[33] Levi, F.W.: Ordered groups, Proc. Indian Acad. Sci., Sect. A. **16**, 256– 263, (1942)

[34] Lin, X.: Representations of knot groups and twisted Alexander polynomials, Acta Math. Sin. (Engl. Ser.) **17(3)**, 361–380, (2001)

[35] Linnell, P.: Division rings and group von Neumann algebras. Forum Math. **5**(6), 561–576 (1993)

[36] Liu, Y.: Finite-volume hyperbolic 3-manifolds are almost determined by their finite quotient groups (2020)

[37] Lott, J., Lück, W.: -topological invariants of 3-manifolds. Invent. Math. **120**(1), 15–60 (1995)

[38] Lück, W.: -invariants: theory and applications to geometry and K-theory, Ergebnisse der Mathematik und ihrer Grenzgebiete. 3. Folge. A Series of Modern Surveys in Mathematics [Results in Mathematics and Related Areas. 3rd Series. A Series of Modern Surveys in Mathematics], **44**, Springer-Verlag, Berlin, (2002)

[39] Lück, W.: Twisting -invariants with finite-dimensional representations. J. Topol. Anal. **10**(4), 723–816 (2018)

[40] Mal’ cev, A.: On the embedding of group algebras in division algebras, Doklady Akad. Nauk SSSR (N.S.) **60**, 1499.1501, (1948)

[41] McMullen, C.: The Alexander polynomial of a 3-manifold and the Thurston norm on cohomology, Ann. Sci. École Norm. Sup. (4) **35(2)**, 153– 171, (2002)

[42] Olbrich, M.: -invariants of locally symmetric spaces. Docum. Math. **7**, 219–237 (2002)

[43] Passman, D.S.: The algebraic structure of group rings, Robert E. Krieger Publishing Co., Inc., Melbourne, FL, 1985. Reprint of the 1977 original

[44] Rosenberg, J.: Algebraic K-theory and its applications, Graduate Texts in Mathematics, vol. 147. Springer-Verlag, New York (1994)

[45] Stallings, J.: On fibering certain 3-manifolds, topology of 3-manifolds and related topics. Proc Univ. Georgia Instit. **1962**, 95–100 (1961)

[46] Tamari, D.: A refined classification of semi-groups leading to generalized polynomial rings with a generalized degree concept, Proc. Int. Cong. of Math.– 1954, Amsterdam **3**, 439–440 (1954)

[47] Thurston, W.: Hyperbolic structures on 3-manifolds. I. Deformation of acylindrical manifolds. Ann. Math. (2) **124(2)**, 203–246 (1986)

[48] Thurston, W.: A norm for the homology of 3-manifolds. Mem. Amer. Math. Soc. 59, no. 339, i–vi and 99–130 (1986)

[49] Wada, M.: Twisted Alexander polynomial for finitely presentable groups. Topology **33**(2), 241–256 (1994)

[50] Yau, S.T.: Problem section, Seminar on Differential Geometry, pp. 669–706, (1982)

